# Multivariate analysis of metabolomic data to identify biological pathways modified by a clinical intervention

**DOI:** 10.1007/s11306-026-02490-w

**Published:** 2026-07-27

**Authors:** Rachel M. Wood, Laura J. Corbin, Jane M. Blazeby, Chris A. Rogers, Nicholas J. Timpson, Daniel J. Lawson

**Affiliations:** 1 School of Mathematics, University of Bristol, Woodland Road, Bristol, BS8 1UG UK; 2 Population Health Sciences, University of Bristol, First Floor, Augustine’s Courtyard, Orchard Lane, Bristol, BS1 5DS UK; 3 Medical Research Council Integrative Epidemiology Unit, University of Bristol, First Floor, Augustine’s Courtyard, Orchard Lane, Bristol, BS1 5DS UK; 4 Population Health Sciences and Bristol Biomedical Research Centre, University of Bristol, 5 Tyndall Avenue, Bristol, BS8 1UD UK; 5 University Hospitals Bristol and Weston NHS Foundation Trust, Marlborough Street, Bristol, BS1 3NU UK; 6 Bristol Medical School, University of Bristol, First Floor, 5 Tyndall Avenue, Bristol, BS8 1UD UK

**Keywords:** CLARITY, Metabolomics, NMR, Multivariate, Network

## Abstract

**Introduction:**

High throughput metabolomic assays offer a huge opportunity to quantify the cellular processes underlying disease and intervention pathways. However, the multi-dimensional inter-relatedness between these processes coupled with the complex noisy measurement environment create a need for generation of new methods that move beyond simple pairwise associations.

**Objectives:**

To evaluate the utility of a simple, multivariate, relational comparison method called CLARITY in the context of metabolomics data.

**Methods:**

Nuclear magnetic resonance (NMR)-derived metabolomics data collected from the same individuals (*N* = 125) before and after a weight management intervention in By-Band-Sleeve (BBS), a clinical trial of metabolic and bariatric surgery, were used. First a traditional (univariate) linear mixed model approach was taken to identify metabolites that were changed post-intervention. The CLARITY method was then used to generate a covariance-based relational anomaly score with a view to increasing classification performance of the underlying cause of changes to the levels of and covariances between metabolites.

**Results:**

CLARITY enabled further characterisation of metabolites identified in univariate linear regression analyses, differentiating those that exhibited covariance changes from those with mean changes only. An additional cluster of metabolites were identified as undergoing a relational change that would not have been detected using traditional methods.

**Conclusions:**

Gathering insights about biological pathways from large-scale metabolomics data has the potential to inform the future design of modelling and laboratory experiments aimed at capturing the underlying biological processes relevant to disease.

**Supplementary Information:**

The online version contains supplementary material available at https://doi.org/10.1007/s11306-026-02490-w.

## Introduction

As the end products of cellular regulatory processes, metabolite levels reflect the response of biological systems to genetic or environmental changes. Capturing cellular responses to endogenous and exogenous perturbations is valuable for the prediction, prognosis, diagnosis and aetiological dissection of disease. With rapid advances in high-throughput technology and bioinformatics now enabling absolute quantification of hundreds (or relative quantification of thousands) of metabolites from a single biological sample, the last decade has seen major growth in the use of metabolomics as a research tool in epidemiology. However, substantial challenges exist in the translation of this research into public health benefit, an issue that has been highlighted by those working directly in the field (Letertre et al., 2021; Li et al., 2024; Pinu et al., 2019).

Current technologies typically generate (semi-)quantitative data on hundreds to thousands of metabolites per sample analysed and this has prompted a shift from measuring one or a small number of ‘target’ metabolites (whose properties may be relatively well understood), to measuring snapshots of the entire metabolome. The high throughput, omic-scale, approach to metabolite analysis has led to the use of standard association testing in large-scale epidemiological studies - ‘metabolome-wide association studies’ (MWAS). This approach, by which an experimental factor is tested for metabolite association against a hypothesis of ‘no effect’ (Pernet 2017) in a univariate testing framework, has as its output a list of single ‘associated’ metabolites determined by the presence of sufficient evidence to justifiably be more than a chance event alone. Such metabolomic profiling has been used to identify biomarkers associated with a range of diseases and to characterise metabolic risk factors (Rattray et al., 2018; Würtz et al., 2017), but arguably has not fully exploited the analytical opportunities available in this field.

Univariate analyses disregard interrelationships between levels of different metabolites that may co-occur either due to biochemical pathways or because of shared genetic and/or environmental influences. This failure has been identified as a major limitation given the system-wide view provided by metabolomics data (Jansen et al., 2012). Furthermore, the coexistence of high throughput molecular measures of differing type and derived from the same biosamples, is presenting an opportunity to consider multi-omic causal complexes which are only maximised when considered in multivariate analyses. Whilst existing multivariate approaches – typically ‘factor analysis’ methods, e.g. principal component analysis (PCA) – go some way to addressing this problem, these also fail to explicitly uncover changes in the relationships between metabolites under different conditions. Although not routinely implemented within the field of epidemiology, correlation network analysis has the potential to reveal differential connectivity patterns between features. To date, this approach has been applied to a limited extent within metabolomics and whilst these studies have found evidence for potentially meaningful differences in connectivity (Afzal et al., 2020; Saccenti et al., 2015; Vignoli et al., 2018, 2020), the limitations of the correlation network approach are well established (Masuda et al., 2025).

Metabolite-metabolite networks are typically underpinned by correlation matrices that capture the pairwise similarity between metabolites. However, it is not always clear how best to translate the correlation matrix into a network, with optimal approaches being dependent on the specific application. A key question is which relations to include in the network; the most common approach is one of thresholding such that only those pairwise relationships whose correlation coefficient exceeds some prespecified (and essentially arbitrary) threshold for relevance are represented in the network. However, there is no consensus regarding what an appropriate threshold for edge inclusion might be (and the choice is known to influence results). Challenges also exist in how to deal with negative correlations, edges with low correlations increasing uncertainty in the network, false positives resulting from indirect associations, varying edge density across individuals (or groups) and small sample sizes (Masuda et al., 2025). Methods continue to be developed to address these complexities including, proportional thresholding (to address varying edge density) and sparsity constraint, e.g. Gaussian graphical models (Iqbal et al., 2018), however, an alternative approach is to avoid transforming the correlation matrix data into a network all together (Masuda et al., 2025). Here we explore the application of an approach designed to compare heterogeneous data using (dis)similarity in the context of metabolomics data.

When two datasets describe the same entities (typically samples or individuals), many scientific questions can be phrased around whether the (dis)similarities between entities are conserved across strata of interest (be it time, study, treatment, group). Here we implement CLARITY, a method that quantifies consistency across datasets, identifies where inconsistencies arise and aids in their interpretation (Lawson et al., 2021). Applications of this software to date have been limited to exploratory analysis for anomaly detection, and only synthetic data in a bioinformatics context. Here we evaluate what is detected when the shared entities are common omics – in this case a shared set of nuclear magnetic resonance (NMR)-derived metabolites, with the two datasets representing different sampling timepoints for the same individuals. This “paired” data allows an integration with regression methods, informing on the type of anomalies to be expected in an unpaired context by CLARITY. By matching simulated data to a real dataset we can characterise the underlying network in terms of “direct” and/or “indirect” or inter-relational changes in metabolites that occur following a clinical intervention.

## Methods

### Study overview

**Fig. 1:**
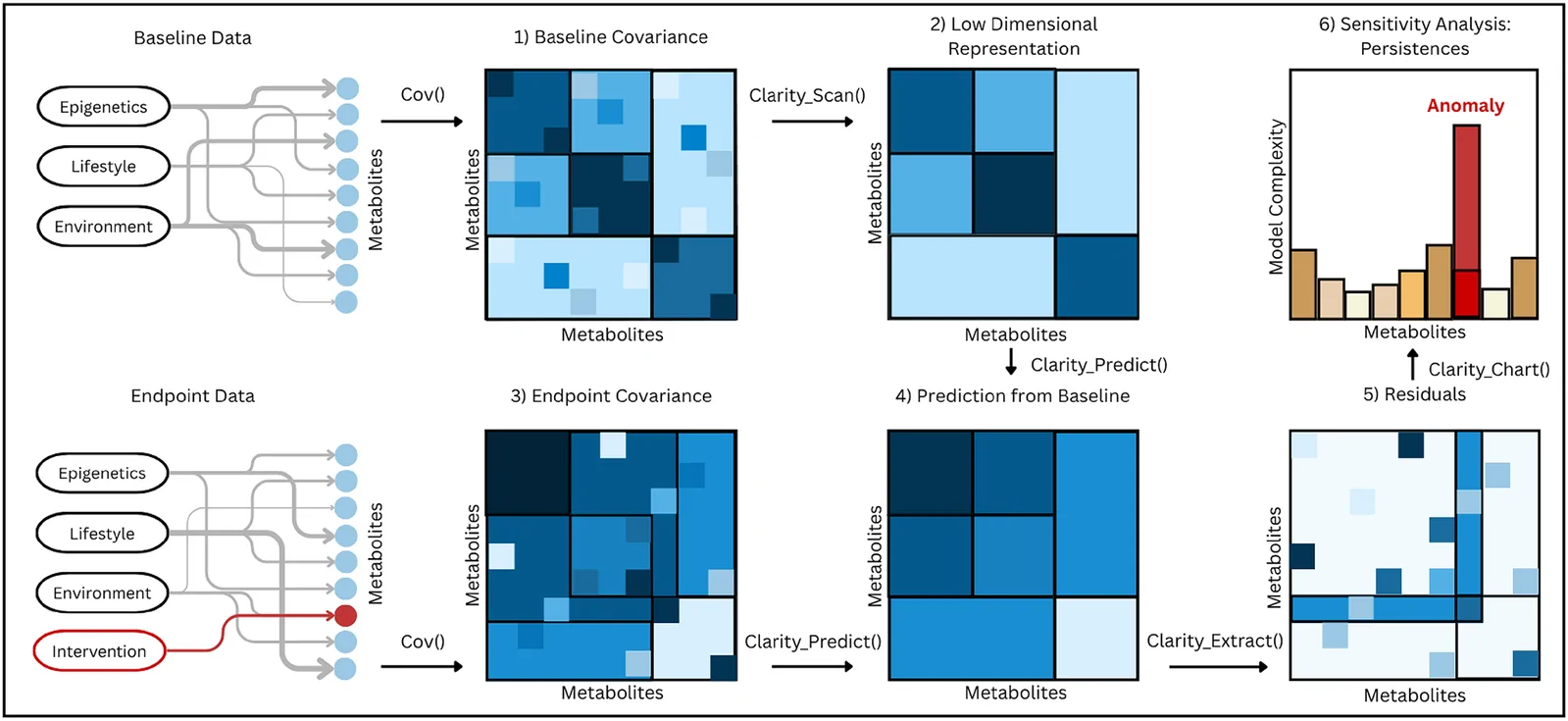
Conceptualisation of metabolite model and corresponding schematic of CLARITY. We consider two instances of the data (corresponding to before and after an intervention). Covariates – in our example epigenetics, lifestyle and environmental factors, affect the metabolites at strengths represented by edge widths. A cluster of covarying metabolites can be thought of as capturing part of a “metabolic pathway”. In the endpoint, a post-treatment pathway variable is added which affects a subset of metabolites, which we are aiming to identify with our methods. Edge weights and confounders correlate but differ from baseline to endpoint. Our simulated data follows this cartoon representation

Metabolite data are complex and a minimal model risks oversimplification. NMR data in particular is characterised by relatively high redundancy and whilst this can lead to over conservative corrections for multiple testing in univariate frameworks it means that these data are well-suited to data compression techniques. The same metabolite observed at different times will systematically differ in a way that is not explainable using observed confounders, whilst groups of metabolites will be correlated due to both observed and unobserved confounders. The signal of an intervention effect sits on top of this complex structure and is also expected to be complex in the sense of affecting multiple metabolites. An illustration of the structure of this generative model is shown in Fig. 1 (Left). Detecting an anomaly after intervention is therefore not as simple as identifying a statistically robust signal of change. To improve classification of anomalies into their root causes, we apply CLARITY (Fig. 1, Right), a non-parametric multivariate comparison method designed to identify correlations that were not already found in the pre-intervention state. For example, doubling all metabolite levels would have no effect on test statistics derived from CLARITY; neither would changing metabolite levels in a major correlated baseline cluster as might be introduced by unobserved confounding. CLARITY is designed to detect endpoint correlations that were not in the baseline, such as would be generated by activating a new pathway as described in our model, without making explicit modelling assumptions. Here, CLARITY is used alongside a traditional linear mixed model (LMM) approach for identifying differences in means with a view to assessing the potential value added by the CLARITY analysis.

### Experimental data

Metabolomics data derived from samples collected during the By-Band-Sleeve (BBS) clinical trial were used. BBS is a pragmatic randomised controlled trial (RCT) which took place in the UK (registration number: ISRCTN00786323). The trial investigated the clinical- and cost-effectiveness of three types of metabolic and bariatric surgery: the Roux-en-Y gastric bypass (“Bypass”), laparoscopic adjustable gastric band (“Band”) and the sleeve gastrectomy (“Sleeve”). A full description of the trial can be found in published works; specifically, the trial protocol (Rogers et al., 2014), a description of the baseline data (By-Band-Sleeve Collaborative Group, 2023) and the primary results (By-Band-Sleeve Collaborative Group, 2025). In this study, serum samples that were collected at baseline (prior to surgery) and at 36-months after randomization were used to generate metabolomics data. The median (interquartile range) time between baseline (randomization) and surgery was 2.7 (1.9, 4.5) months, and between surgery and 36-month follow-up was 33.4 (31.7, 34.8) months. Details of the sample collection and processing procedures have been previously described (Smith et al. 2025). Included here are data from a pilot study involving a subset of 125 patients who were recruited at a single hospital site and had serum samples available for analysis from both baseline and 36-months post-randomisation by June 2021. The 250 samples were sent for NMR metabolomics analysis by Nightingale Health (Helsinki, Finland).

A high-throughput ^1^H-NMR metabolomics platform was used to quantify 250 metabolic biomarkers, 160 in absolute levels and 90 derived (ratio/percentage/total) measures (full details in Online Resource 1) and data were received in October 2022. The R package *metaboprep* (Hughes et al., 2022) was used to perform pre-processing and quality control (QC) of the NMR data. Any samples and features with extreme missingness (> 80%) were excluded before recalculating sample and feature missingness. Then samples with more than 20% missingness and features with more than 20% missingness were removed from the dataset. PCA analysis was also performed and any samples that were more than five standard deviations (SDs) from the mean of the first and/or second principal components were filtered from the data set. No imputation of the data was performed. A summary of dataset QC can be found in Online Resource 2. After QC filtering, 245 samples and 250 metabolites remained in the dataset. Finally, data for individuals who did not have surgery before the 36-mth post-randomization sample collection timepoint, were excluded from analyses. This left data for 237 samples from 121 unique individuals. Metabolite data were restricted to the 160 absolute measures (i.e., derived measures were excluded). Because the metabolomics data are measured on widely variable scales, to give equal weight in the algorithm they were jointly mean centred and scaled to have SD 1 using the base R function *scale()*; this scaling procedure was performed on the full sample set (i.e., on baseline and endpoint samples together).

### Simulated metabolite data

To aid interpretation of the results from the analysis of experimental data, we introduce a simulation study that captures the most important features – following the framework described in Online Resource 1 (‘*Simulation Pipeline*’) - and for which the ground truth is known. The simulation was designed to represent a typical NMR-derived metabolite dataset with similar properties to our experimental data set. In this we simulated metabolites to represent shared causal pathways from covariates, whereby metabolites belong to clusters which are jointly activated by those covariates in a hierarchical model. Specifically, each covariate activates two clusters, each differently, and each metabolite differently within a cluster. Anomalies were then planted in the ‘endpoint’ observation that affect either a whole cluster or only half of a cluster.

Data was generated for *n* subjects and $\:M\:$metabolites, using an idealised notion of “pathways” - here defined as a set of metabolites that covary together. The metabolites are simulated according to a linear model, with inputs being made up of observed confounders (* C*) which are given as covariates in the LMM and unobserved confounders (* U*) which are hidden from the LMM. We wish to detect an intervention “pathway” $\:{C}^{*}$ for which coefficients are non-zero only in the endpoint. Then metabolite $\:j\:$for subject $\:i\:$ at time t is given by:

$$\:{M}_{ij}^{\left(t\right)}=\:{C}_{i}{\beta\:}_{{C}_{ij}}^{\left(t\right)}+{U}_{i}{\beta\:}_{{U}_{ij}}^{\left(t\right)}\:+{C}_{i}^{*}{\beta\:}_{ij}^{*\left(t\right)}+{E}_{ij}^{\left(t\right)}$$

where the error matrices $\:{E}^{\left(1\right)},\:{E}^{\left(2\right)}$ are of size $\:n\:\times\:M\:$and generated independently according to a standard normal distribution. The important component of the model is the correlated effect sizes $\:\beta\:=\left\{{\beta\:}_{{C}_{j}}^{\left(t\right)},{\beta\:}_{{U}_{j}}^{\left(t\right)}\right\}$ and the intervention pathway effects $\:{\beta\:}_{j}^{*\left(t\right)}$.

Each metabolite is assigned to one of $\:c\:$evenly sized clusters, which informs how the $\:\beta\:$s are chosen to create a covariance structure representing critical features of clinical data. The metabolite data from both time points can be thought of as being generated by a multi-level hierarchical model which defines the covariance of the effect sizes $\:\beta\:$.

Specifically, let $\:{{\Sigma\:}}^{l}$ define the covariance of all $\:\beta\:$s within a time point for covariate $\:l\:$, for which we choose two clusters at random to be “activated”. $\:{{\Sigma\:}}_{j{j}^{{\prime\:}}}^{l}={v}_{0}$ if $\:j\:$ or $\:{j}^{{\prime\:}}$ are outside of the activated clusters, $\:{v}_{1}$ if they are in different activated clusters, $\:{v}_{2}$ for the same activated cluster and $\:{v}_{3}$ if $\:j={j}^{{\prime\:}}$ in an activated cluster. By choosing $\:{v}_{0}<\:{v}_{1}<{v}_{2}<{v}_{3}$, the covariance is positive definite.

The joint effect sizes for each covariate $\:l\:$ across both time points are generated from a joint normal distribution. The mean for this distribution is set to 0 and the full covariance matrix $\:{\varSigma\:}^{\mathrm{l}}$ is:

$$\:{\varSigma\:}^{l}=\:\left[{\:}_{{\rho\:}_{t}^{2}{{\Sigma\:}}^{l}\:\:\:{{\Sigma\:}}^{l}}^{{{\Sigma\:}}^{l}\:\:\:\:\:\:\:{\rho\:}_{t}^{2}{{\Sigma\:}}^{l}}\:\right]$$

where $\:{\rho\:}_{t}$ is the correlation of effect sizes across time.

Finally, the intervention pathway $\:{\beta\:}_{j}^{*\left(baseline\right)}=0$ for each metabolite $\:j\:$ whilst a subset of metabolites $\:\left\{{M}_{j}\right\}$ has $\:{\beta\:}_{j}^{*\left(endpoint\right)}\ne\:0$ within two randomly selected clusters. In one cluster $\:{c}_{1}$, the effects $\:{\beta\:}_{j}^{*\left(endpoint\right)}\ne\:0$ for the whole cluster, which we interpret as strengthening an existing pathway. In the other selected cluster, a random half of metabolites $\:j\:$ have $\:{\beta\:}_{j}^{*\left(endpoint\right)}\ne\:0$, which we interpret as a new pathway. We call this half of the cluster $\:{c}_{2}$ and further denote the remaining half of the cluster $\:{c}_{3}$, which has not experienced mean shift, but a change of relationship with similar metabolites. In both $\:{c}_{1}$ and $\:{c}_{2}$ there is a direct “first-order” change, whereas $\:{c}_{3}$ can be seen as “second-order” because it experiences no direct change, but its covariance is changed through its relationship with other metabolites. The vector $\:{\beta\:}_{j}^{*\left(endpoint\right)}\sim N\left(m,{{\Sigma\:}}^{*}\right)$ consists of a mean change * m* (which can be zero) and a covariance increase parameterised as above.

Full details of how to generate $\:{\beta\:}_{C},\:{\beta\:}_{U},\:{\beta\:}^{*}$ and $\:{C}^{*}$ are provided in the Online Resource 1.

### Statistical analysis

#### Linear mixed model to detect mean differences

A linear mixed model (LMM) was implemented using the *lmer()* function from the *lmerTest* R package (Kuznetsova et al., 2017) to identify metabolite levels whose mean differed between the two time points represented in the data.

The following formula was used:

$$\mathrm{metabolite} \sim \mathrm{cov}_{1} + \cdots + \mathrm{cov}_{k} + \mathrm{timepoint} + \left( {1|\mathrm{studyID}} \right)$$

where for the experimental data, covariates were *storage.time + age + sex*, where *storage.time* is the estimated length of time in months between sample collection and aliquoting for metabolomics analysis, *age* is participant age at baseline in years, *sex* is a binary variable (self-reported sex, male/female), *‘studyID’* is the identifier for each participant, fitted as a random effect to account for the repeated measures, and ‘*timepoint*’ is the independent (or predictor) variable. For simulated data *k = 4* and cov_1_, …, cov_k_ correspond to the C matrix described in the previous section as continuous normally distributed random variables. Model betas for ‘*timepoint*’ represent the estimated difference in metabolite abundance (in SD units) at the second time point (post-treatment) as compared to the first (pre-treatment). Benjamini-Hochberg (BH) corrected p-value (Benjamini & Hochberg, 1995) was calculated using the “BH” method in the *p.adjust()* function from the ‘stats’ R package (R Core Team, 2025).

#### CLARITY analysis to detect structural differences

CLARITY is a method to detect differences in structure – meaning components analogous to clusters or shared variance components - between two datasets. If we knew the causal pathways, we would regress these out to identify the changes, but these are very difficult to learn. CLARITY solves this problem by building a representation of the similarity between metabolites at baseline, which is used to predict the similarity at endpoint. In this way, change in the relative importance of metabolite groupings that are present in the baseline will not be identified, but metabolite groupings present in the second dataset alone are reported as anomalies. CLARITY uses a non-parametric approach in which increasingly complex representations of the first dataset are used to predict the second. An “anomaly” is therefore defined as a structure present in the second dataset and not in the first, that “persists” across a wide range of model complexities. In the current application, we reframe the CLARITY approach such that the shared entities are common omics – in this case a set of measured metabolites, rather than individuals or samples. The same metabolites have been measured at two different sampling timepoints. A full description of the functions from the R CLARITY package (Lawson et al., 2021) that were applied to both the simulated and the experimental data can be found in Online Resource 1 (‘*Application of CLARITY to Metabolomics Data’*). A schematic of the CLARITY approach is shown in Fig. 1.

The derived test statistic from CLARITY is named “persistence” because the set of metabolites *j* that have large residuals remains highly consistent for a wide range of model complexities *k* by construction. A cross-validation procedure for computing p-values for each metabolite was implemented in *Clarity_Compare()* (described in Online Resource 1). For the simulated data, the p-values were taken, with *k* to be the number of simulated clusters. In the analysis of the experimental data (where *k* is unknown), persistence values were computed for all *k*. As claimed from the name, it is observed that an almost identical set of anomalous metabolites “persist” i.e. have extreme test-statistic values, for a wide range of *k* from 10 to 40. We therefore discuss and interpret only anomalies that persisted over this range of *k*, whilst corresponding test statistics (i.e., p-values) are reported for rank *k = 25*.

#### Technical choices within the CLARITY pipeline

CLARITY compares the similarity between metabolites at baseline to the endpoint. The selection of similarity measure is an important implicit modelling choice that should be carefully considered. Firstly, metabolites abundance varies by orders of magnitude and therefore scaling the variance is essential. Secondly, the mean level of metabolites should not be informative to changes and therefore mean centering is appropriate. These transforms are applied to the joint dataset over baseline and endpoint; to transform separately will lose power but not validity. We choose to work with covariances because these are interpretable, but any pairwise comparison such as cosine distance, and other scalings such as log-transforms are possible. A sensitivity analysis without mean centering is shown in Online Resource 1 (‘*Pre-processing of inputs*’).

The choice of similarity affects power but not validity because of the careful treatment of the test statistic. CLARITY constructs a conservative test statistic through a test/train resampling procedure to compare distributions within the baseline to that in the endpoint, for which the lower quantiles remain bounded as the number of subjects grows under the null (see *Notes on CLARITY test statistics* in Online Resource 1). Given this sample-scaled conservativeness property and the compute costs associated with generating small p-values by bootstrap, BH correction is not usually recommended because the family-wise error rate with no positive cases is < 0.1% at a nominal value of 5%.

Further details and deeper discussion of other choices are given in (Lawson et al., 2021).

## Results

### Experimental data

Of the 160 NMR metabolites tested in the LMM, 30 (19%) were altered by the intervention (BH-corrected *p* < 0.05) (Supplementary Table 1 in Online Resource 3). The strongest association was seen for triglycerides in large HDL (beta = 0.78 SD, 95% CI: 0.44, 1.12, BH-adjusted *p* = 0.002) with three other metabolites having BH-adjusted *p* = 0.003 (average diameter of HDL particles (beta = 0.69 SD, 95% CI: 0.36, 1.02), valine (beta = −0.75 SD, 95% CI: −1.11, −0.39) and isoleucine (beta = −0.71 SD, 95% CI: −1.06, −0.37)). There were relatively more metabolites from the ‘branched chain amino acid (BCAA)’ class in the LMM-associated subset as compared to all metabolites tested (13.3% compared to 5.6%) (Supplementary Fig. 1 in Online Resource 4).

**Fig. 2:**
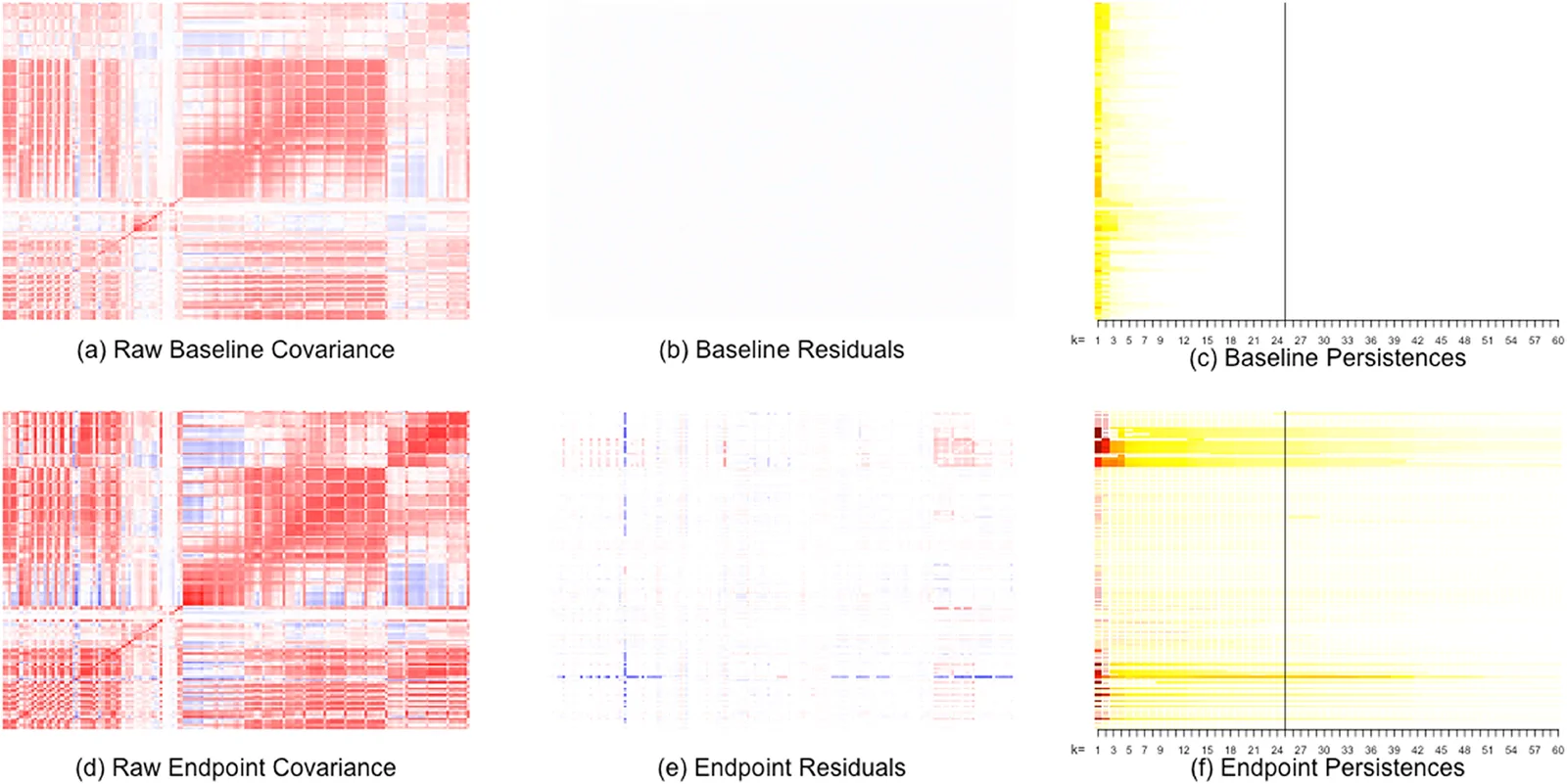
CLARITY plots for experimental data. A representation of the clinical data as it moves through the CLARITY pipeline. The first **a**–**c** and second **d**–**f** rows correspond to the baseline and endpoint data respectively. The first column (plots **a**, **d**) shows the raw covariance matrices. The second column (plots **b**, **e**) shows the residuals of the CLARITY model at k = 25 on the same scale, “fading” (making transparent) non-significant residuals. The third column (plots **c**, **f**) shows the persistences from k = 1, …, 60 on the same scale, with a black line indicating the persistences for k = 25 and “fading” p-values > 0.05. Scale: red: large and positive, decreasing through yellow and white=zero, to blue: large and negative

**Fig. 3:**
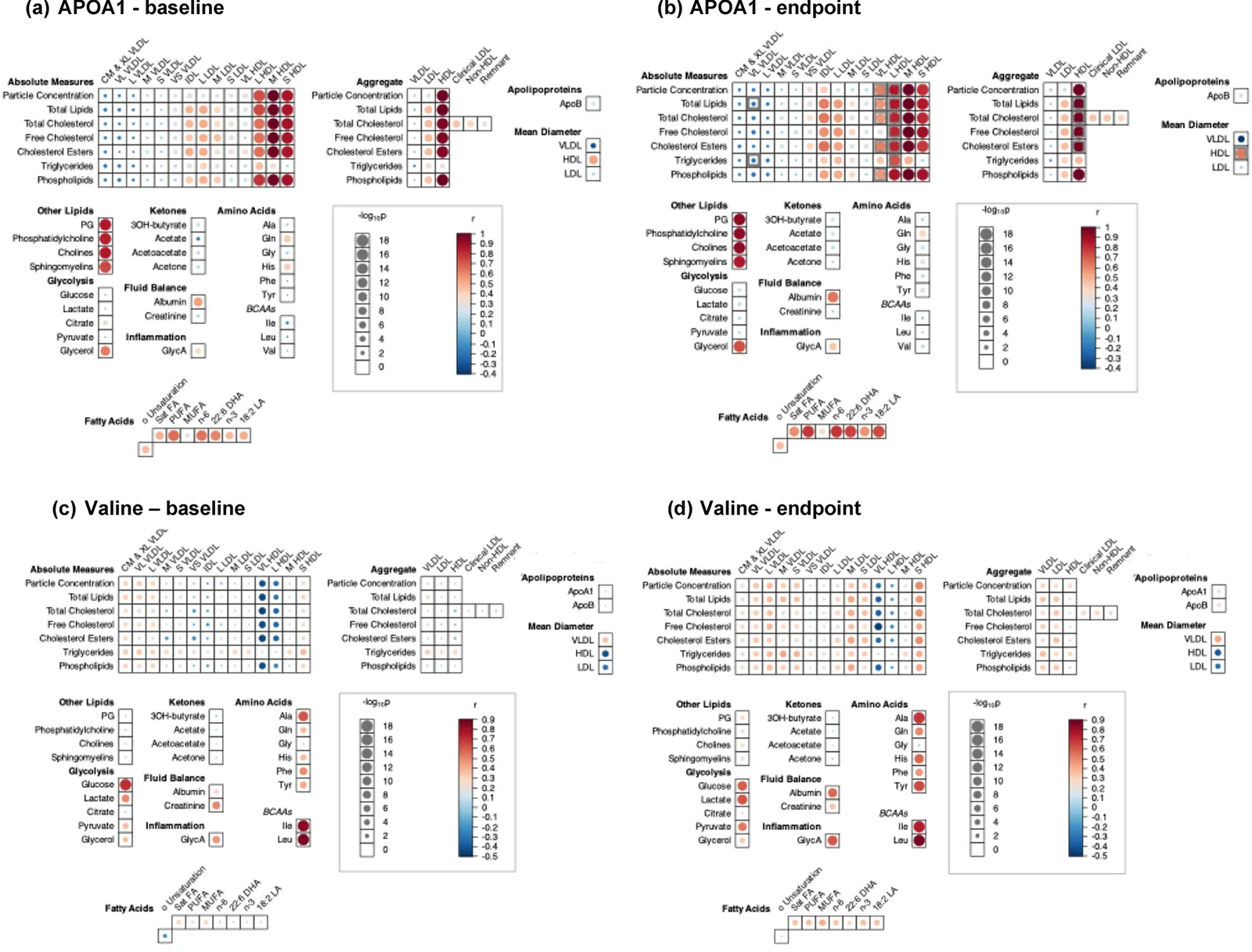
Correlation heat maps showing the relationship between APOA1 **a**, **b** and valine **c**, **d** and all other measured metabolites firstly at baseline **a**, **c** and then at endpoint **b**, **d**. APOA1 showed persistence in the CLARITY analysis and grey squares highlight which APOA1-metabolite relationships changed (*p* < 0.05). Valine did not show persistence in the CLARITY analysis and no meaningful differences in valine-metabolite relationships were identified. Plots produced using a modified version of the R package ‘bubbleHeatmap’(Boxall, 2023). r = Pearson correlation; -log_10_p = minus log_10_ of p-value from test of Pearson’s correlation

In the CLARITY analysis, 28 metabolites (18%) had an empirically derived persistence *p* < 0.05 (with k = 25) suggesting that they could not be well-predicted at the 36-month timepoint based on baseline (reference) data structure, even in models with a relatively high degree of complexity (Fig. 2). The five metabolites with the greatest persistence were Concentration of large HDL particles, Total cholesterol in large HDL, Cholesterol esters in large HDL, Total lipids in large HDL, and Phospholipids in large HDL (Supplementary Table 1). There were relatively more metabolites from the ‘other lipids’ class in the subset with persistence p < 0.05 as compared to all metabolites tested (10.7% compared to 2.5%) (Supplementary Fig. 1). The distribution of pairwise correlations across metabolites varied with those metabolites from the amino acid class typically showing a normal distribution centred on zero and those from the lipid classes frequently exhibiting multi-modal distributions indicating moderate to strong positive and negative correlations with many other metabolites (Online Resources 5 and 6). Visualising the between metabolite correlations at the two timepoints helps to elucidate the changes that have occurred in the case of metabolites that returned high persistence values (Fig. 3a, b), whereas for metabolites that did not demonstrate persistence there is no substantial change in correlation structure (Fig. 3c, d).

To check for simple explanations for our results, we computed the total correlation (sum of absolute pairwise correlations, *r*, values) for each metabolite first at baseline and then again at endpoint. Examining the difference, the metabolites with persistence *p* < 0.05 exhibited differences in total correlation across the full range (decrease, increase and no difference) (Supplementary Fig. 2 in Online Resource 4), i.e. our findings cannot be explained by changes in the metabolite’s total correlation from baseline to endpoint. It is critical to consider the persistence value in the context of an empirically derived test statistic. Whilst a small number of metabolites returned particularly high persistence values, these were found not to be statistically meaningful when put in the context of the empirically derived p-value (Supplementary Fig. 3 in Online Resource 4, top panel), likely because they were simply very variable between time points. There was additionally no strong relationship between total correlation at baseline and persistence (Supplementary Fig. 3, middle and bottom panel). From these sanity checks we conclude that the anomalies do indeed represent a change in the covariance between metabolites.

**Fig. 4:**
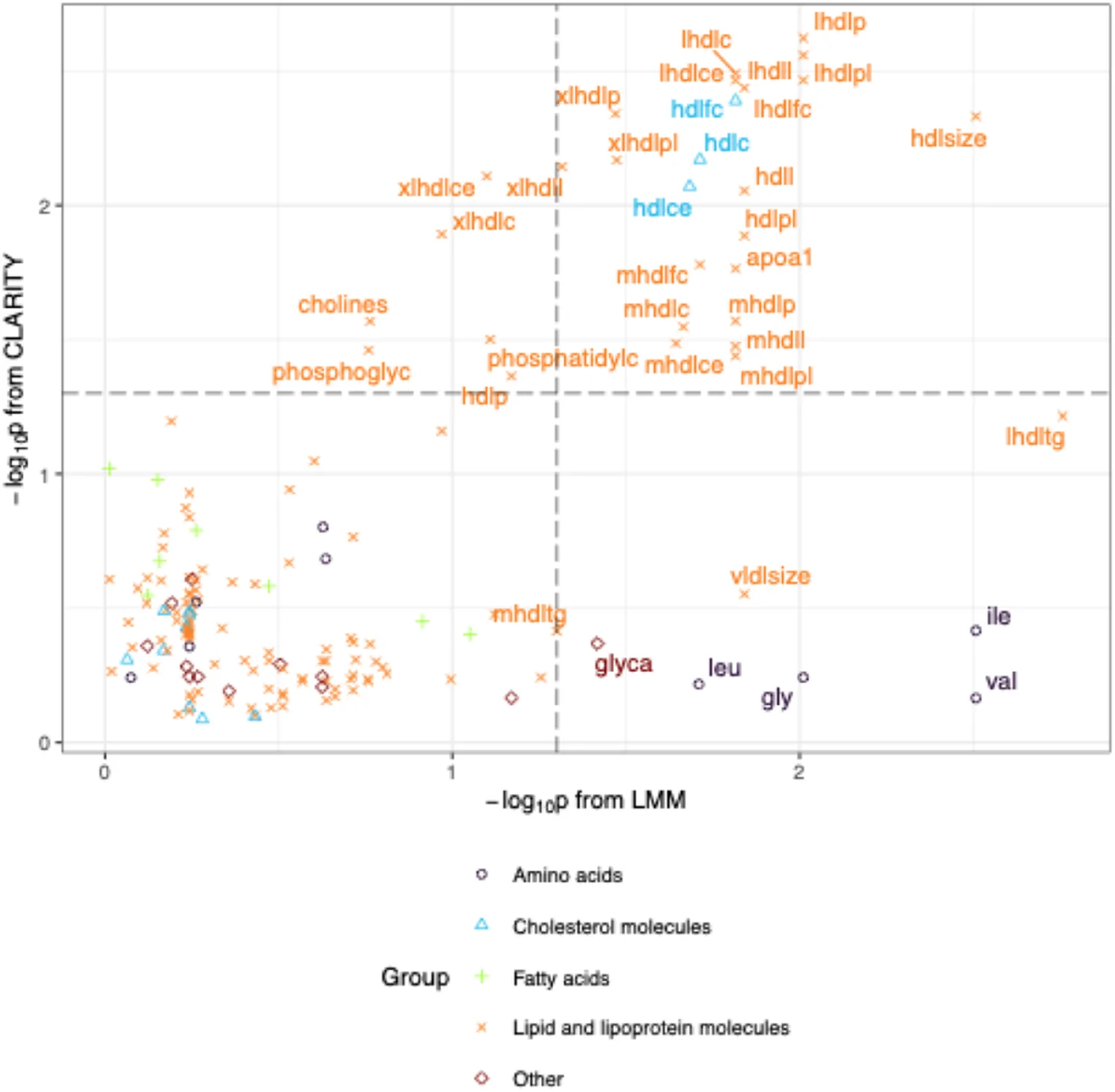
Association statistics from the two methods, linear mixed model (LMM) and CLARITY, applied to experimental data. Metabolites have been grouped according to class. Grey dashed lines have been included to indicate *p* = 0.05 (BH-adjusted for LMM). Metabolites with *p* < 0.05 in one or both analyses have been labelled (full names and classes provided in Supplementary Table 1). LMM = linear mixed model. For a more detailed exploration of the pre- and post-intervention metabolite relationships for APOA1 and valine (val), see Fig. 2

A comparison of the two approaches shows a moderate degree of overlap (Fig. 4). While some metabolites have an approximately proportional signal in the LMM and CLARITY analyses (based on respective *p*-values), others are only identified using one of the approaches. For example, the branched chain amino acids (BCAA) have a strong signal in the LMM analysis but do not show meaningful persistence in the CLARITY analysis. Extra-large HDL molecules predominate in the list of metabolites identified more strongly in the CLARITY analysis. And amongst those shown to differ in both approaches are several medium and large HDL molecules, as well as HDL particle size and APOA1 (Fig. 3a, b).

### Simulated data

We considered how each method performs in identifying multiple types of planted changes. The simulation framework described above was deployed with four observed and two unobserved covariates. There were seven clusters of metabolites with correlation across time points $\:\rho\:=0.8$, the minimum covariance was $\:{v}_{1}=0.0375$, the within-activated cluster covariance was $\:{v}_{3}=0.12$, and the between-activated cluster covariance $\:{v}_{2}=\left({v}_{1}+{v}_{3}\right)/2$. We explored the case with a large intervention pathway $\:{C}^{*}$ mean change of *m =* 0.5.

**Fig. 5:**
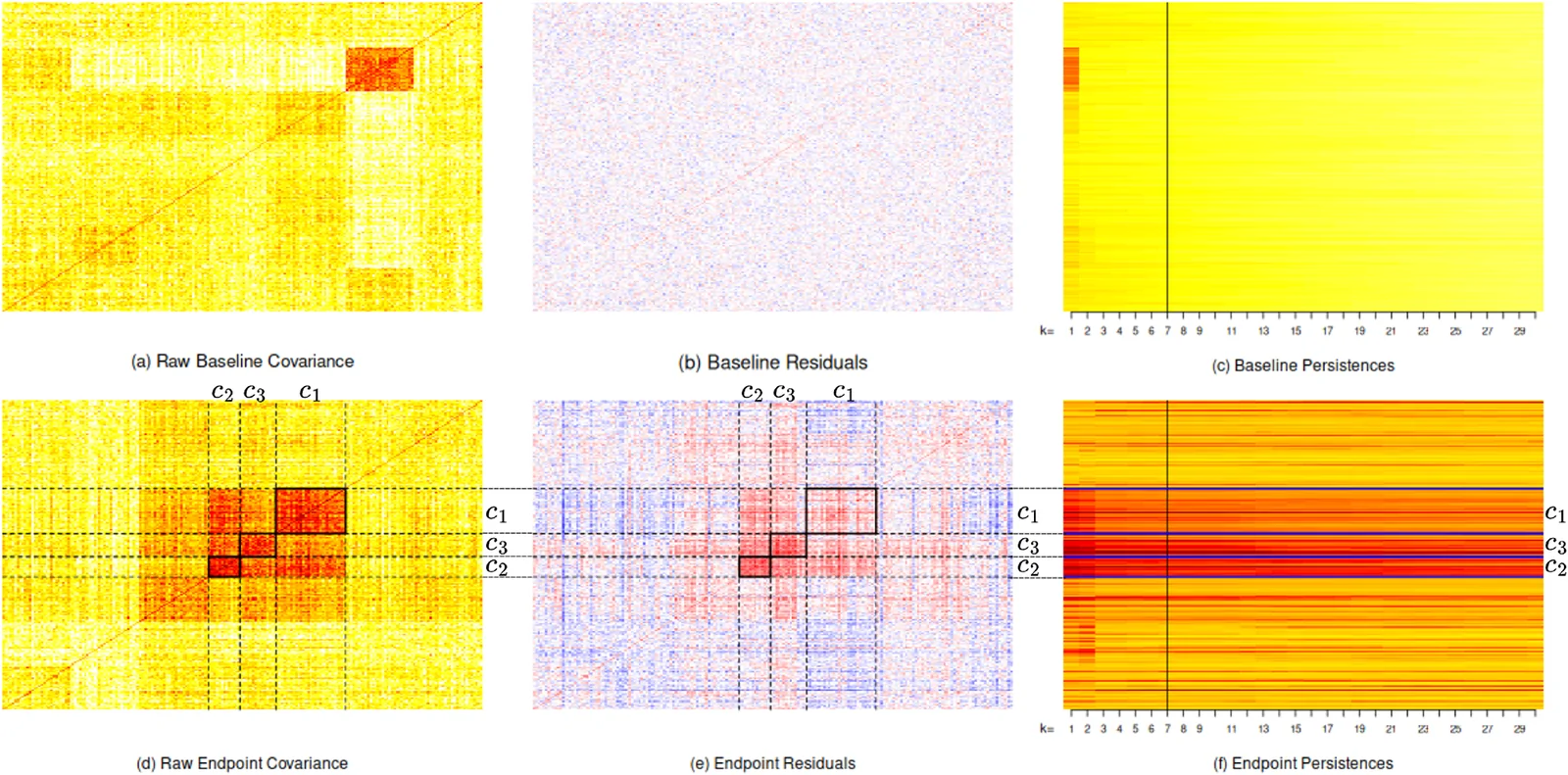
CLARITY plots for simulated data. A representation of the simulated data as it moves through the CLARITY pipeline. The first (**a**–**c**) and second (**d**–**f**) rows correspond to the baseline and endpoint data respectively. The first column (plots (**a**, **d**)) shows the raw covariance matrices. The second column (plots (**b**, **e**)) shows the residuals of the CLARITY model at k = 7. The third column (plots (**c**, **f**)) shows the persistences from k = 1, …, 30, with a black line showing the persistences for k = 7. In the endpoint plots, dashed lines and rectangles indicate the clusters that have a planted change. Scale: red: large and positive, decreasing through yellow and white=zero, to blue: large and negative

Figure 5 shows the covariance matrices and how well CLARITY predicts their structure when its complexity parameter *k* was set to seven, the number of simulated clusters. The residual matrix for the simulated baseline is simply noise, suggesting this representation is complex enough to capture the structure of the baseline data. In the endpoint data, there is still structure in the residual matrix; the persistence is highest for metabolites in clusters $\:{c}_{2}$ and $\:{c}_{3}$, with $\:{c}_{1}$ also showing smaller but still substantial persistences.

**Fig. 6:**
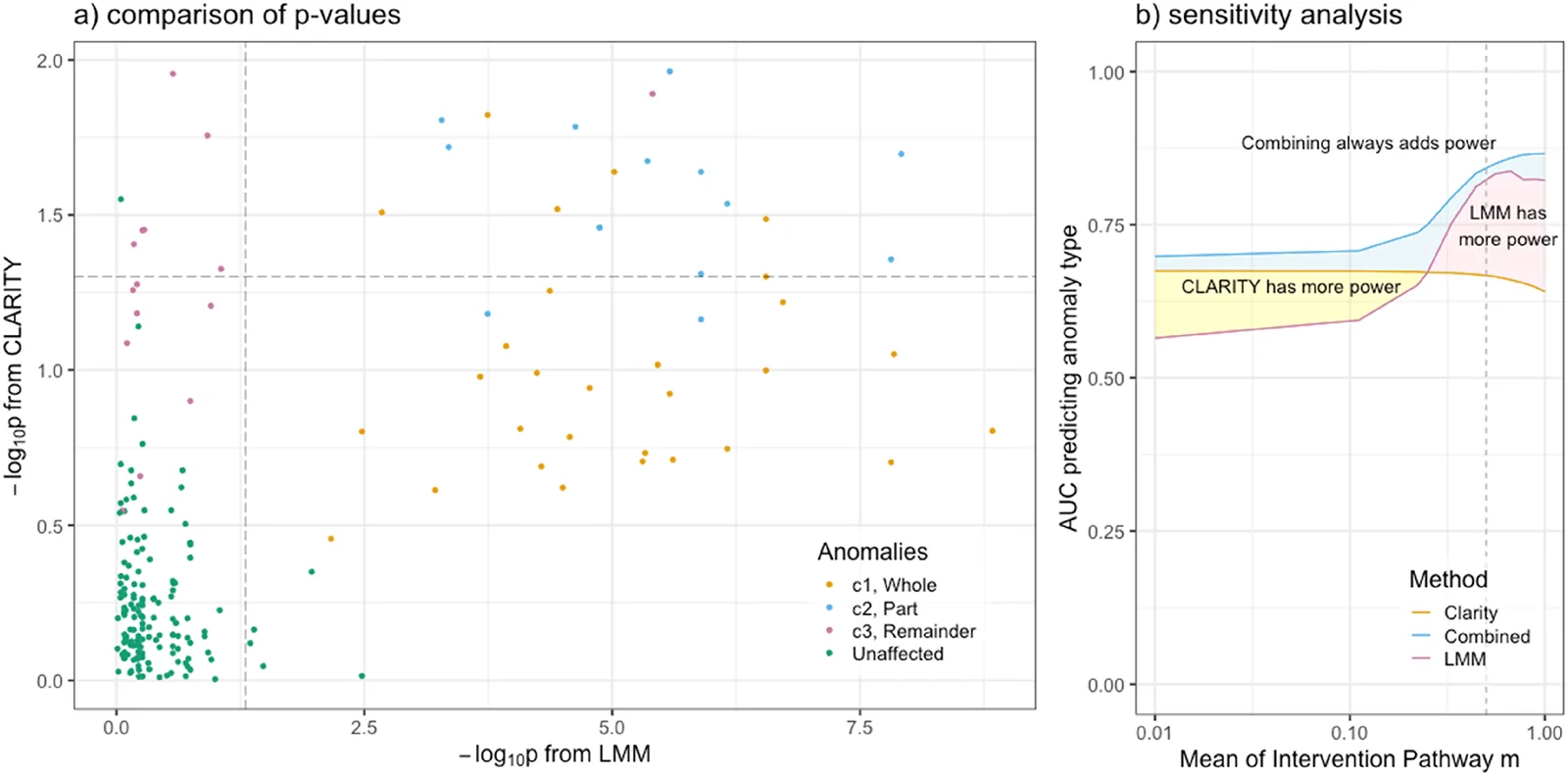
Linear mixed model (LMM) and CLARITY associations in simulated data. **a** Association statistics from the two methods, with metabolites coloured to reflect the type of change they experienced. Grey dashed lines have been included to indicate *p* = 0.05. LMM = linear mixed model. **b** Performance metric recovering the exact anomaly class type as measured by Area under the ROC curve (AUC) averaged over 500 simulations at each intervention mean m, using a test statistic of the CLARITY persistence, LMM p-value, or Combined, as a function of intervention mean $\:\mathrm{m}$. Grey dashed line at 0.5 shows the parameters from **a**

While metabolites do not fall into precisely defined groups, we can see that there are broad trends in where different classes fall in Fig. 6a. Metabolites in $\:{c}_{1}$and $\:{c}_{2}$, which have experienced a first order change (through the additive $\:{C}^{*}{\beta\:}^{*}$term), show a consistently stronger signal in the univariate analysis. In contrast, CLARITY rates the change in $\:{c}_{2}$ and $\:{c}_{3}$ to be stronger on average, as the structure of that block has changed from one uniform cluster to two clusters. Even though $\:{c}_{3}$ was not directly affected, it’s relationship with $\:{c}_{2}$ causes it to experience a second-order effect which can now be detected and classified. Results for a scenario with no mean change can be found in Online Resource 1 (‘*Comparison of p-values with no mean change*’). Figure 6b illustrates the complementarity of CLARITY and LMM information, in terms of the power to detect anomalies (as measured by the area under a receiver-operator curve, AUC). As the mean intervention decreases to 0, the LMM loses all power (AUC 0.5) and CLARITY always adds value to the LMM, even when mean interventions are large.

## Discussion

Metabolite profiles vary after an intervention for a variety of reasons. Many changes are technical as it is impossible to keep everything constant between observations of metabolites that vary due to many exogenous factors, including time of measurement, time since consumption of food, exercise, sleep and so on. These unobserved factors will be present at both time points but are expected to vary in relative impact, resulting in confounding for methods that look at before and after patterns of a single metabolite for each person.

The changes of interest in any given association analysis of longitudinal change are complex and involve a systematic change to the underlying (and largely unknown) metabolic pathways, therefore affecting many metabolites simultaneously. Information about these pathways is encoded in the co-occurrence patterns of metabolite profiles. We have shown that it is possible to identify sets of anomalous metabolites with new covariance structures after an intervention. Under our simulation model, this would be interpreted as the activation of a “metabolic pathway” in the sense of a group of metabolites responding together under this treatment condition. This was detected using CLARITY, a non-parametric analytical approach robust to noise and focused on evaluating the relative conservation of (dis)similarities.

We have shown using simulated data that if there were truly multiple metabolic pathways detectable as covarying metabolites and these varied in importance between baseline and endpoint, the CLARITY approach can correct for this and identify sets of metabolites involved in a pathway specific to the endpoint (here post-intervention) dataset – i.e. a change associated with the intervention. Conversely, traditional LMM approaches based on independent regressions identify a combination of covariance changes, mean changes, and confounding by technical changes. The metric used by CLARITY to quantify the degree of novelty (or unpredictability) of metabolites in the endpoint is a ‘persistence’ score. By considering the persistence score (and its test statistic) alongside results from traditional univariate analyses we can gain information regarding how (not just if) metabolites are different post-intervention.

In the CLARITY analysis of our experimental dataset, we observed meaningful persistence for 28 metabolites in total, six of which did not exhibit a strong effect in the LMM analysis including HDL particle concentration, cholines and phosphoglycerides. The sets of anomalous metabolites identified partition quite neatly into two classes, which we can interpret using the simulation analysis and by understanding the types of anomalies each method tries to identify. Firstly, metabolite anomalies identified by both methods (which CLARITY has more power to detect) are suspected of experiencing changes in both mean and covariance structure in the post-intervention state. Secondly, metabolite anomalies identified by the LMM only indicate a similar covariance structure at both timepoints. LMM approaches robustly detect mean changes which CLARITY is not designed to detect, but follow-up (experimental or otherwise) is needed to assess which type of change is most likely to lead to actionable insights. What is clear is the ability to annotate metabolites by whether they experience differences in mean and/or covariance compared to a baseline, is informative of the underlying biology and likely to be an important tool in the analysis of massively multivariate ‘omics data.

Our contribution is primarily methodological, and specific results should be seen as exploratory. We found that branched chain amino acids (BCAAs) (leucine, isoleucine, valine) showed a strong effect in the LMM but little persistence in CLARITY suggesting an effect of the intervention on mean levels via a pre-existing pathway, but no new relationship with other measured metabolites; indeed, this change in mean is supported by existing literature (Vaz et al., 2022). In general, and across both timepoints, the BCAAs exhibit a strong positive correlation with each other, a moderate positive correlation with other amino acids but a low correlation with most of the lipid metabolites that dominate our dataset (see Fig. 3c, d for valine as an example). To investigate whether the low overall correlation of the BCAAs with other measured metabolites was the reason they did not exhibit high persistence, we considered the relationship between total correlation and persistence. There was little indication that an overall low (or high) level of relatedness with other metabolites unduly influenced the CLARITY results. This suggests the cross-validation procedure used to generate empirical test statistics effectively accounts for such features in the data.

Metabolites identified with greater power in the CLARITY analysis were all lipids or lipid components with a predominance of very large HDL molecules. It is important to note that these metabolites fall into a continuum that includes metabolites that were identified by both methods, therefore showing both differences in mean and differences in their relationship with other metabolites. These included APOA1 (Fig. 3a, b), the principal protein component of HDL, as well as several medium and large HDL molecules. The identification of HDL molecules by CLARITY is in keeping with recent literature that points towards the relevance of HDL quality and functionality (determined by properties such as the composition of lipid and protein, and particle shape and number) and not just quantity to health (Cho, 2022). Investigating this group of molecules further is an important activity for future work.

CLARITY is an exploratory data analysis tool for approaching high-dimensional statistical comparisons and so is limited for drawing formal conclusions. Because it corrects for structure learned from the baseline dataset using arbitrary linear combinations in the test dataset, it has limited power to detect and quantify changes in factors already present. There are many situations we would expect these to change meaningfully despite the issues around confounding that are expected, particularly for metabolites. Methods to quantify this class of change would be welcomed, for example Unfolded Spectral Embedding (Gallagher et al., 2021) provides embeddings in which distances between multivariate embeddings (before vs. after) are meaningful, and which can be extended to similarities (Gallagher et al., 2024). Other limitations include a lack of theoretical guidance for how to pre-process the data, which is currently an implicit part of choosing a similarity measure. Here we present results based on centred and scaled input data and whilst scaling should not affect the inference, we present the results of the CLARITY pipeline on uncentered data in Online Resource 1.

There is a need to develop a wide range of tools that can detect different types of structural change in ‘omics data, which should ultimately lead to the ability to identify specific biological pathways impacted by an intervention. The CLARITY approach we have explored here identifies structural changes in metabolomic covariances that provides complementary information to traditional regression. It has the advantage of requiring no training data and because it uses covariances only, could be applied even if the two datasets were observed on different individuals. Next steps include comparison to a “healthy normal”, comparison of different treatments, and baseline validation where experimental work is available to probe the actual pathways activated.

## Supplementary Information

Below is the link to the electronic supplementary material.

Supplementary Material 1

Supplementary Material 2

Supplementary Material 3

Supplementary Material 4

Supplementary Material 5

Supplementary Material 6

## Data Availability

At the time these data were generated, participants were not asked for their permission to share data beyond the immediate project team. However, anonymised individual patient data from the By-Band-Sleeve trial will be made available upon request to the chief investigator (Jane Blazeby, [J.M.Blazeby@bristol.ac.uk](mailto: J.M.Blazeby@bristol.ac.uk)) for secondary research, conditional on assurance from the secondary researcher that the proposed use of the data is compliant with the Medical Research Council Policy on Data Sharing regarding scientific quality, ethical requirements, and value for money, and is compliant with the National Institute for Health and Care Research policy on data sharing. A minimum requirement with respect to scientific quality will be a publicly available prespecified protocol describing the purpose, methods, and analysis of the secondary research (e.g., a protocol for a Cochrane systematic review), approved by a UK Research Ethics Committee or other similar, approved ethics review body. Participant identifiers will not be passed on to any third party.

## Code availability

The software developed in this study is available via a GitHub repository at: https://github.com/lauracorbin/clarity_analysis_of_bbs_data.
