# Supplementary material for: Multivariate analysis of metabolomic data to identify biological pathways modified by a clinical intervention
Source: Metabolomics. 2026 Jul 27;22(4):134. doi: 10.1007/s11306-026-02490-w (PMC13407567; doi:10.1007/s11306-026-02490-w)
Supplement: Supplementary file 1 — Supplementary Material 1 [file 11306_2026_2490_MOESM1_ESM.pdf]

Online Resource 1:

Multivariate analysis of metabolomic data to identify biological pathways modified by a clinical intervention

*Rachel M. Wood[1], *Laura J. Corbin[2,3], Jane M. Blazeby[4,5], Chris A. Rogers[6], Nicholas J. Timpson[2,3], Daniel J. Lawson[1,3]

Supplementary Methods

Derivation of NMR data

A high-throughput ^{1}H-NMR metabolomics platform was used to quantify 250 metabolic biomarkers, 160 in absolute levels and 90 derived (ratio/percentage/total) measures. Details of experimental procedures have been described elsewhere (Ahola-Olli et al. 2019; Soininen et al. 2015). Data were received in October 2022. The biomarkers include detailed measures of cholesterol metabolism, fatty acid compositions and various low-molecular weight metabolites, such as amino acids, ketones and glycolysis metabolites. The 14 lipoprotein subclass sizes are defined as follows: extremely large VLDL with particle diameters from 75 nm upwards and a possible contribution of chylomicrons, five VLDL subclasses (average particle diameters of 64.0 nm, 53.6 nm, 44.5 nm, 36.8 nm, and 31.3 nm), IDL (28.6 nm), three LDL subclasses (25.5 nm, 23.0 nm, and 18.7 nm) and four HDL subclasses (14.3 nm, 12.1 nm, 10.9 nm, and 8.7 nm). The following lipid components within the lipoprotein subclasses are quantified: phospholipids (PL), triglycerides (TG), cholesterol (C), free cholesterol (FC), and cholesteryl esters (CE). The mean size for VLDL, LDL and HDL particles is calculated by weighting the corresponding subclass diameters with their particle concentrations (Soininen et al. 2015).

Simulation Pipeline

Input:

- n: sample size
- n_M: number of metabolites
- n_U: number of unobserved confounders
- n_C: number of observed confounders
- m: mean effect
- c : number of clusters
- σ_c: correlation of effects within a cluster
- σ_x: correlation of effects outside clusters
- ρ_t^2: shared covariance between effects across times

1. **Generate observed (C), unobserved (U) confounders and anomalous confounder**:
    1. Generate a mean for every confounder $l \in \{1, \dots, n_U + n_C\}$ according to

    $$\mu_l \sim U[0, 0.5]$$

    2. Set covariance matrix

    $$\Sigma^C_{kl} = \begin{cases} 1 & \text{if } k = l \\ 0.5 & \text{else} \end{cases}$$

    3. Obtain a sample of covariates of length $n_U + n_C$ for each subject i:

$$UC_i \sim_{iid} \mathcal{N}(\mu, \Sigma^C)$$

to get matrix $UC \in \mathbb{R}^{n \times (n_C + n_U)}$

4. Generate variable $C^* \in \mathbb{R}^n$ for each subject i which is only active in $t = 2$ for anomalous metabolites:

$$C_i^* \sim \mathcal{N}(m, 1)$$

2. **Divide metabolites into evenly sized clusters**, denote cluster memberships as $Z_j \in \{1, \ldots, c\}$ for each metabolite j.
3. **Create coefficient matrices** $\boldsymbol{\beta}^{(1)}, \boldsymbol{\beta}^{(2)} \in \mathbb{R}^{n_M \times (n_C + n_U)}$ in a way such that each confounder activates a two clusters (mimicking pathways). For every $l = 1, \ldots, (n_U + n_U)$, we consider the coefficients corresponding to the lth column of UC:
   1. Sample 2 clusters to be activated, say c_l^1, c_l^2
   2. Set matrix $\Sigma^l \in \mathbb{R}^{n_M \times n_M}$ to be the marginal covariance matrix for coefficients within a time point as follows:

$$\boldsymbol{\Sigma}_{jj'}^{(l)} = \begin{cases} \sigma_c & \text{if } Z_j = Z_{j'} \in \left\{c_l^1, c_l^2\right\} \\ \frac{(\sigma_c + \sigma_x)}{2} & \text{if } Z_j \neq Z_{j'}, \text{ and } Z_j, Z_{j'} \in \left\{c_l^1, c_l^2\right\} \\ \sigma_x & \text{else} \end{cases}$$

   i.e. σ_c within an activated clusters, $\frac{\sigma_c + \sigma_x}{2}$ across activated clusters and σ_c otherwise.

   3. Create covariance matrix to sample $t = 1, 2$ coefficients jointly:

$$\boldsymbol{\Sigma}^l = \begin{bmatrix} \boldsymbol{\Sigma}^l & \rho_t^2 \boldsymbol{\Sigma}^l \\ \rho_t^2 \boldsymbol{\Sigma}^l & \boldsymbol{\Sigma}^l \end{bmatrix}$$

   4. Draw l^{th} column of coefficient matrices as follows

$$\left[\beta_l^{(1)} \ \beta_l^{(2)}\right] \sim \mathcal{N}\left(\mathbf{0}, \boldsymbol{\Sigma}^l\right)$$

4. **Create anomalies**:
   1. Sample 2 clusters to be anomalies, say c_1^A, c_2^A and set anomaly status vector A to be 1 for all metabolites in c_1^A and half of the metabolites in c_2^A
   2. Obtain coefficients for C^* as $\boldsymbol{\beta}^* \in \mathbb{R}^{nX}$:

$$\boldsymbol{\beta}_i^* \sim \begin{cases} U[0.5, 1] & \text{if } A_i = 1 \\ 0 & \text{else} \end{cases}$$

5. **Produce metabolite data** X_1, X_2:
   1. Generate error matrices $E^{(1)}, E^{(2)} \in \mathbb{R}^{n \times nX}$ according to a standard normal for each subject i and metabolite j:

$$E_{ij}^{(t)} \sim \mathcal{N}(0, 1)$$

2. For each metabolite j at time t, get the vector $M_{ij} \in \mathbb{R}^n$ of observed metabolites:

$$M_{ij}^{(t)} = \begin{cases} C_i \boldsymbol{\beta}_{C_j}^{(t)} + U_i \boldsymbol{\beta}_{U_j}^{(t)} + E_{ij}^{(t)} + C_i^* \boldsymbol{\beta}_j^* & \text{if } i \in A, t = 2 \\ C_i \boldsymbol{\beta}_{C_j}^{(t)} + U_i \boldsymbol{\beta}_{U_j}^{(t)} + E_{ij}^{(t)} & \text{else} \end{cases}$$

Application of CLARITY to Metabolomics Data

All functions mentioned in this section form part of the R CLARITY package (Lawson et al. 2021). CLARITY was applied to the simulated and clinical datasets as follows. For this multivariate analysis, we are given feature matrices, denoted as D_1 (the reference data) and D_2 (the target data) each with L rows. From these, column-wise covariance matrices of the feature matrices of metabolites are taken, denoted as Y_1 and Y_2. Covariances were calculated using the *cov()* function in R using complete observations. Note that this removes the requirement that the datasets contain the same individuals. The first step is creating a rank k approximation of Y_1 with the *Clarity_Scan()* function:

$$\begin{aligned} Y_1 &= V_1^k \Sigma_1^k (V_1^k)^T + V' \Sigma V'^T \qquad n \\ &\approx V_1^k \Sigma_1^k (V_1^k)^T \end{aligned}$$

Y_1 and Y_2 are predicted using this rank k representation of the baseline Y_1, implemented in *Clarity_Predict()*, giving predictions $Y^{(t)}$ as:

$$Y^{(t)} = V X_t V^T$$

where $X^{(t)} = (V^T V)^{-1} V^T Y_t V (V^T V)^{(-1)}$ and $V = V_1^k$.

Clarity_Persistence() is then used to compute a test statistic, simply the squared row sums of residuals:

$$P_{jk} \ = || \ Y_{2,j} \ - \ Y_{2,j}^k \ ||_F^2$$

This test statistic is named “persistence” because the set of metabolites j that have large residuals remains highly consistent for a wide range of model complexities k by construction. Conceptually, the singular value decomposition (SVD) of the baseline data is learning structure (i.e. cluster-like objects) for small k, and noise (or structures that are not in the endpoint) as k grows. At very large k this allows over-fitting to the endpoint data, so an appropriate choice of k is from the wide region where anomalies persist.

A cross-validation procedure for computing p-values for each metabolite is implemented in *Clarity_Compare()* and described below.

Procedure:

For $r = 1, \ldots, R$:

1. Randomly split the features of D_1 to create D_0', D_1'.
2. Sample $L/2$ rows from D_2, call this D_2'.
3. Create covariance matrices Y_0, Y_1, Y_2 from D_0', D_1', D_2'.

4. Perform CLARITY on pairs (Y_0, Y_1) and (Y_0, Y_2) with rank k.
5. Compute test statistic $f(\text{persistences})$ for each row of Y_1 and Y_2:
    - The function *c_Scoresplit()* computes a Wilcoxon Rank Sum test statistic with a regularization of 1, specifically: let $x_{(r)}$ be the sorted replicate values from comparison of baseline to baseline, and $y_{(r)}$ be the sorted bootstrap values from comparison of baseline to endpoint. The test statistic $T(j,k)$ for metabolite j at complexity k is the regularised proportion of this distribution larger in the endpoint:

$$T(j,k) = \frac{1 + \sum_{r=1}^{R} \mathbb{I}\left(y_{(r)} > x_{(r)}\right)}{1 + R}$$

Pre-processing of inputs

When calculating covariance matrices, a choice needs to be made on whether to mean-center the raw data. In the main text, all figures show CLARITY on scaled and centered data to ensure as much consistency across metabolites as possible. The scaling is needed to ensure that metabolites with high concentrations do not dominate the model, however the choice to mean-center data is a modelling choice. Figure S1 provides a sensitivity analysis for the effect of mean-centering on Figure 2 in the main text.

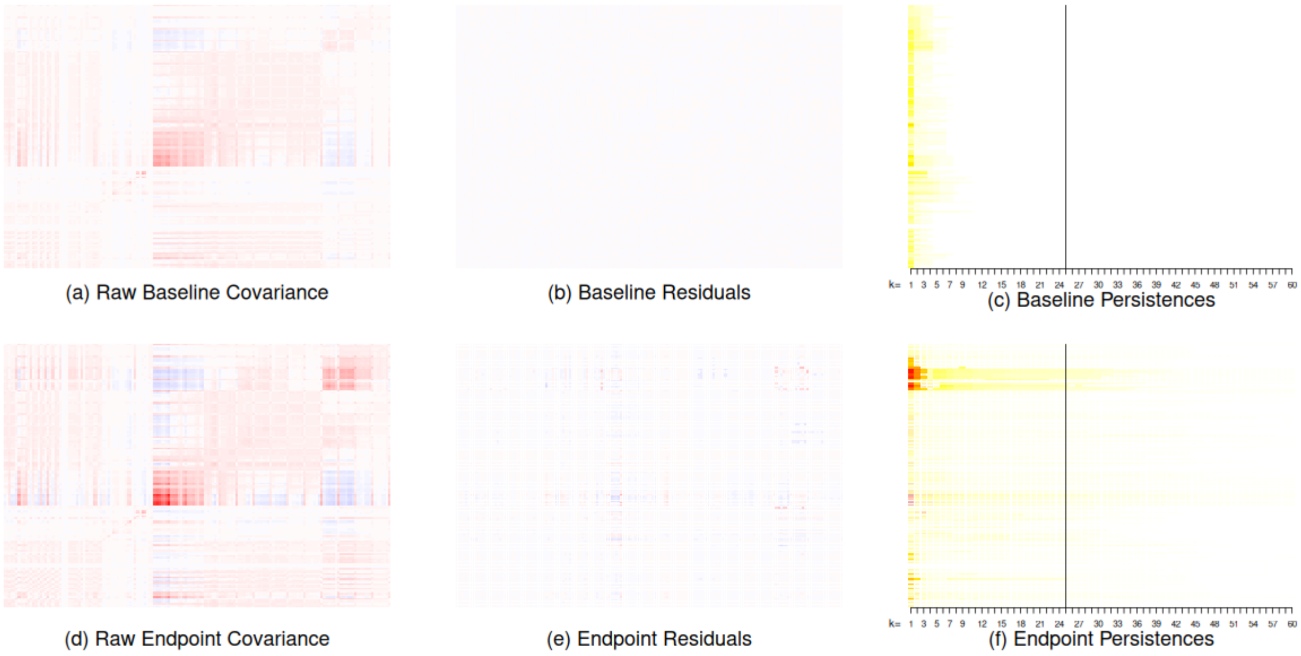

Figure S1 - Clarity Pipeline when raw data scaled but not mean-centered

When no mean-centering is applied, the means are included in the model. The structure is broadly similar but the strength of covariance within cluster structures is much diminished, with only a subset of metabolites identified as being significantly different from baseline to endpoint.

Notes on CLARITY test statistics

Because the data are relational, the original features are "reused" many times (once for each similarity calculation). The baseline data are then rotated to best match the endpoint, which induces further correlations between the values. To construct empirical distributions of test statistics CLARITY performs a repeated test/train data split both within the baseline and endpoint which are treated as pseudo-data. However, the correlations across covariances (from the rotation) and across replicates (from the reuse of the data) render traditional tests invalid. For example, even a simple sign test, where we set $d_r = y_r - x_r$ for the unsorted replicate differences between endpoint and baseline, and compare the observed value to the sign-randomized version, is not conservative.

We therefore construct a conservative above test statistic based on the Wilcoxon Rank Sum test to compare resampled distributions. This can generate a small p-value only if the entire distribution of the test statistic under the baseline is below the entire distribution of the endpoint. Because the number of summed similarities grows with the number of subjects, the probability of the entire endpoint distribution falling above the baseline by chance remains small (i.e. has an exponential tail), and the false positive rate under the null does not grow. This is demonstrated in Figure S2.

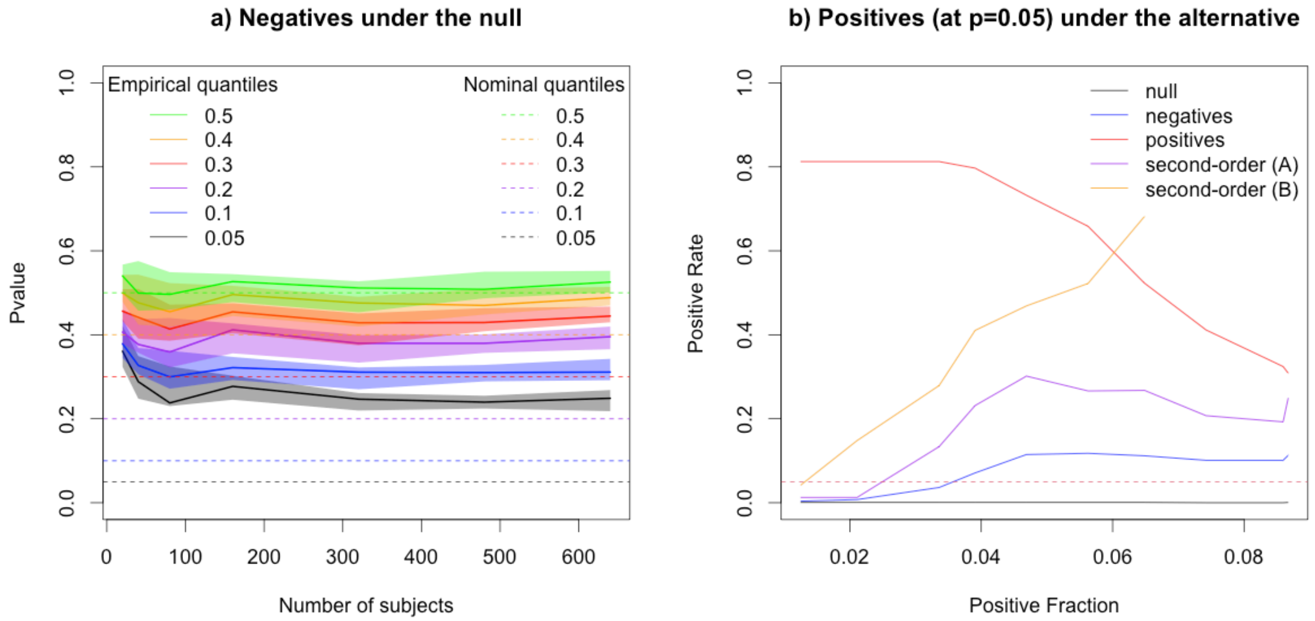

Figure S2- Positives in CLARITY based on the simulation framework of the ClaritySim package (Lawson et al. 2021) in a hierarchical model with K=10 clusters. Left: p-value distribution under a null with no positives is concentrated above p=0.2 even in the tail (black line: the family wise error rate with nominal p=0.05), leading to a family-wise error rate of less than 0.1% that does not change with the number of subjects. Right: Power under the alternative to detect positive cases (i.e. sensitivity) decreases as the positive fraction increases (red), and the overall p-values can become slightly non-conservative (blue), but the ranking remains reliable. In this situation 20% of nodes have a changed covariance, either by being in the cluster the positive cases move to (second-order A) or move from (second-order B).

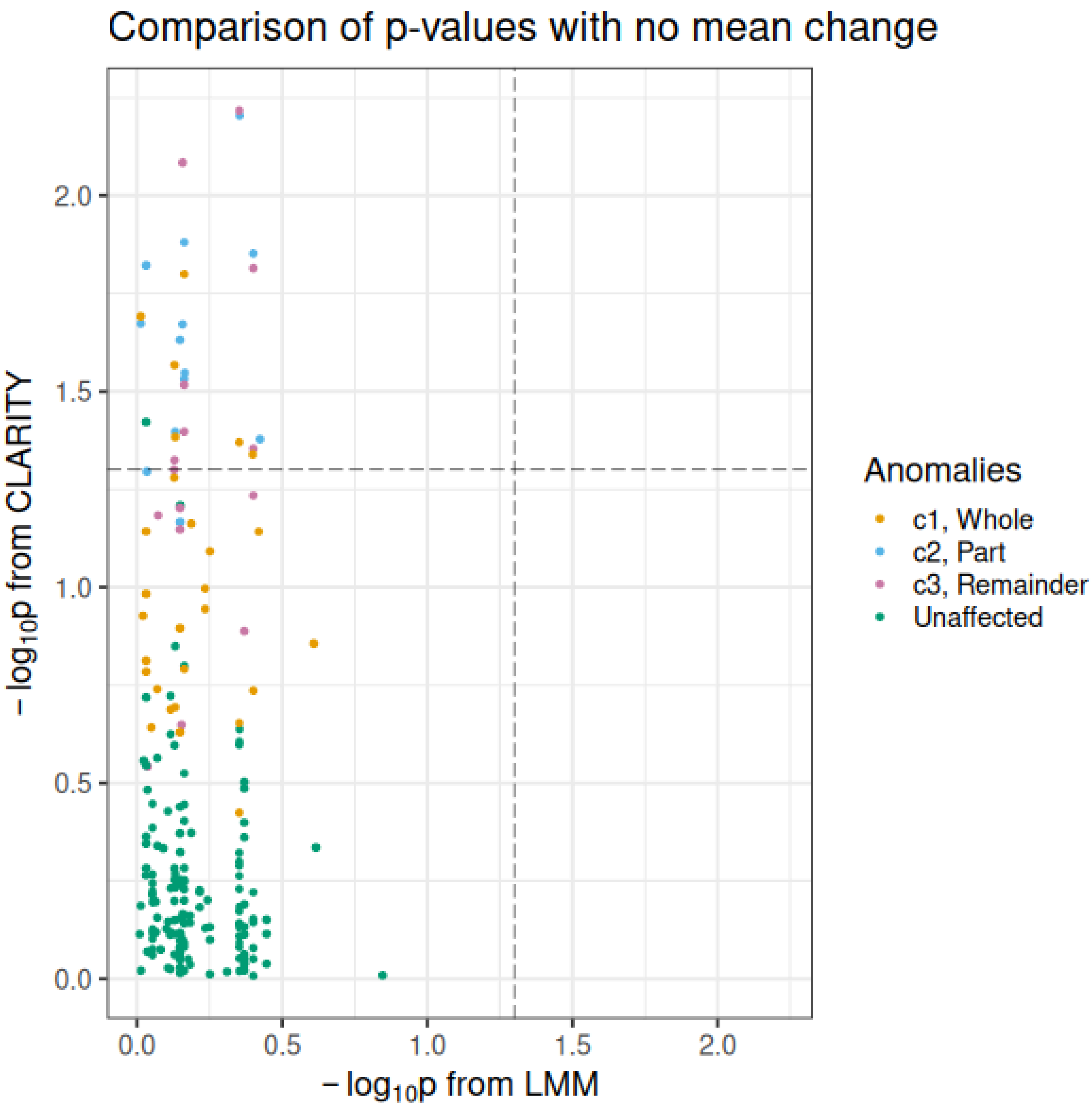

Figure S3 Comparison of p-values of simulated data when no mean change is introduced. The p-values of CLARITY remain unchanged, with the c2 and c3 clusters being identified as undergoing covariance change, whereas the LMM no longer finds any significant changes in metabolites.

References

Ahola-Olli, A. V., Mustelin, L., Kalimeri, M., Kettunen, J., Jokelainen, J., Auvinen, J., et al. (2019). Circulating metabolites and the risk of type 2 diabetes: A prospective study of 11,896 young adults from four Finnish cohorts. Diabetologia, 2298–2309. https://doi.org/10.1101/513648

Lawson, D. J., Solanki, V., Yanovich, I., Dellert, J., Ruck, D., & Endicott, P. (2021). CLARITY: comparing heterogeneous data using dissimilarity. Royal Society Open Science, 8(12), 202182. https://doi.org/10.1098/rsos.202182

Soininen, P., Kangas, A. J., Würtz, P., Suna, T., & Ala-Korpela, M. (2015). Quantitative serum nuclear magnetic resonance metabolomics in cardiovascular epidemiology and genetics. Circulation: Cardiovascular Genetics, 8(1), 192–206. https://doi.org/10.1161/CIRCGENETICS.114.000216
