# Supplementary material for: Multivariate analysis of metabolomic data to identify biological pathways modified by a clinical intervention
Source: Metabolomics. 2026 Jul 27;22(4):134. doi: 10.1007/s11306-026-02490-w (PMC13407567; doi:10.1007/s11306-026-02490-w)
Supplement: Supplementary file 4 — Supplementary Material 4 [file 11306_2026_2490_MOESM4_ESM.pdf]

Online resource 4 for:

Multivariate analysis of metabolomic data to identify biological pathways modified by a clinical intervention

*Rachel M. Wood[1], *Laura J. Corbin[2,3], Jane M. Blazeby[4,5], Chris A. Rogers[6], Nicholas J. Timpson[2,3], Daniel J. Lawson[1,3]

Table of Contents

Supplementary Figures *3*

Supplementary Figure 1 3

Supplementary Figure 2 4

Supplementary Figure 3 5

Supplementary Figures

Supplementary Figure 1

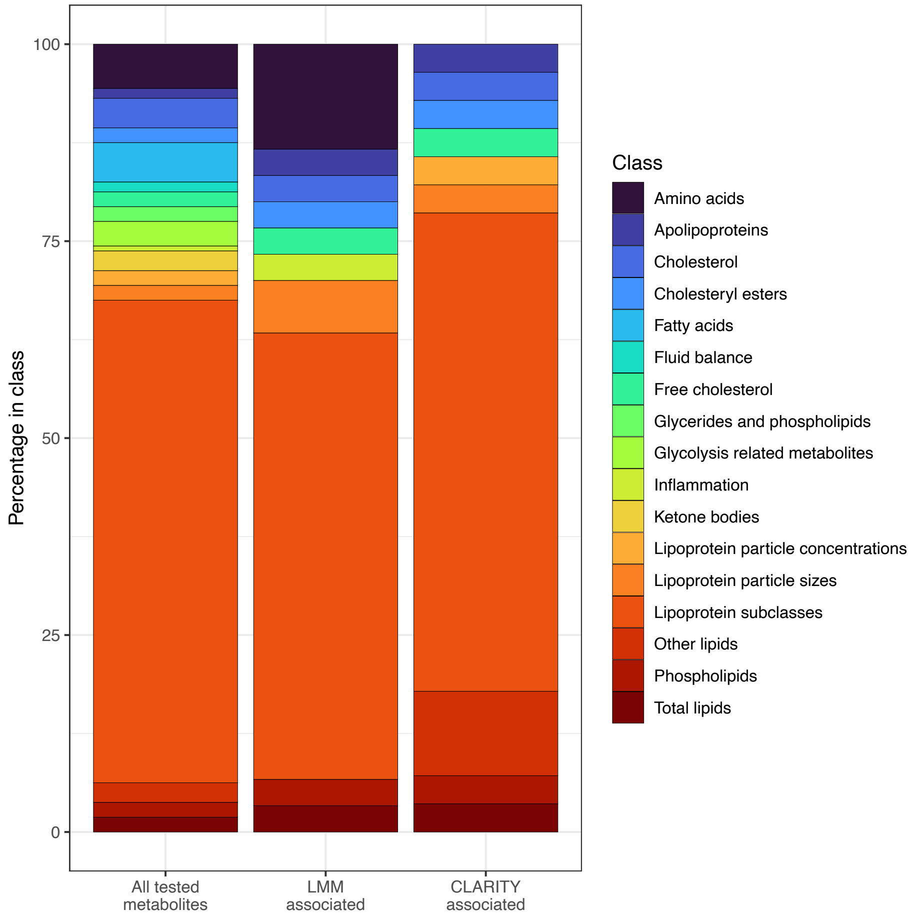

Distribution across classes of intervention-associated metabolites identified by the two different methods, as compared to all metabolites tested.

LMM = linear mixed model.

Supplementary Figure 2

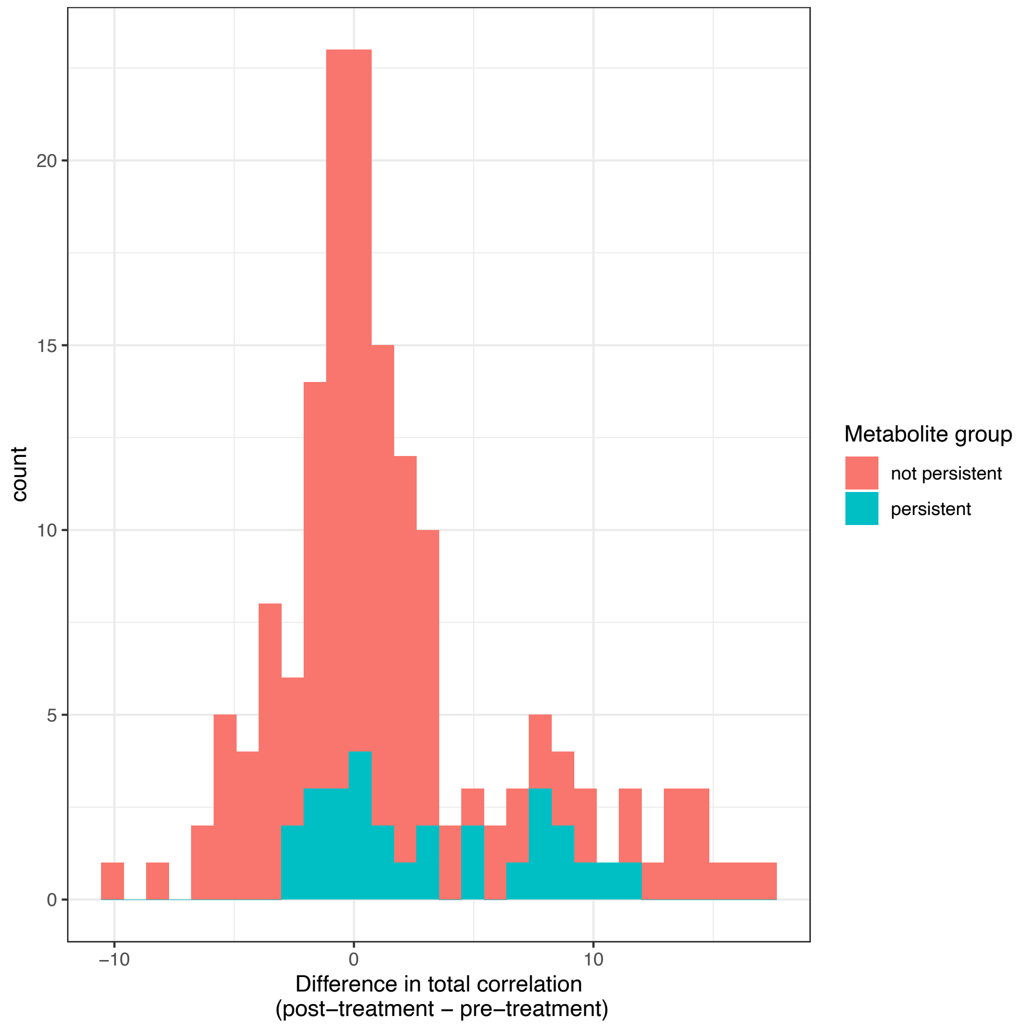

Distribution of change in total correlation for each metabolite (post-treatment – pre-treatment).

Coloured according to whether metabolites showed persistence in CLARITY or not.

Supplementary Figure 3

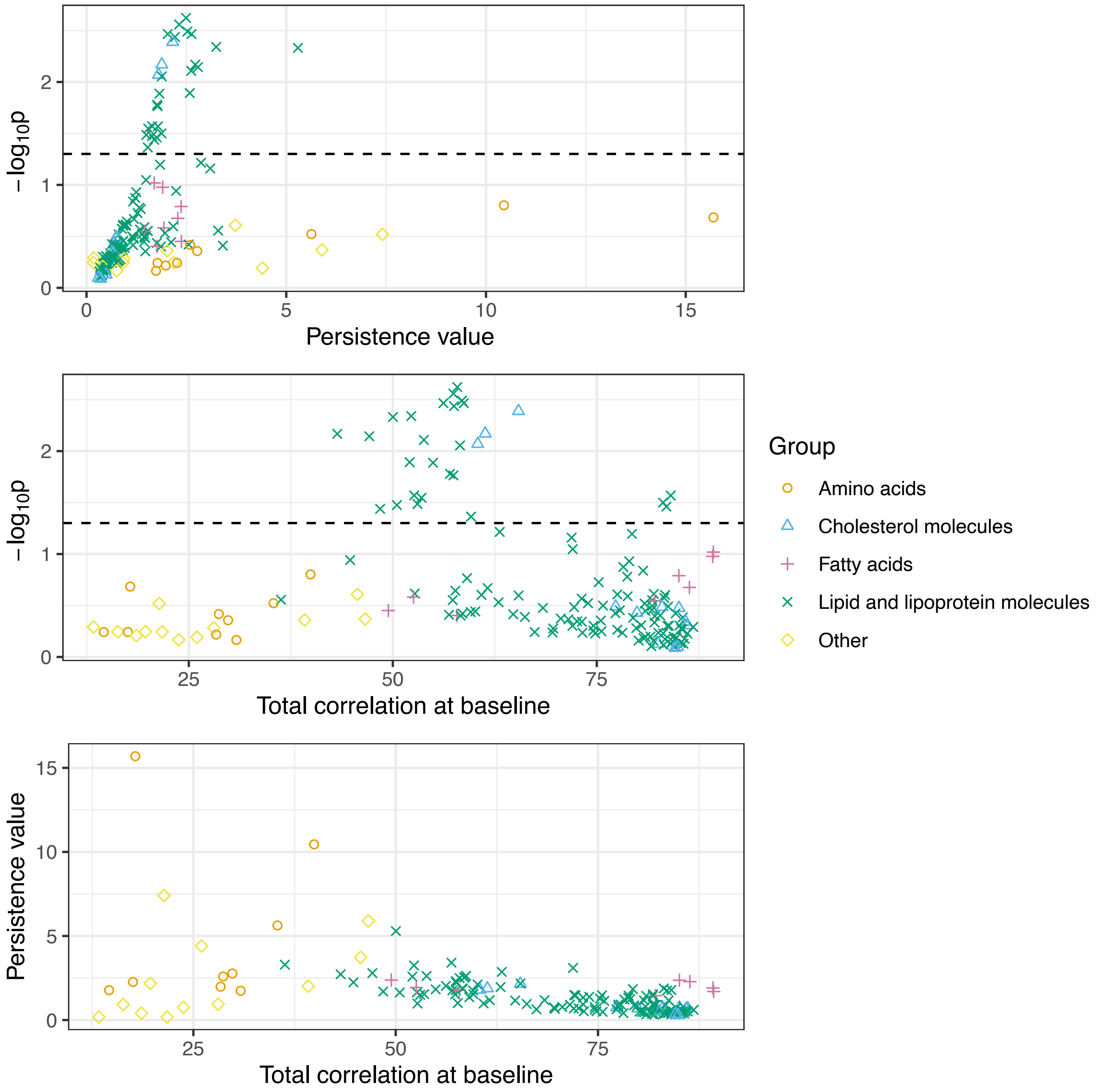

Exploration of CLARITY results.

Top: Plot of -log10(p) on persistence score (CLARITY)

Middle: Plot of -log10(p) from CLARITY on total metabolite correlation at baseline.

Bottom: Plot of persistence score from CLARITY on total metabolite correlation at baseline.
