# Supplementary material for: Multivariate analysis of metabolomic data to identify biological pathways modified by a clinical intervention
Source: Metabolomics. 2026 Jul 27;22(4):134. doi: 10.1007/s11306-026-02490-w (PMC13407567; doi:10.1007/s11306-026-02490-w)
Supplement: Supplementary file 5 — Supplementary Material 5 [file 11306_2026_2490_MOESM5_ESM.pdf]

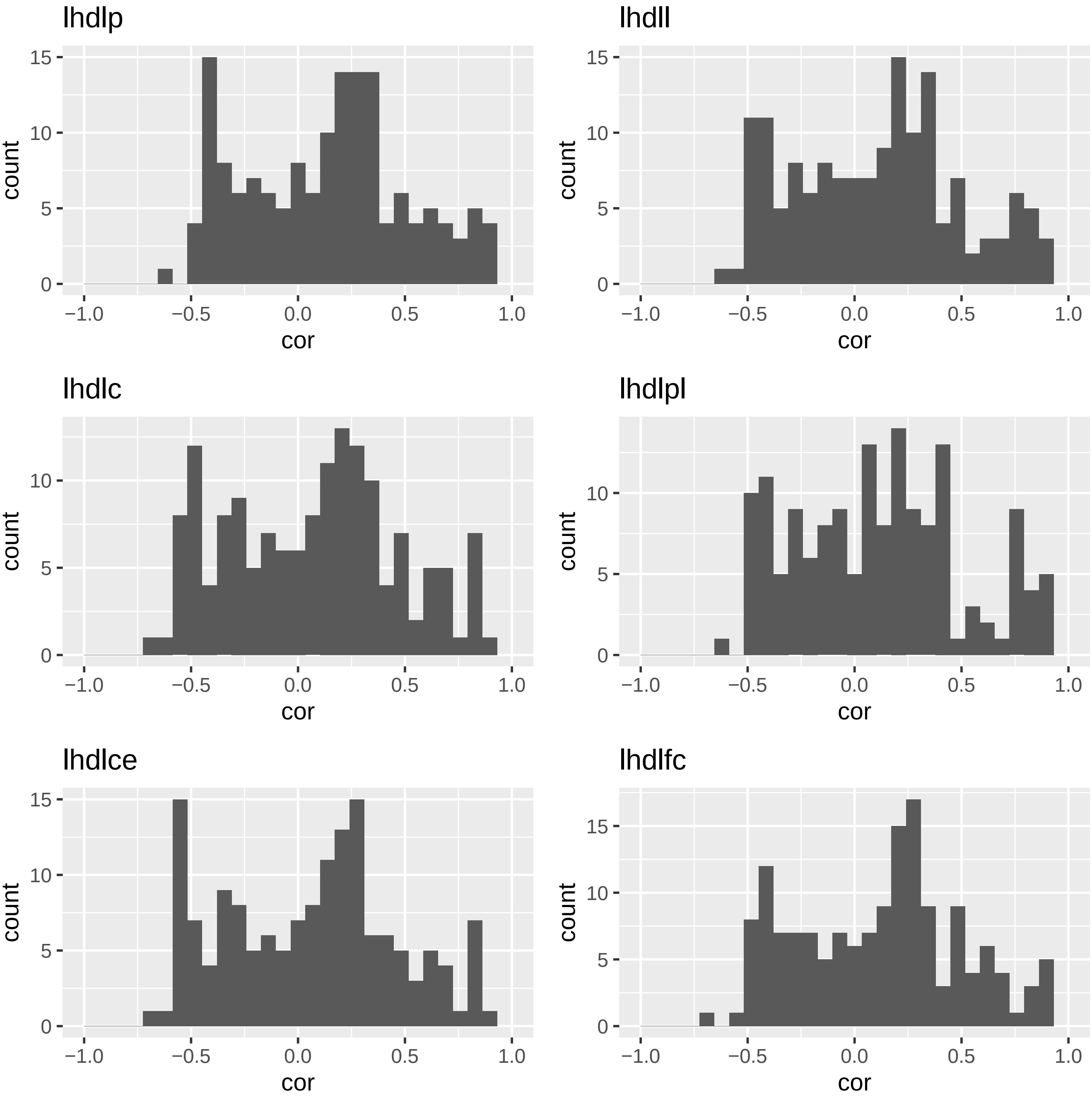
lhdlp
lhdll
lhdlc
lhdlpl
lhdlce
lhdlfc
count
cor
−1.0
−0.5
0.0
0.5
1.0
0
5
10
15

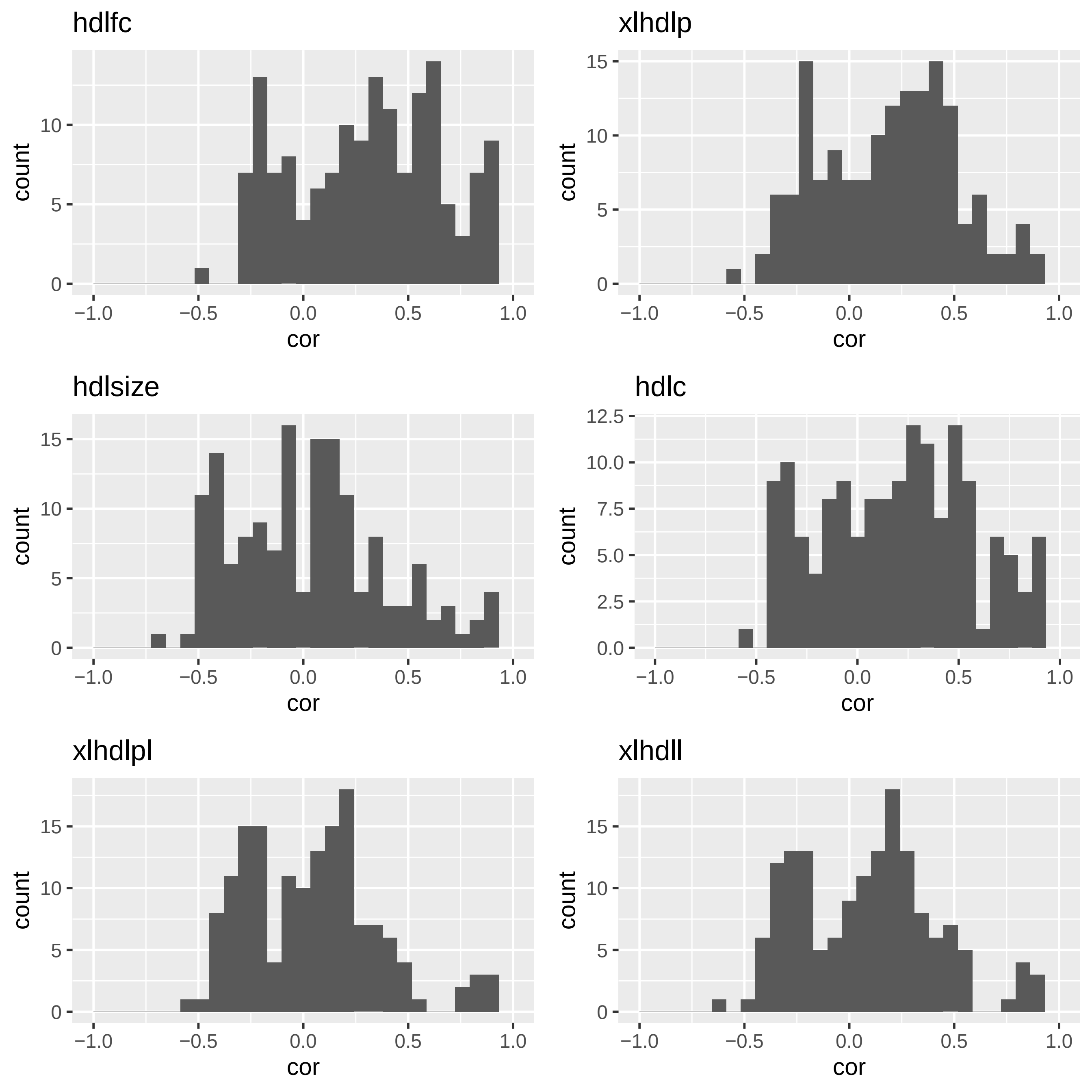
hdlfc
xlhdlp
hdlsize
hdlc
xlhdlpl
xlhdll
count
cor
0
5
10
15
0.0
2.5
5.0
7.5
10.0
12.5
−1.0
−0.5
0.0
0.5
1.0

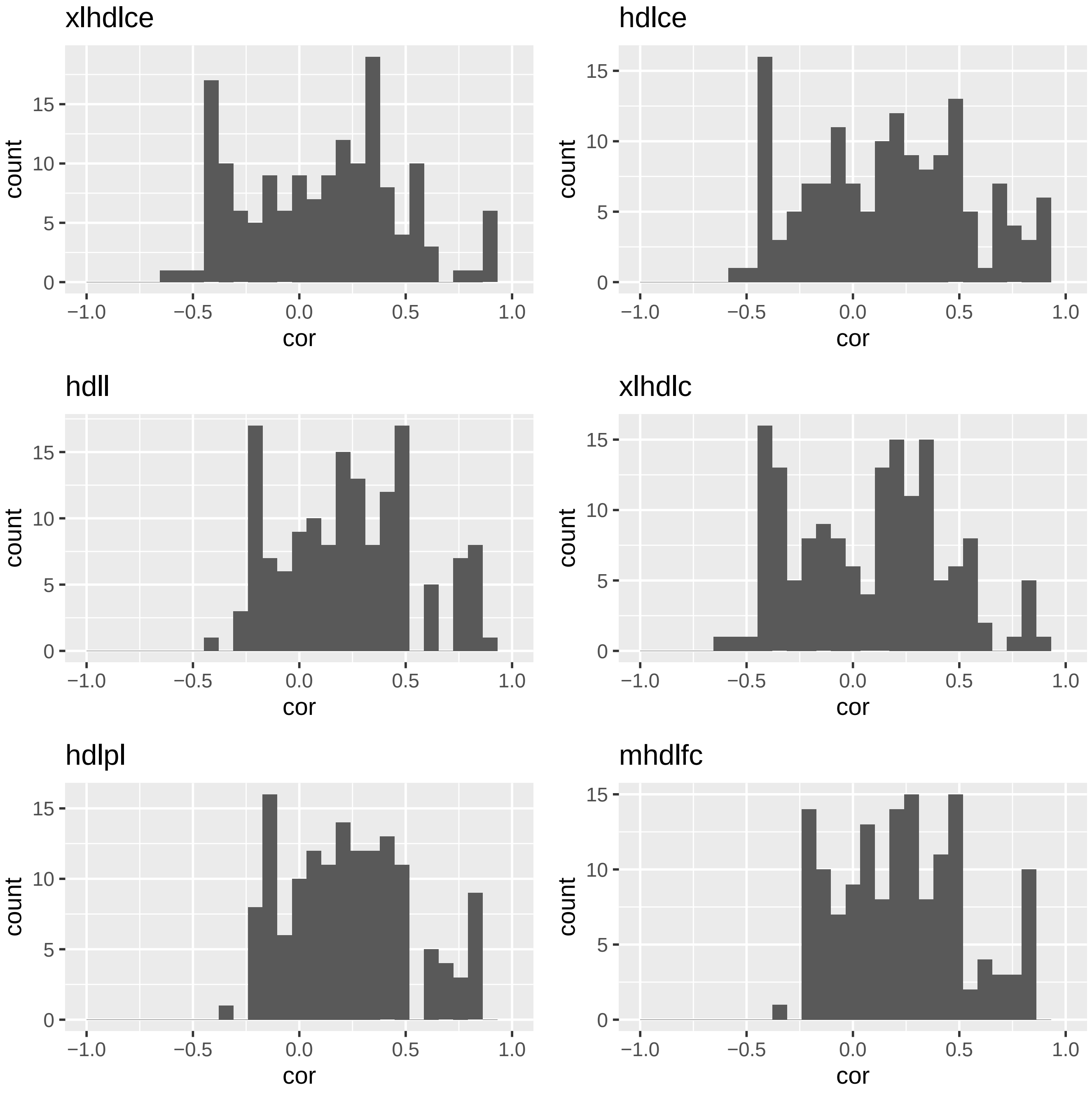
xlhdlce
hdlce
hdll
xlhdlc
hdlpl
mhdlfc
count
cor
0
5
10
15
−1.0
−0.5
0.0
0.5
1.0

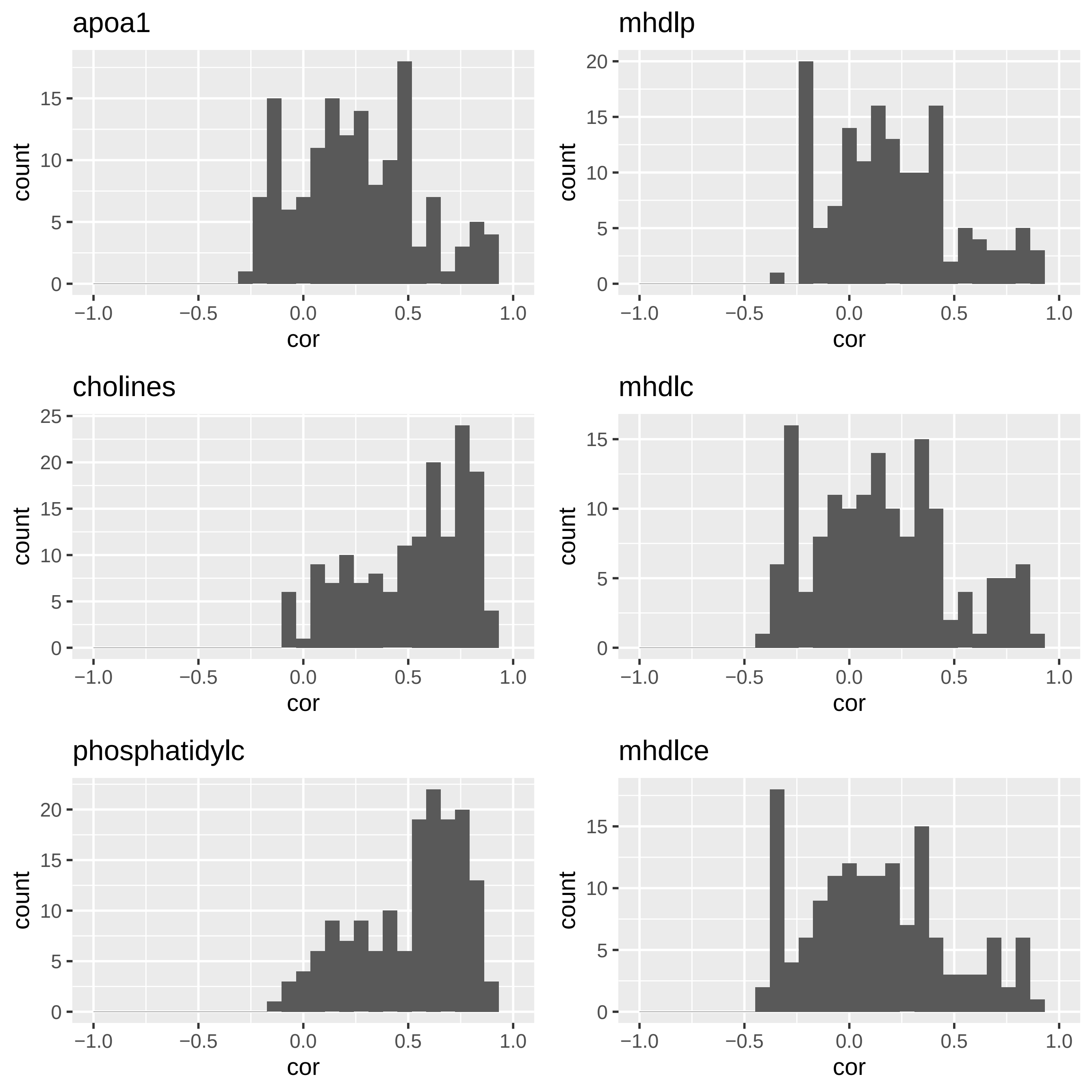
apoa1
mhdlp
cholines
mhdlc
phosphatidylc
mhdlce
count
cor
−1.0
−0.5
0.0
0.5
1.0
0
5
10
15
20
25

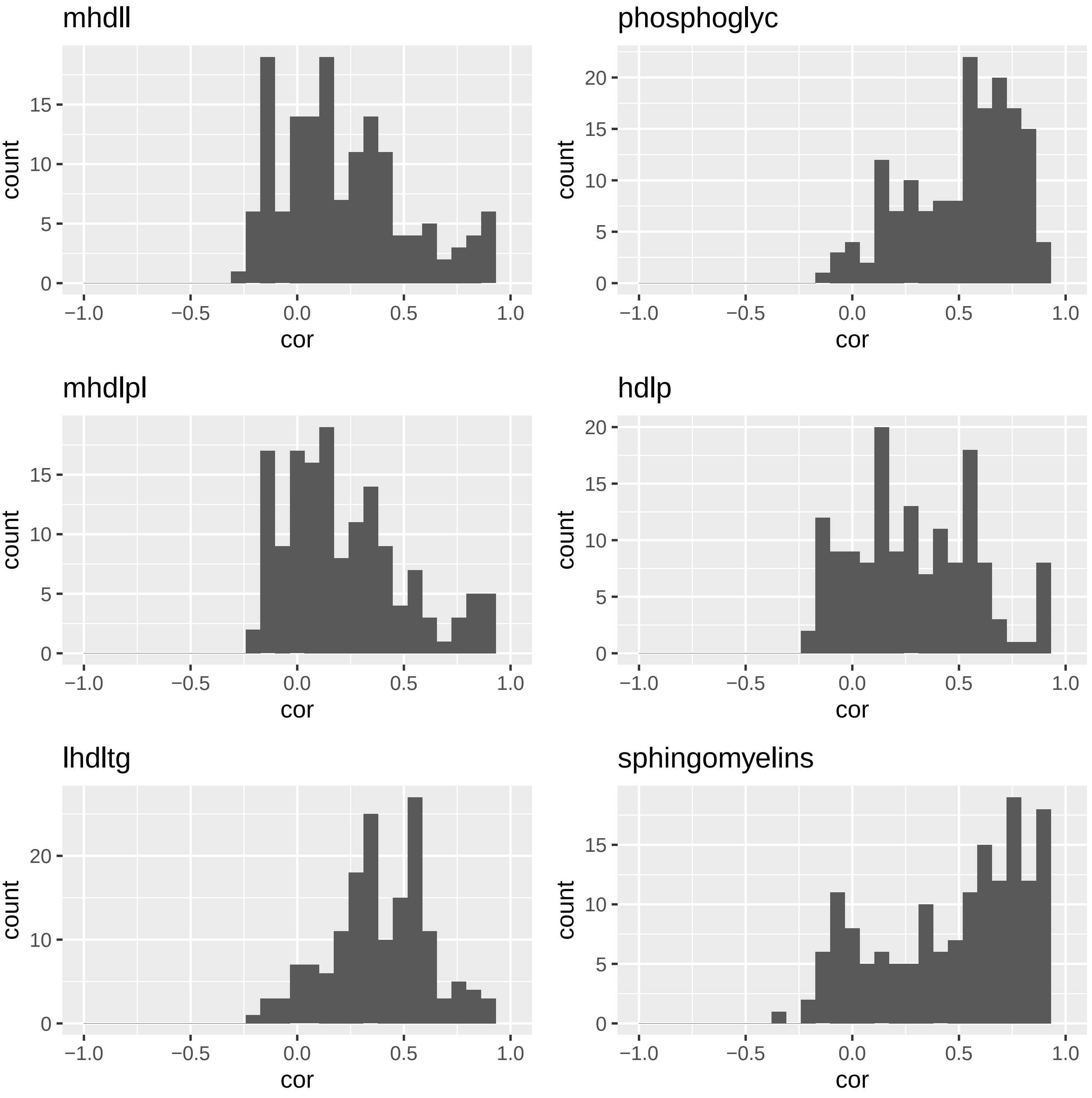
mhdll
phosphoglyc
mhdlpl
hdlp
lhdltg
sphingomyelins
count
cor
−1.0
−0.5
0.0
0.5
1.0
0
5
10
15
20

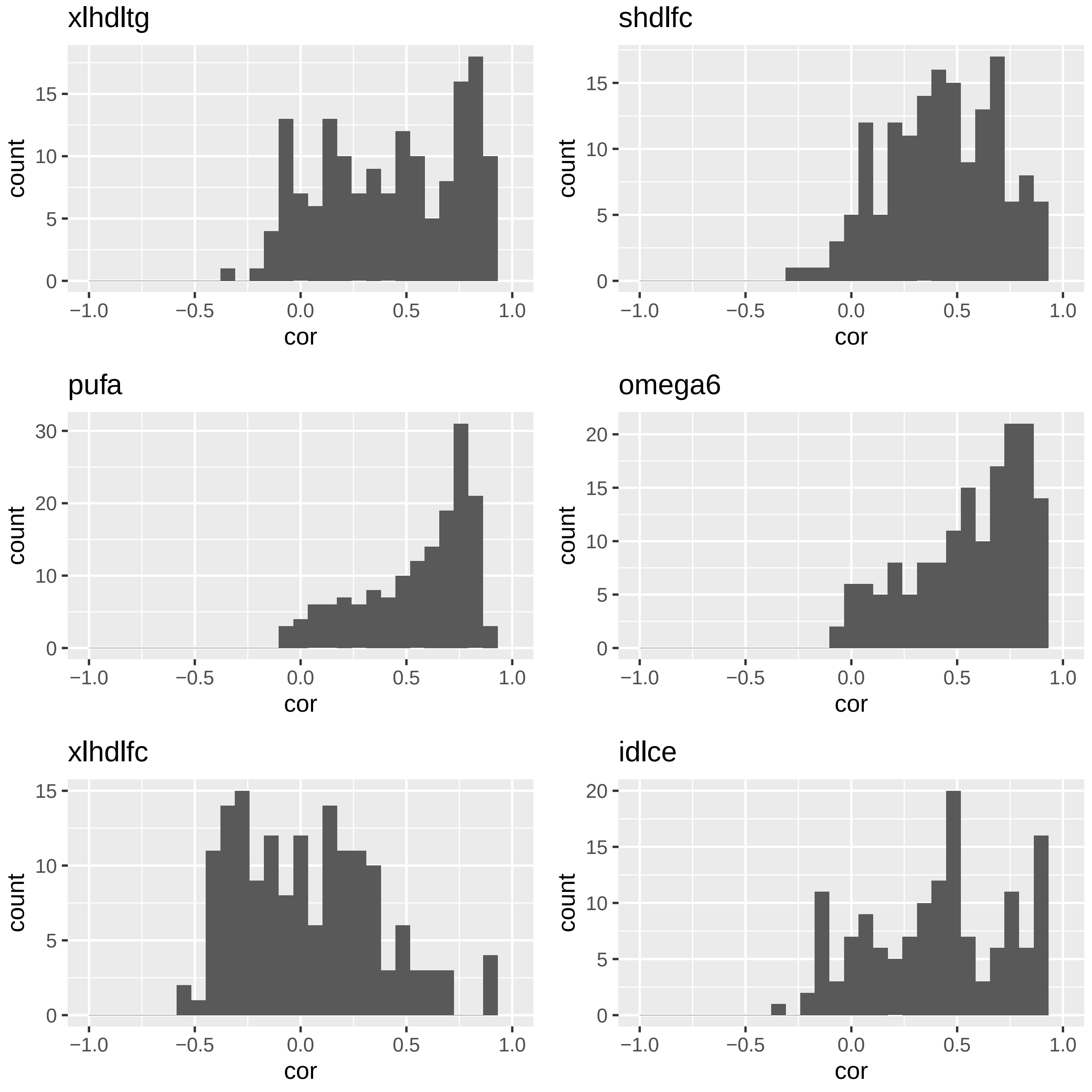
xlhdltg
shdlfc
pufa
omega6
xlhdlfc
idlce
count
cor
−1.0
−0.5
0.0
0.5
1.0
0
5
10
15
20
30

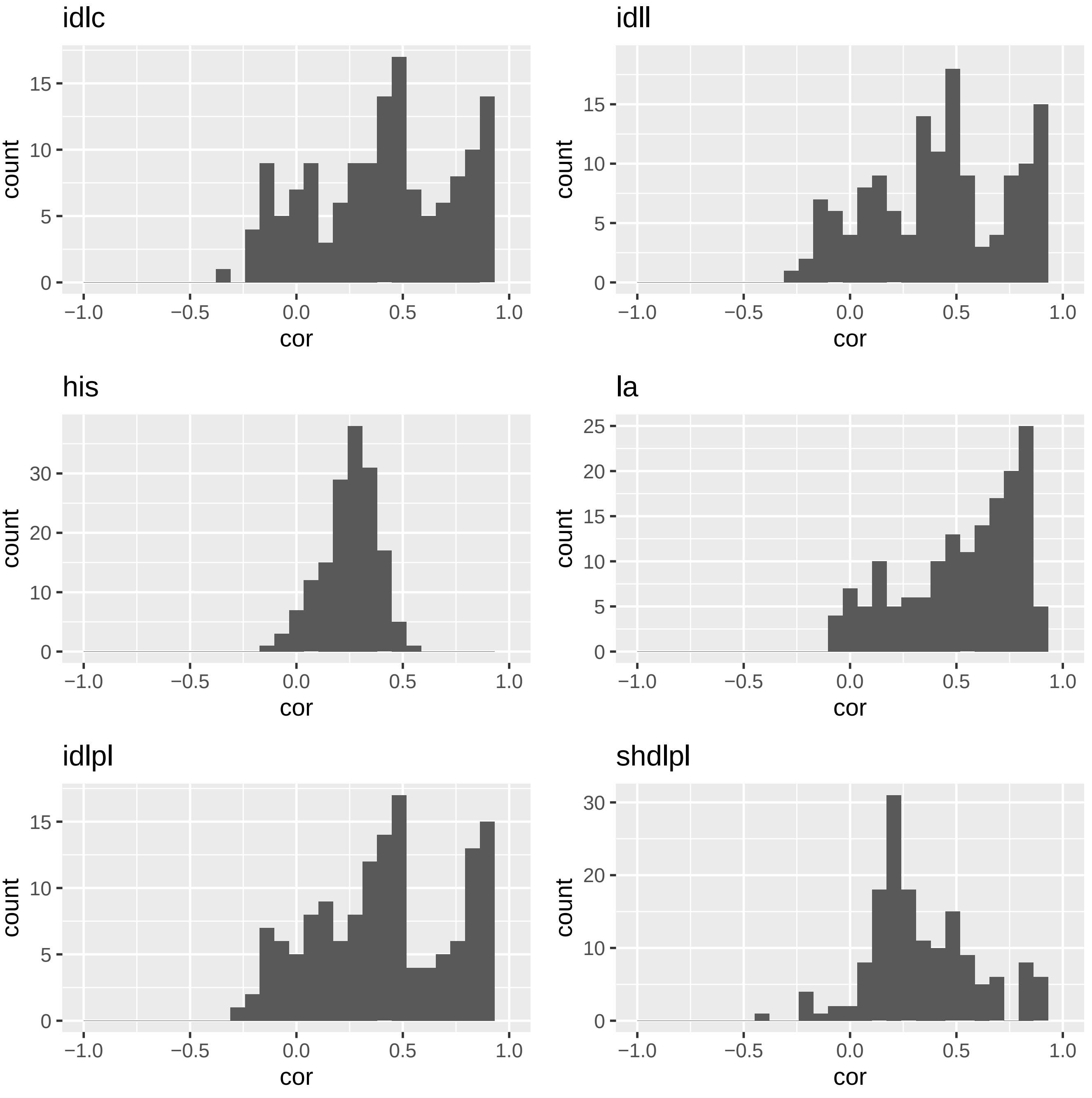
idlc
idll
his
la
idlpl
shdlpl
count
cor
−1.0
−0.5
0.0
0.5
1.0
0
5
10
15
20
25
30

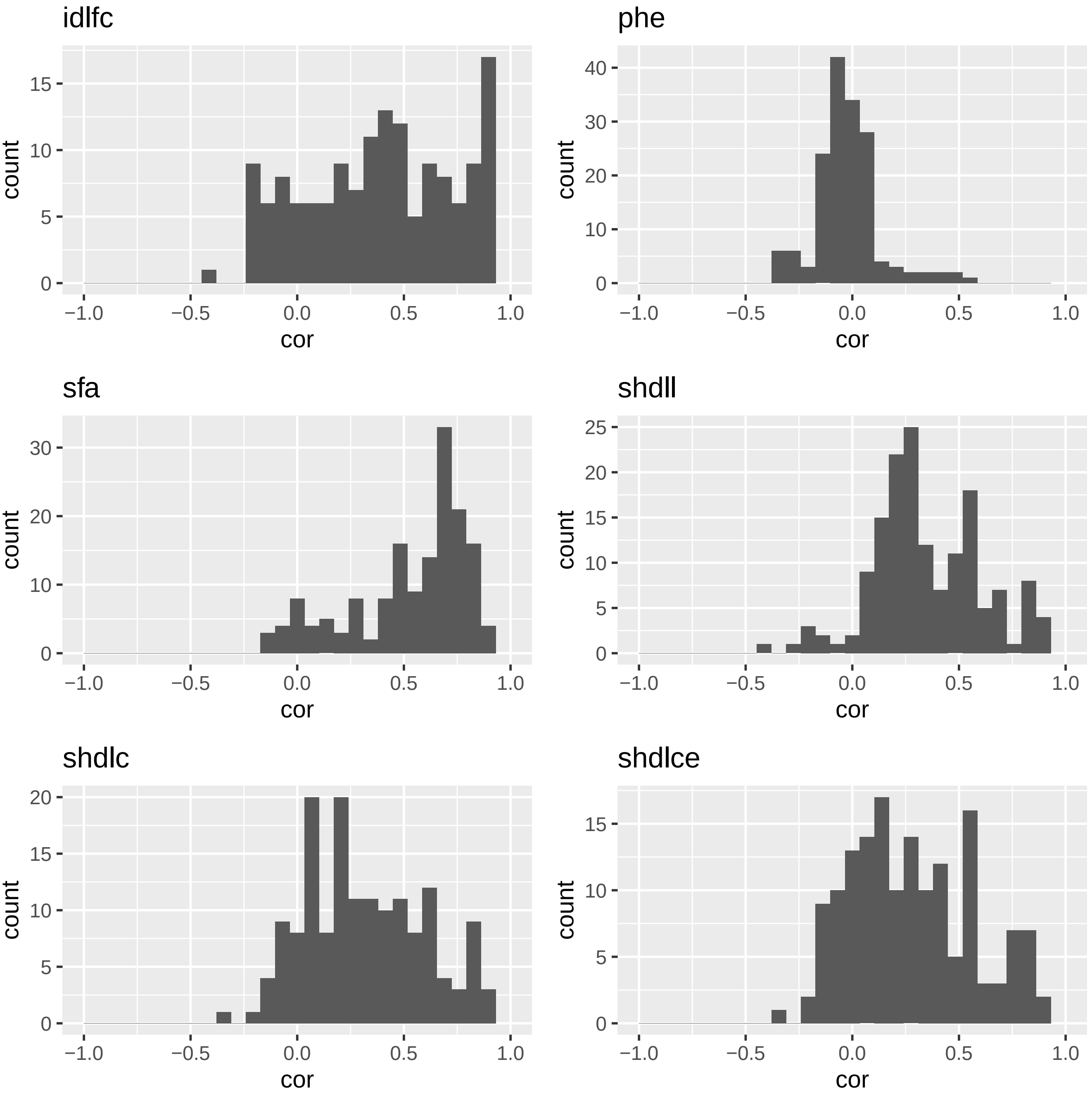
idlfc
phe
sfa
shdll
shdlc
shdlce
count
cor
−1.0
−0.5
0.0
0.5
1.0

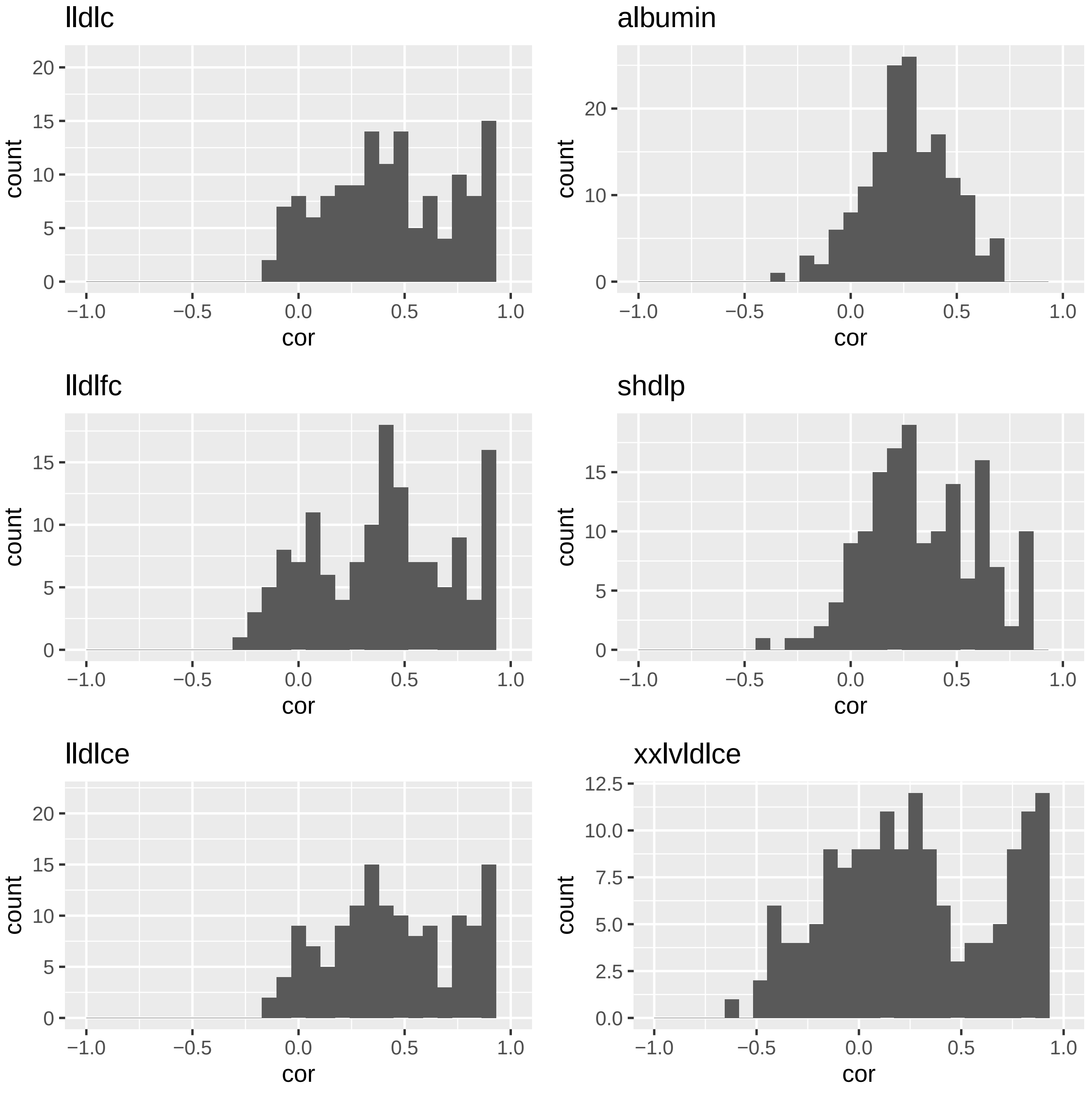
lldlc
albumin
lldlfc
shdlp
lldlce
xxlvldlce
count
cor
−1.0
−0.5
0.0
0.5
1.0
0
5
10
15
20
0.0
2.5
5.0
7.5
10.0
12.5

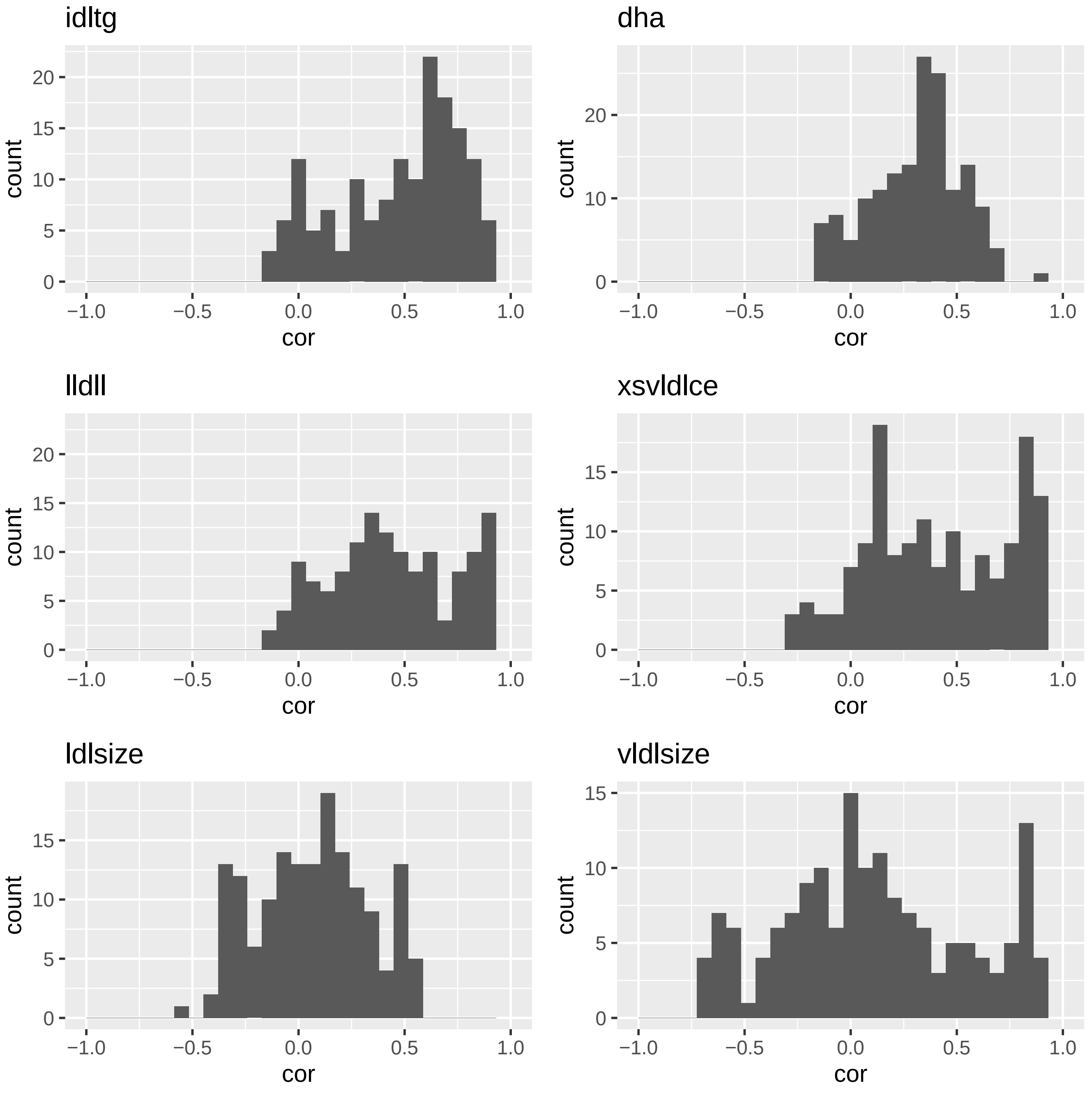
idltg
dha
lldll
xsvldlce
ldlsize
vldlsize
count
cor
−1.0
−0.5
0.0
0.5
1.0
0
5
10
15
20

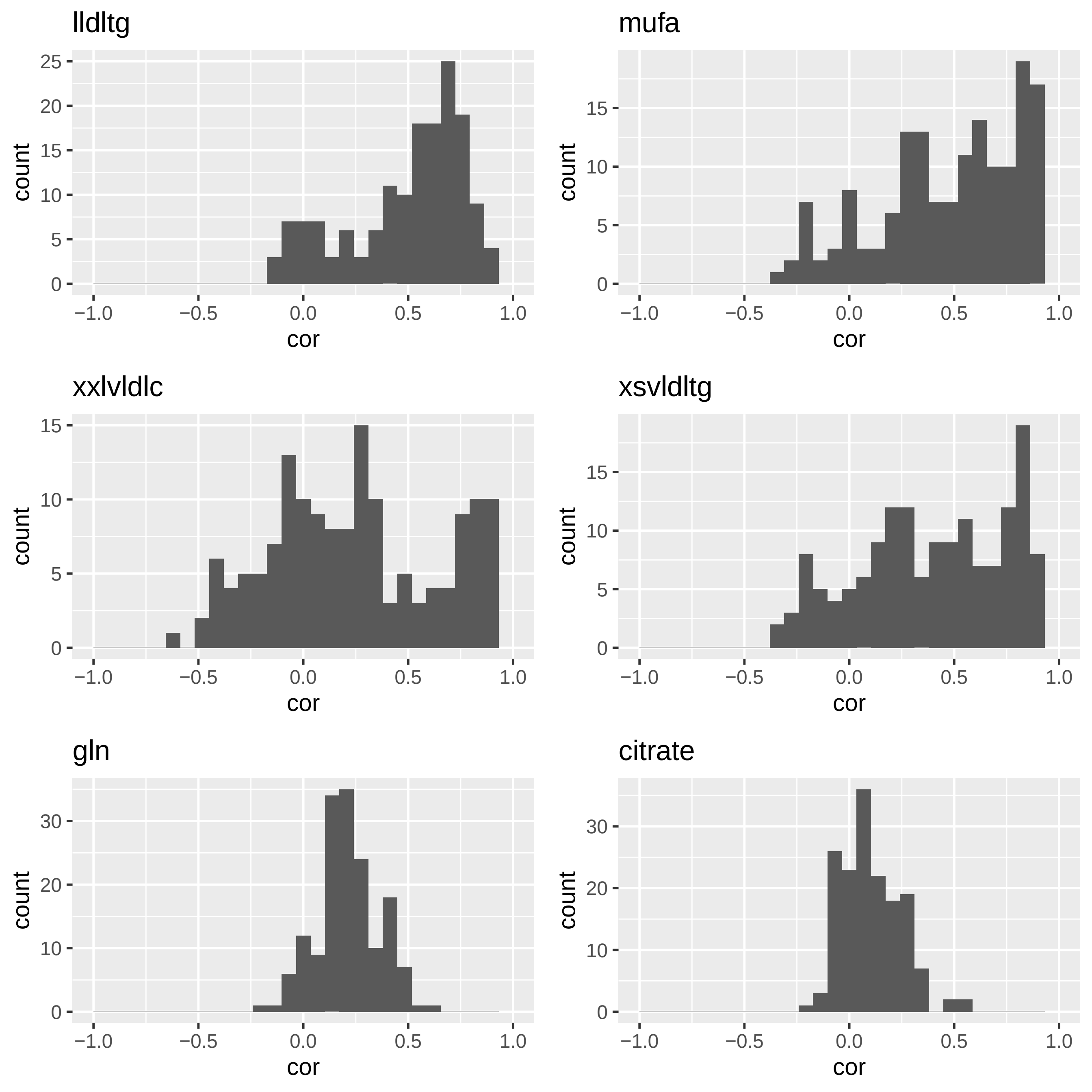
lldltg
mufa
xxlvldlc
xsvldltg
gln
citrate
count
cor
−1.0
−0.5
0.0
0.5
1.0
0
5
10
15
20
25
30

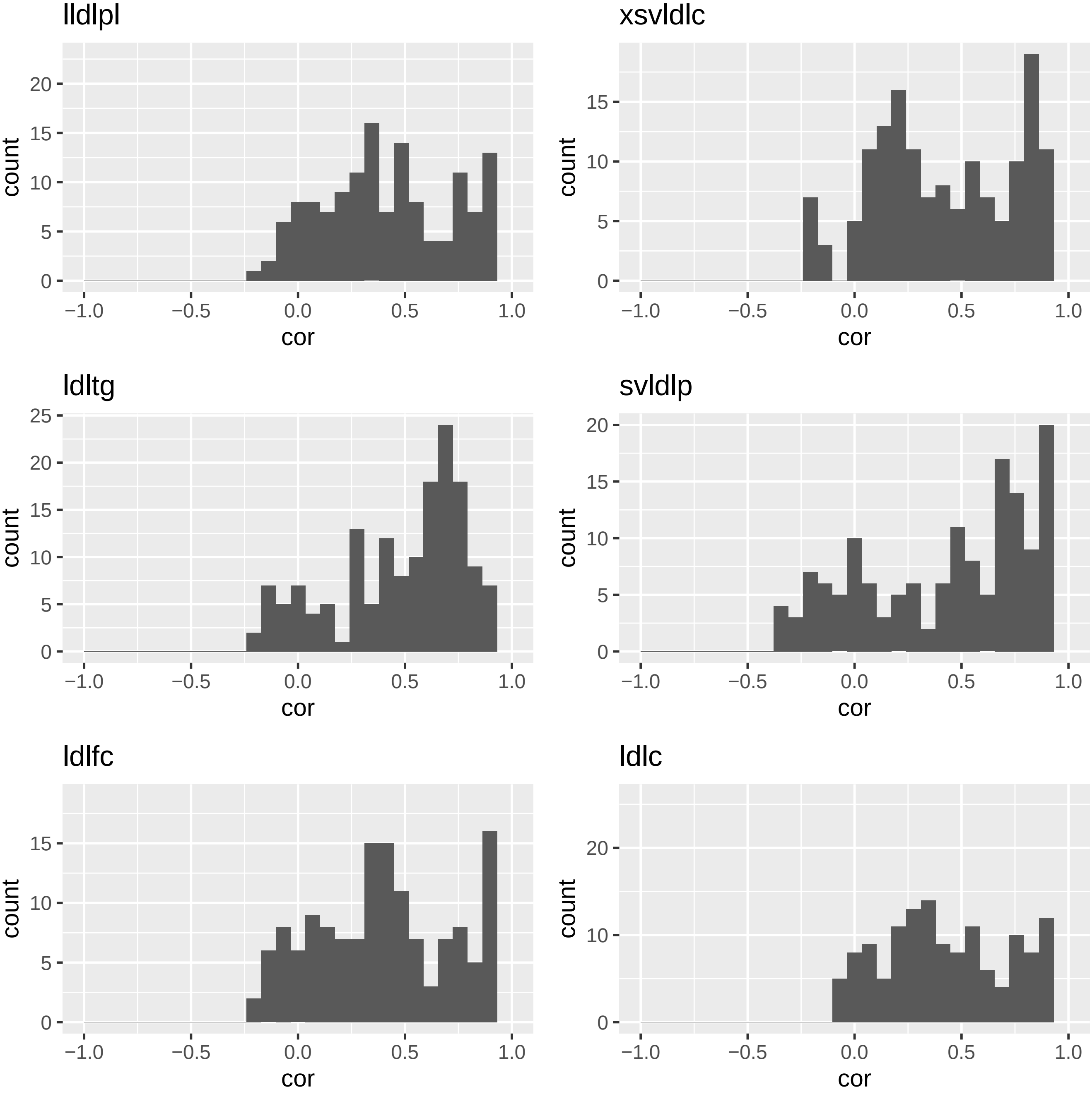
lldlpl
xsvldlc
ldltg
svldlp
ldlfc
ldlc
count
cor
−1.0
−0.5
0.0
0.5
1.0
0
5
10
15
20
25

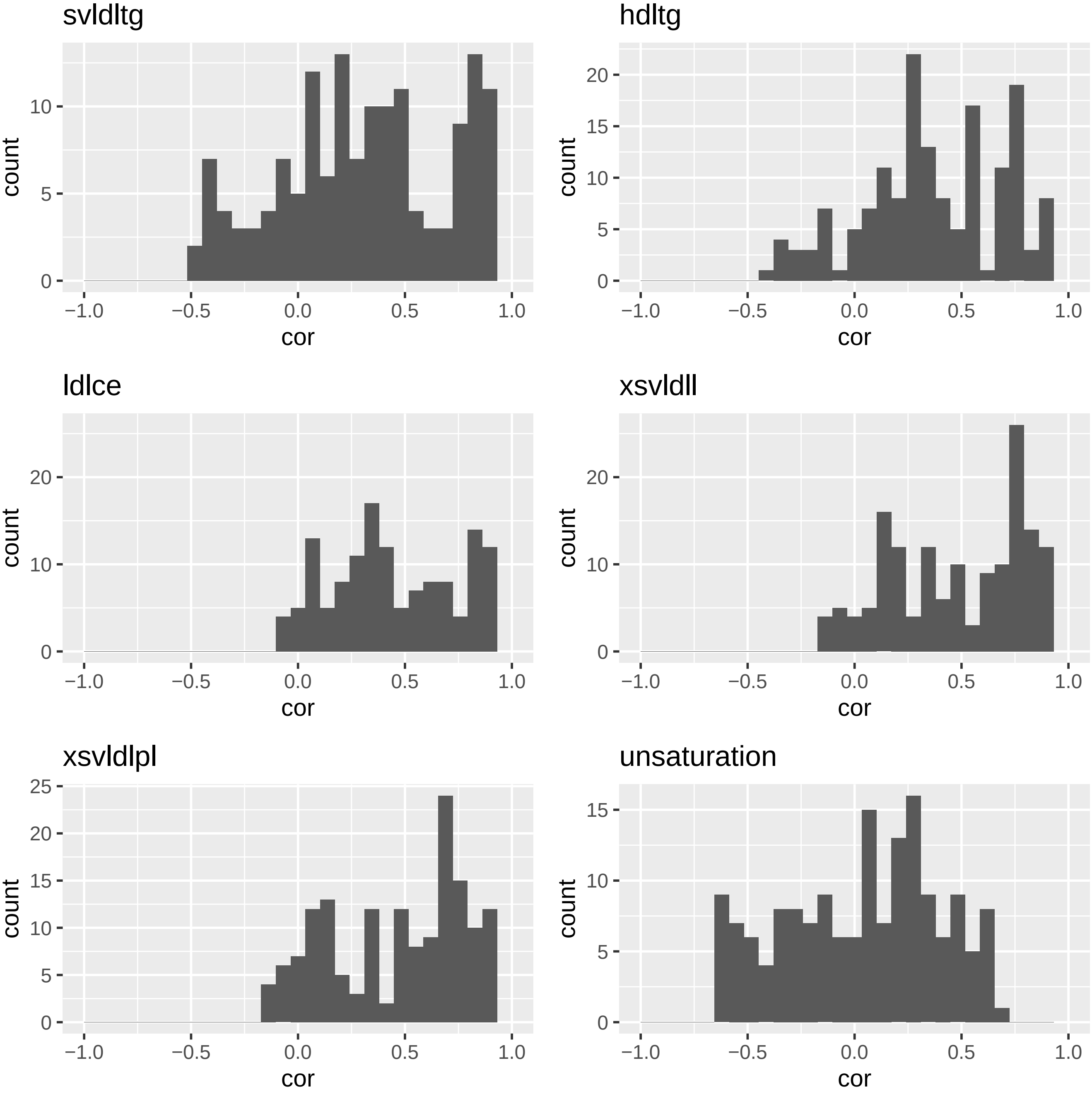
svldltg
hdltg
ldlce
xsvldll
xsvldlpl
unsaturation
count
cor
−1.0
−0.5
0.0
0.5
1.0

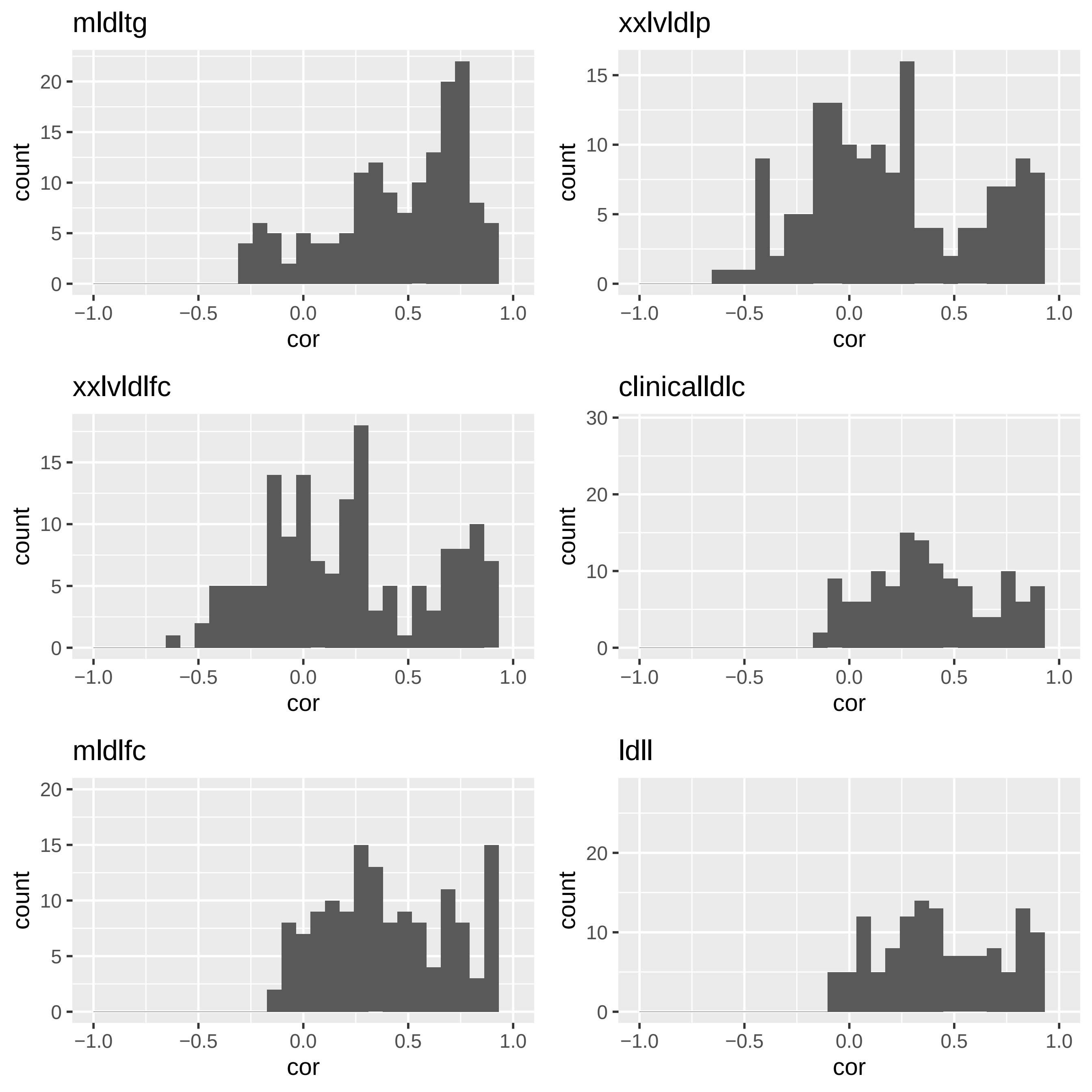

mldltg
xxlvldlp
xxlvldlfc
clinicalldlc
mldlfc
ldll
count
cor

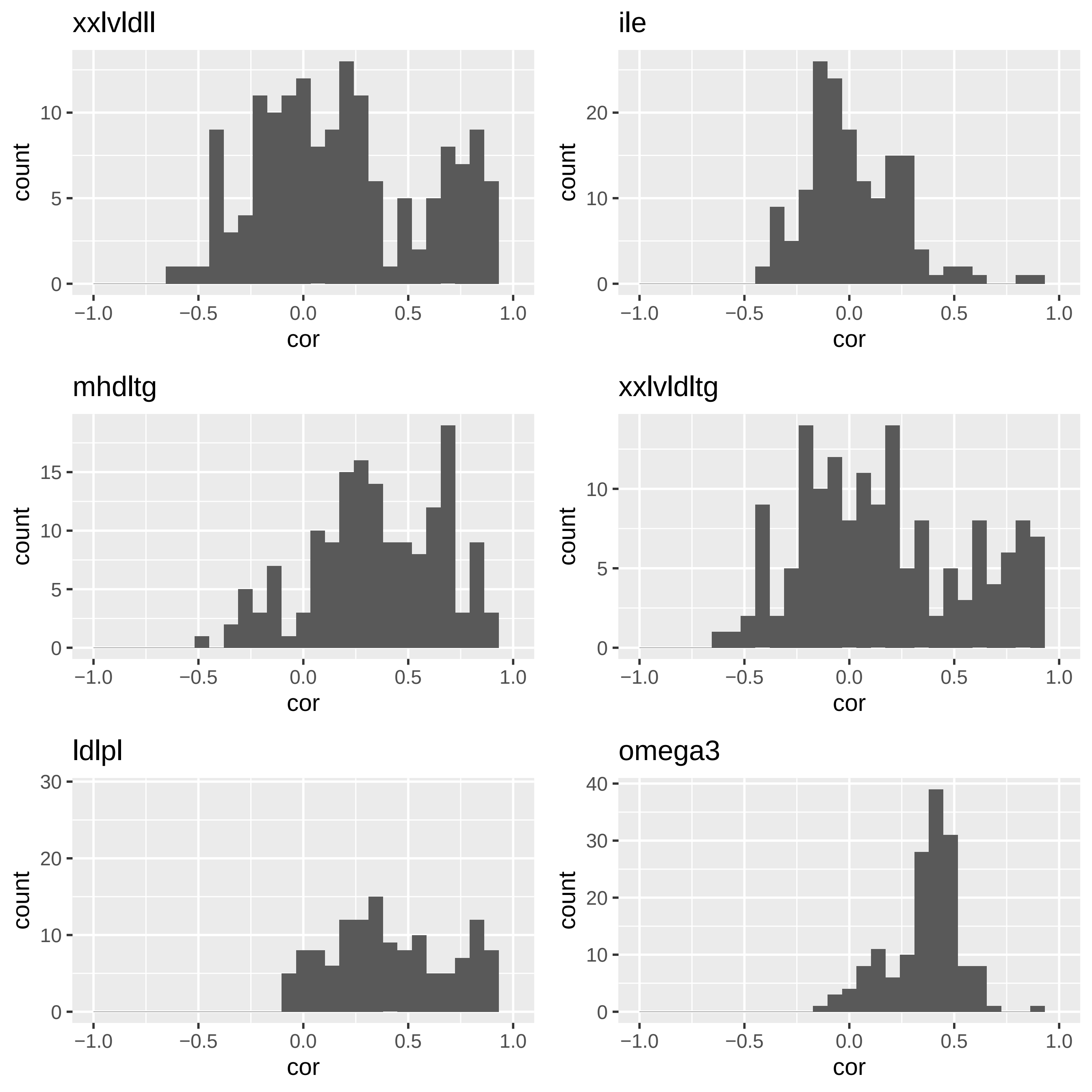
xxlvldll
ile
mhdltg
xxlvldltg
ldlpl
omega3
count
cor
−1.0
−0.5
0.0
0.5
1.0
0
5
10
15
20
30
40

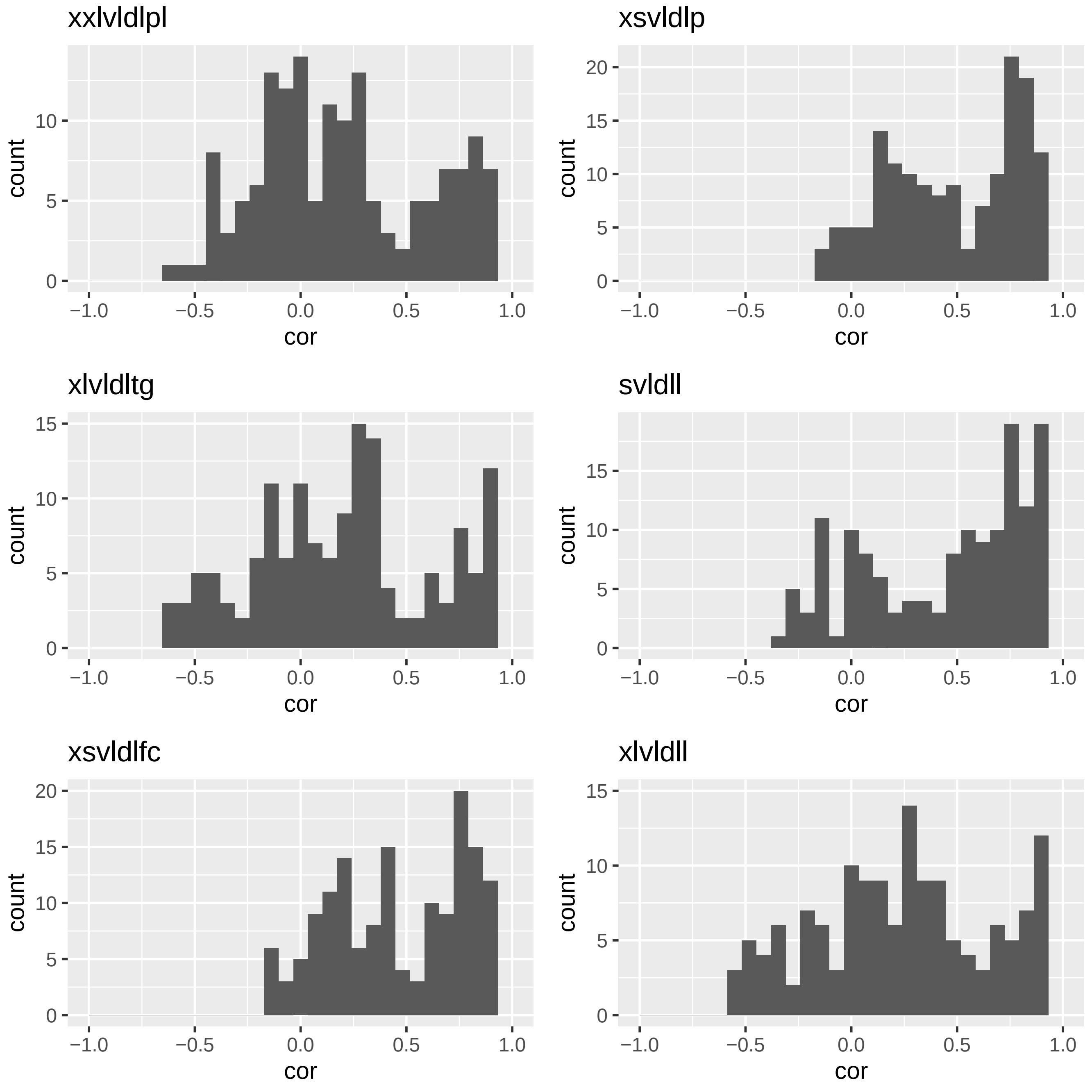
xxlvldlpl
xsvldlp
xlvldltg
svldll
xsvldlfc
xlvldll
count
cor
−1.0
−0.5
0.0
0.5
1.0
0
5
10
15
20

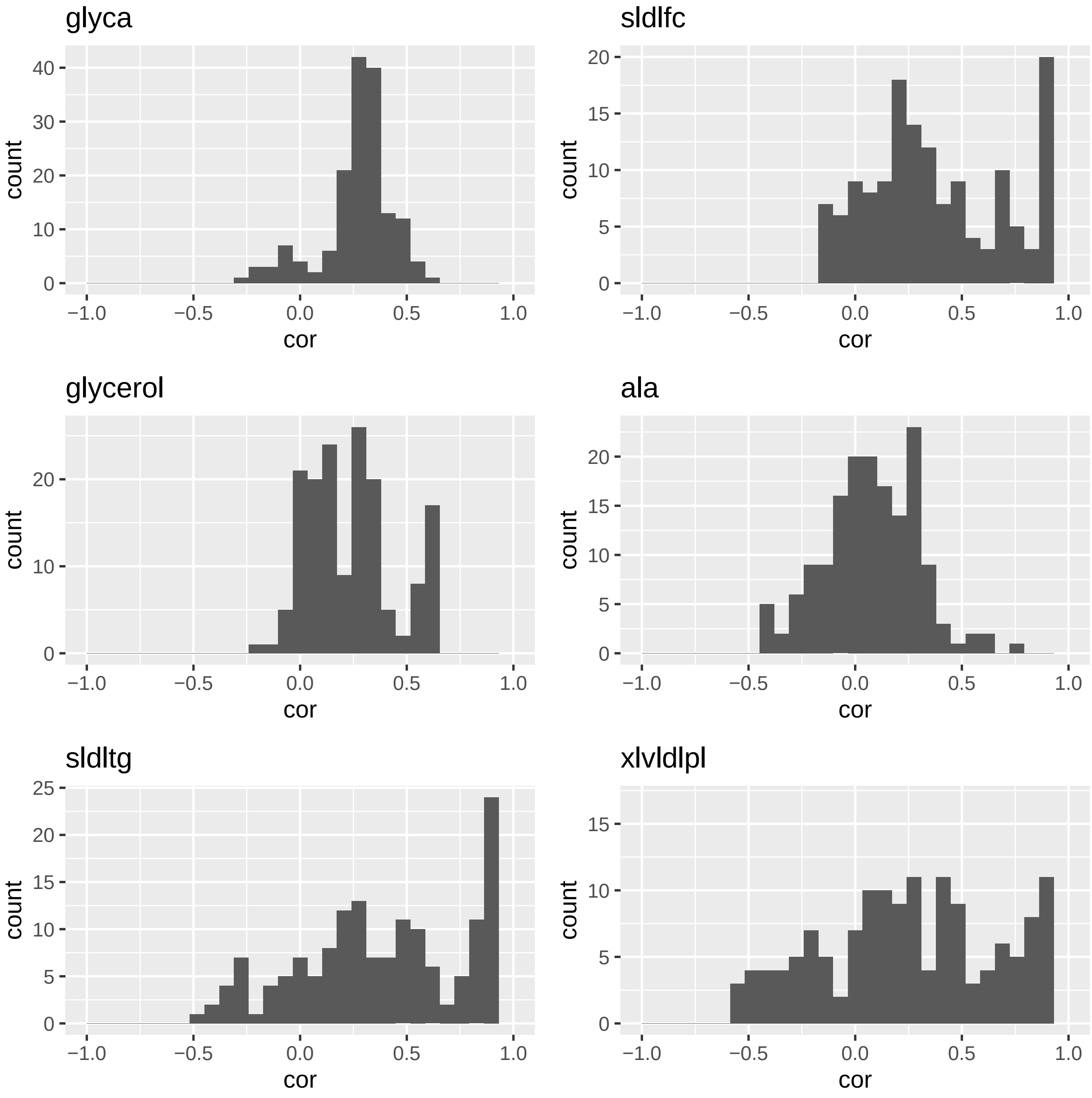
glyca
count
40
30
20
10
0
−1.0
−0.5
0.0
0.5
1.0
cor
sldlfc
count
20
15
10
5
0
−1.0
−0.5
0.0
0.5
1.0
cor
glycerol
count
20
10
0
−1.0
−0.5
0.0
0.5
1.0
cor
ala
count
20
15
10
5
0
−1.0
−0.5
0.0
0.5
1.0
cor
sldltg
count
25
20
15
10
5
0
−1.0
−0.5
0.0
0.5
1.0
cor
xlvldlpl
count
15
10
5
0
−1.0
−0.5
0.0
0.5
1.0
cor

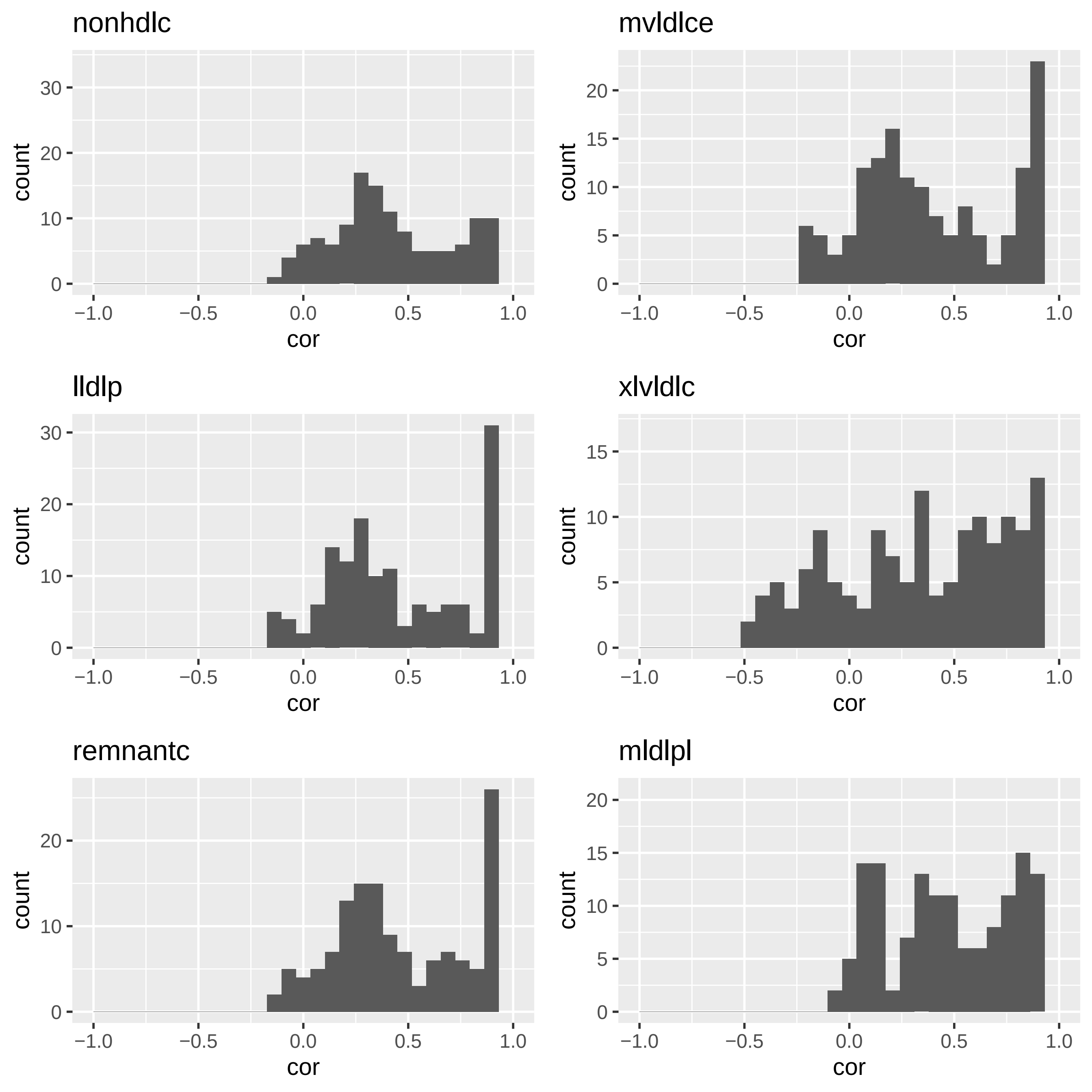
nonhdlc
mvldlce
lldlp
xlvldlc
remnantc
mldlpl
count
cor
−1.0
−0.5
0.0
0.5
1.0

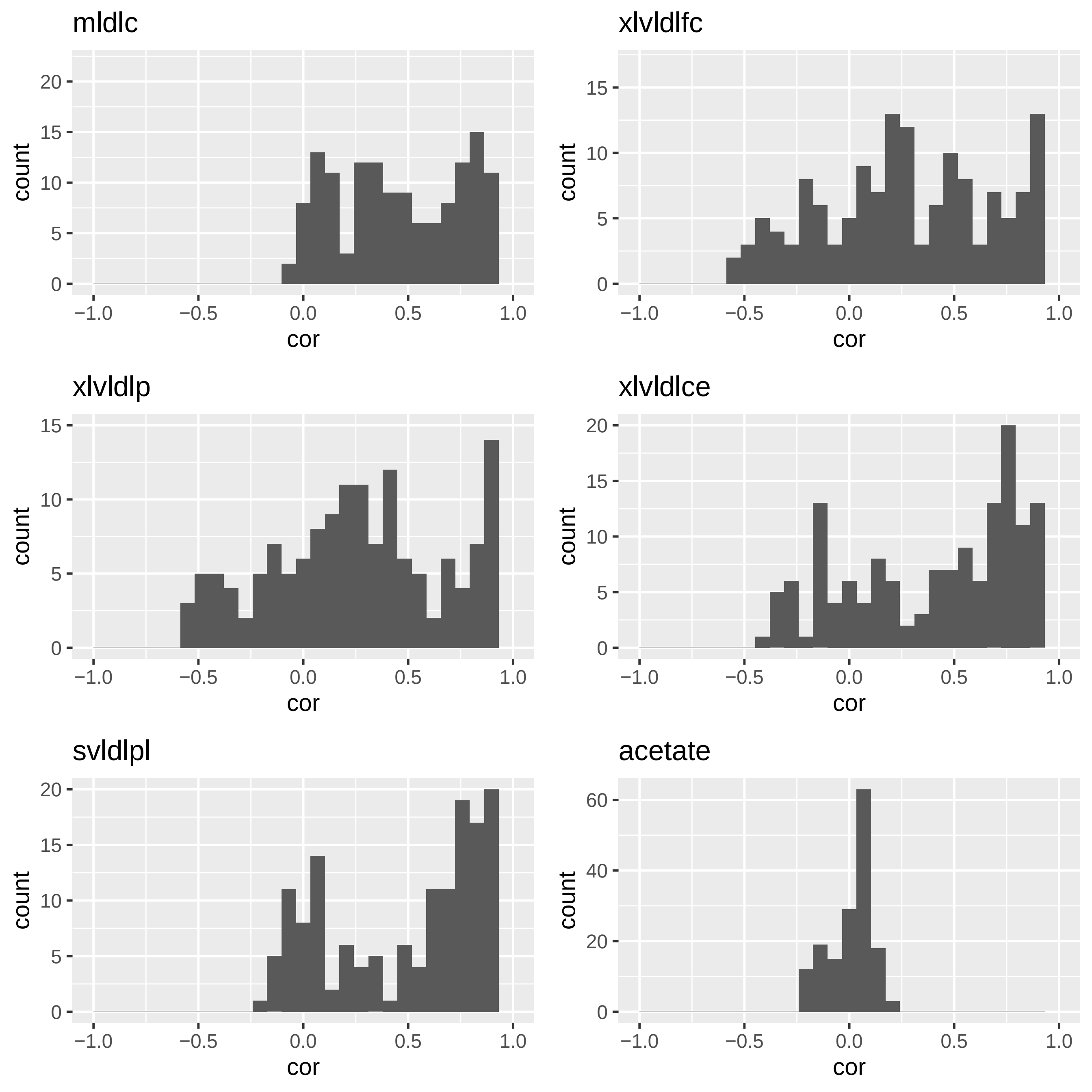

mldlc
xlvldlfc
xlvldlp
xlvldlce
svldlpl
acetate
count
cor

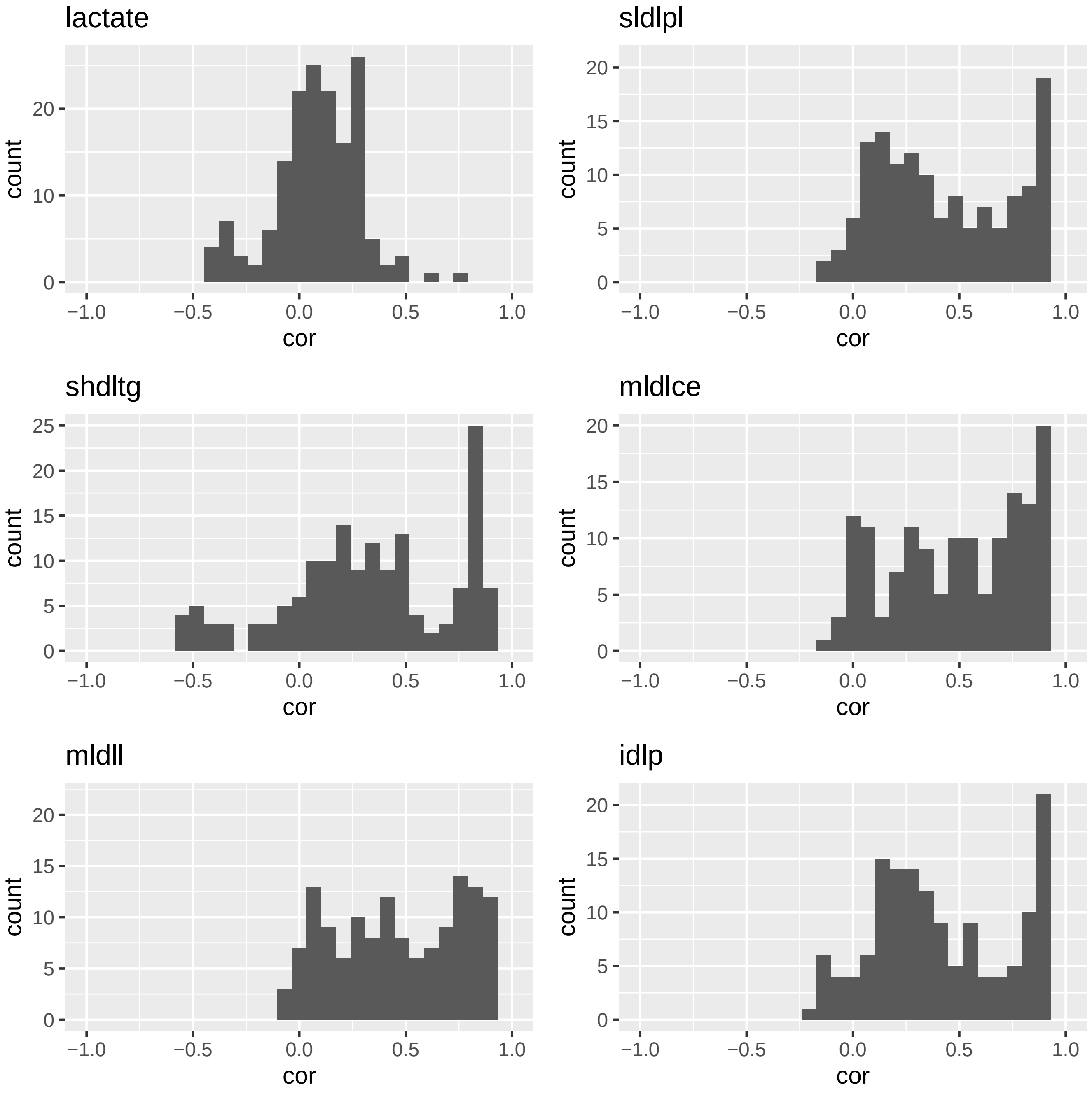
lactate
sldlpl
shdltg
mldlce
mldll
idlp
count
cor
−1.0
−0.5
0.0
0.5
1.0
0
5
10
15
20
25

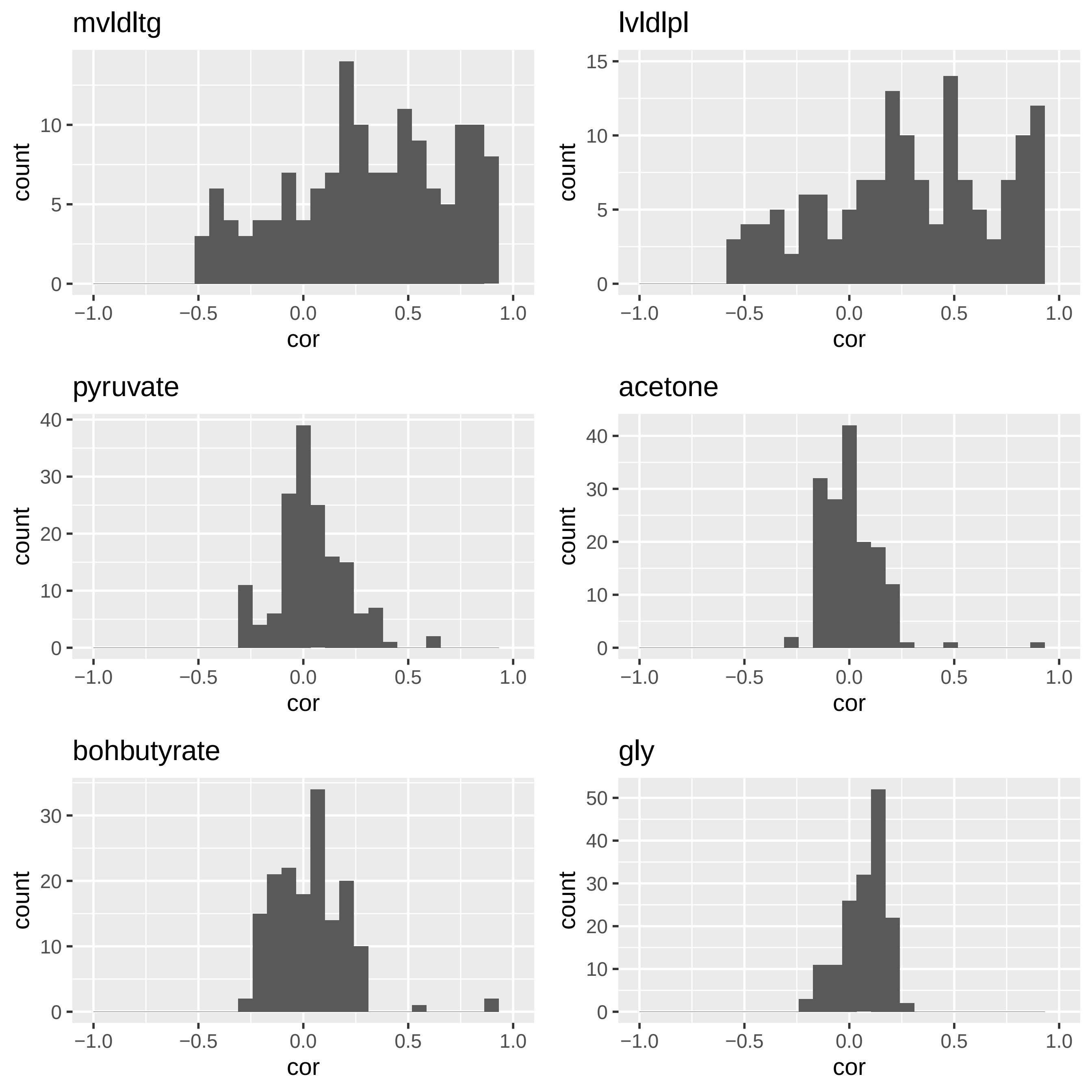
mvldltg
lvldlpl
pyruvate
acetone
bohbutyrate
gly
count
cor
−1.0
−0.5
0.0
0.5
1.0

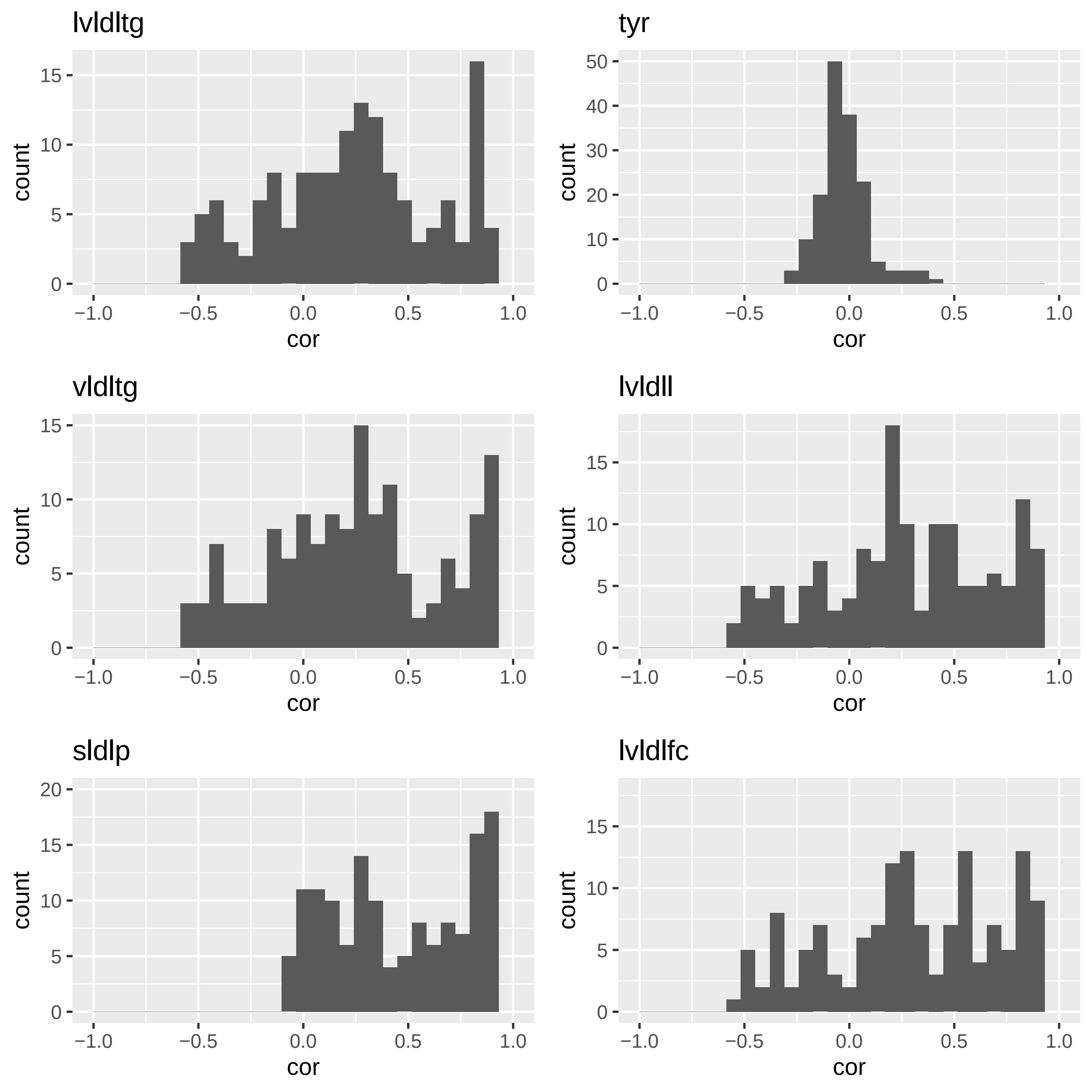

lvldltg
tyr
vldltg
lvldll
sldlp
lvldlfc
count
cor
−1.0
−0.5
0.0
0.5
1.0

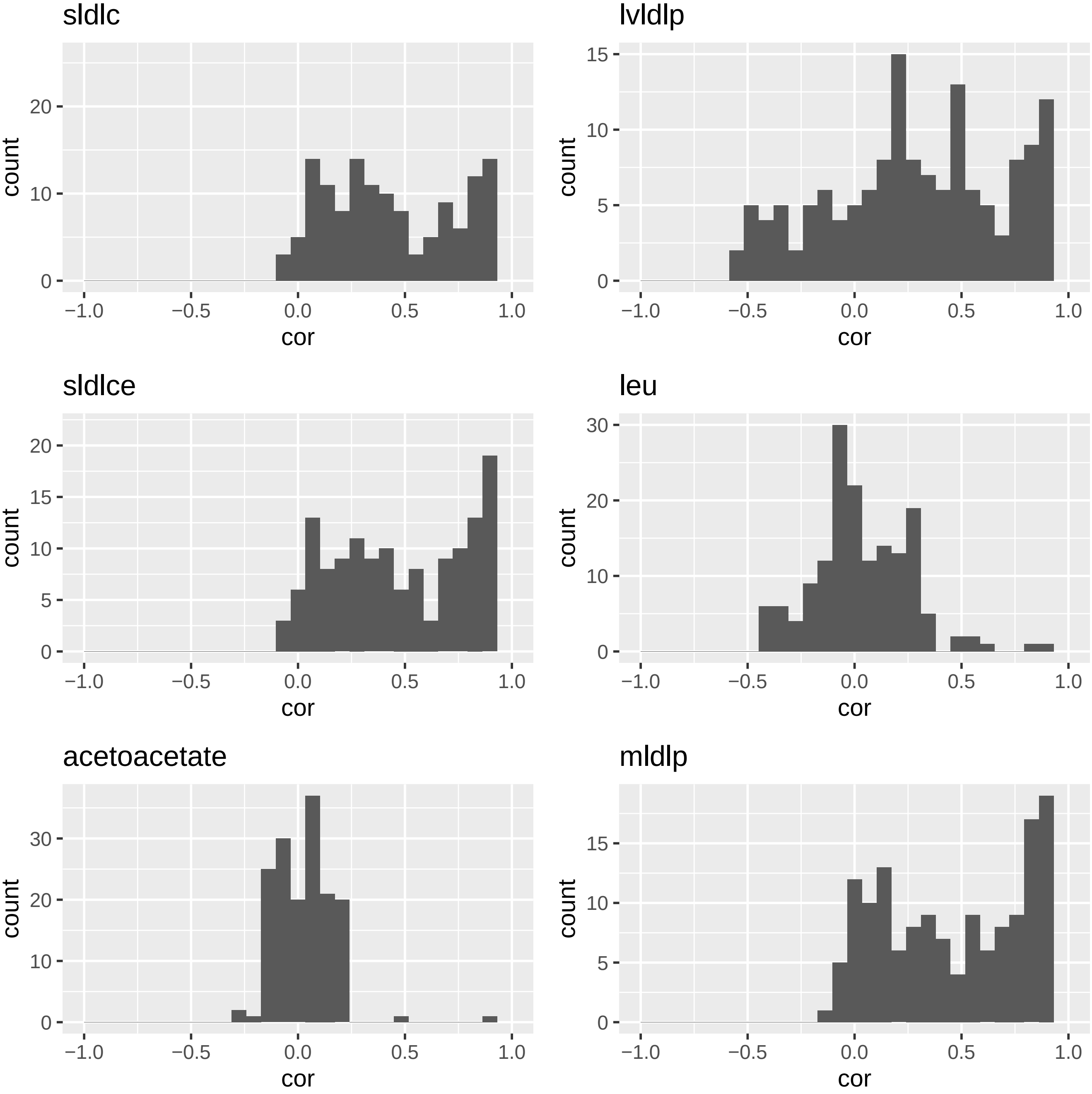
sldlc
lvldlp
sldlce
leu
acetoacetate
mldlp
count
cor
0
5
10
15
20
30
−1.0
−0.5
0.0
0.5
1.0

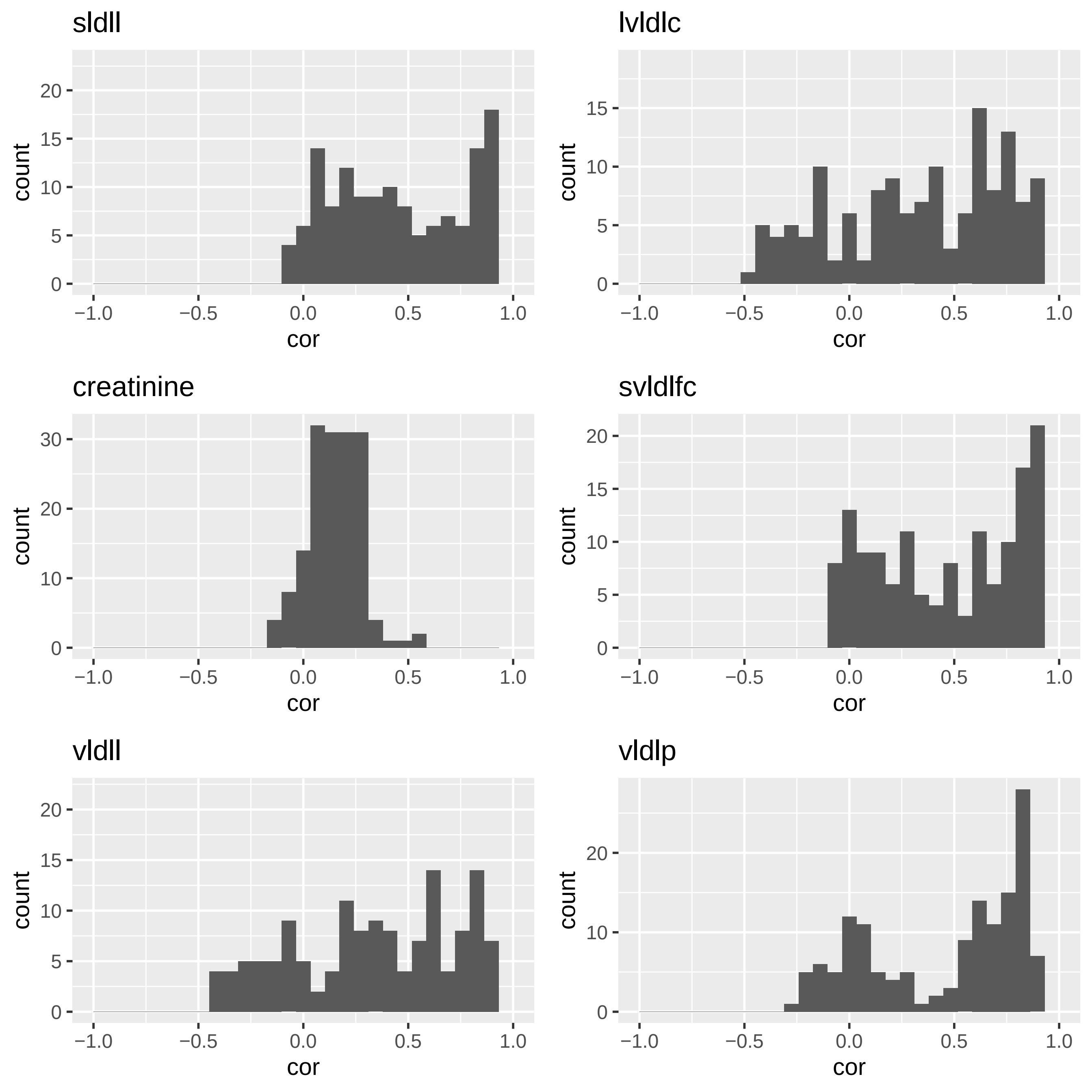

sldll
lvldlc
creatinine
svldlfc
vldll
vldlp
count
cor

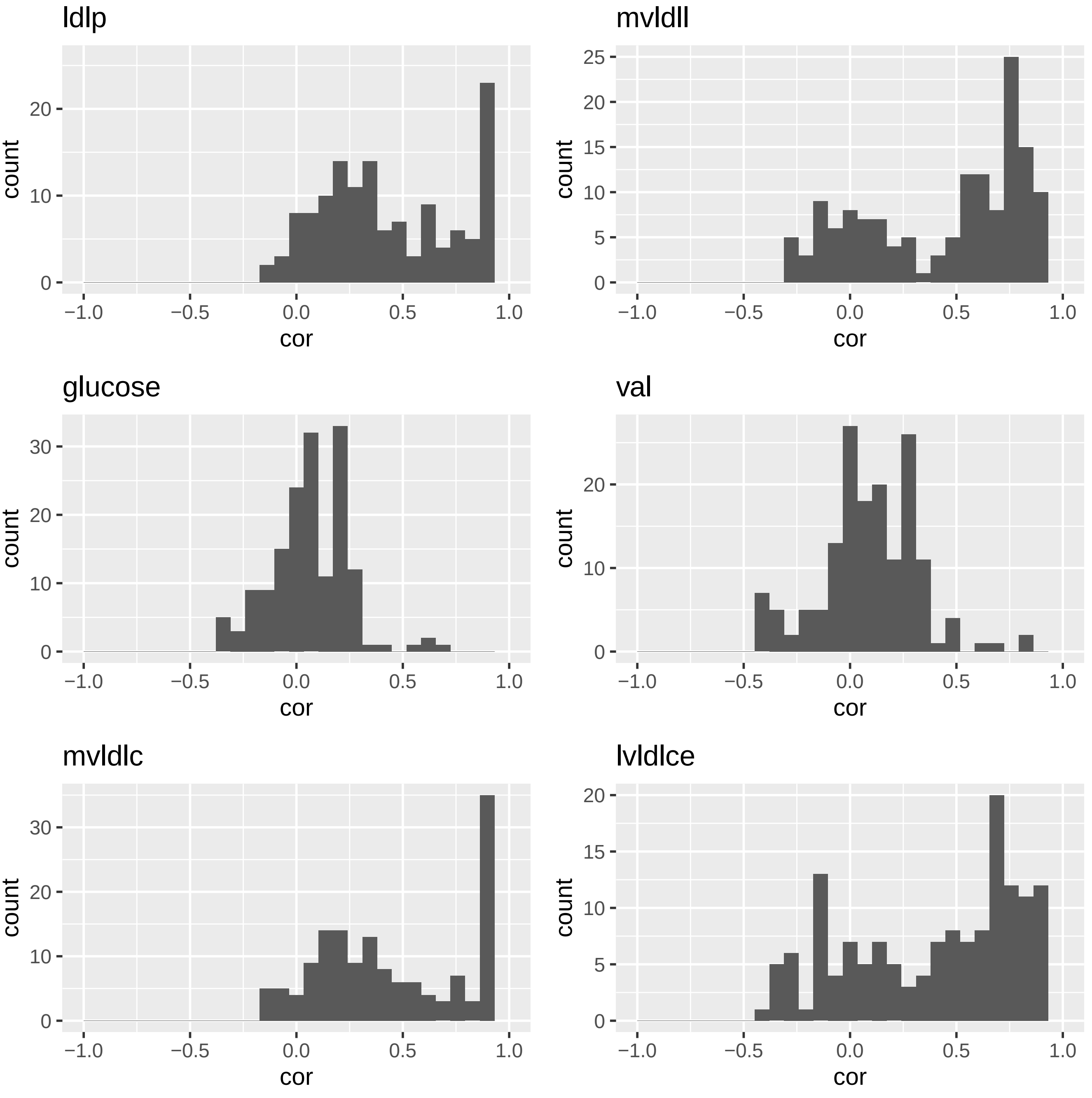

ldlp
mvldll
glucose
val
mvldlc
lvldlce
count
cor
−1.0
−0.5
0.0
0.5
1.0
0
5
10
15
20
25
30

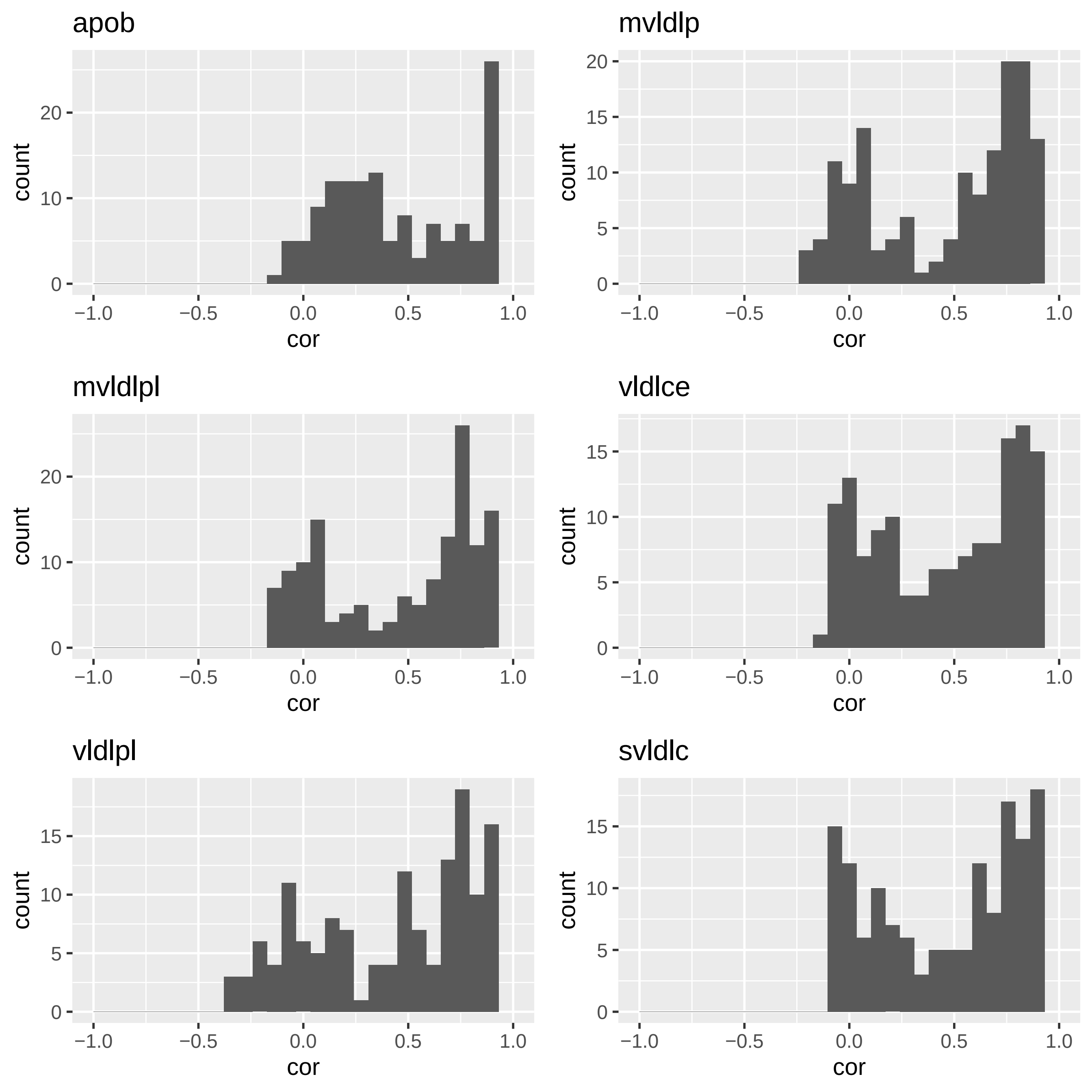
apob
mvldlp
mvldlpl
vldlce
vldlpl
svldlc
count
cor
−1.0
−0.5
0.0
0.5
1.0
0
5
10
15
20

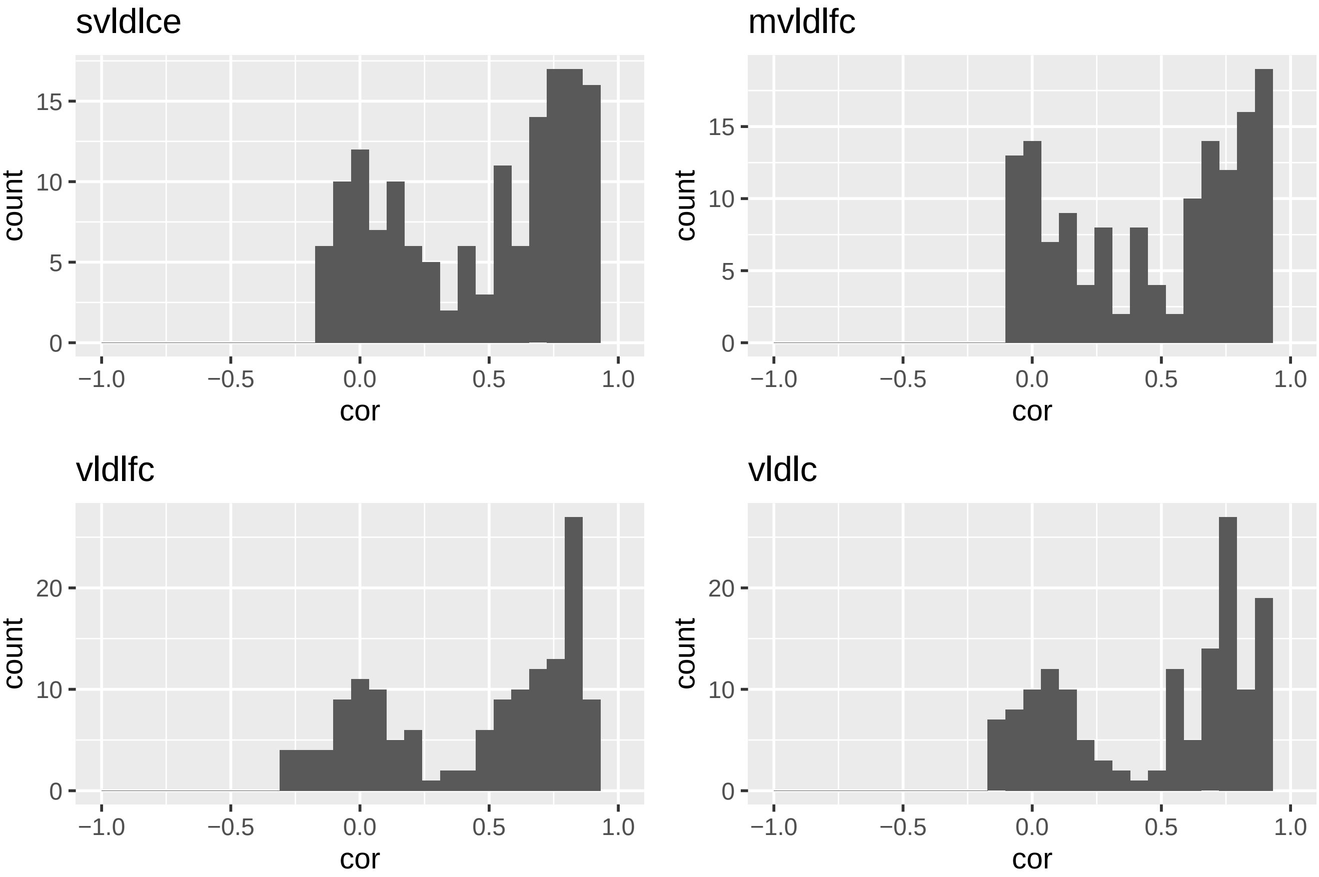
svldlce
mvldlfc
vldlfc
vldlc
count
cor
0
5
10
15
20
−1.0
−0.5
0.0
0.5
1.0
