# Supplementary material for: Multivariate analysis of metabolomic data to identify biological pathways modified by a clinical intervention
Source: Metabolomics. 2026 Jul 27;22(4):134. doi: 10.1007/s11306-026-02490-w (PMC13407567; doi:10.1007/s11306-026-02490-w)
Supplement: Supplementary file 6 — Supplementary Material 6 [file 11306_2026_2490_MOESM6_ESM.pdf]

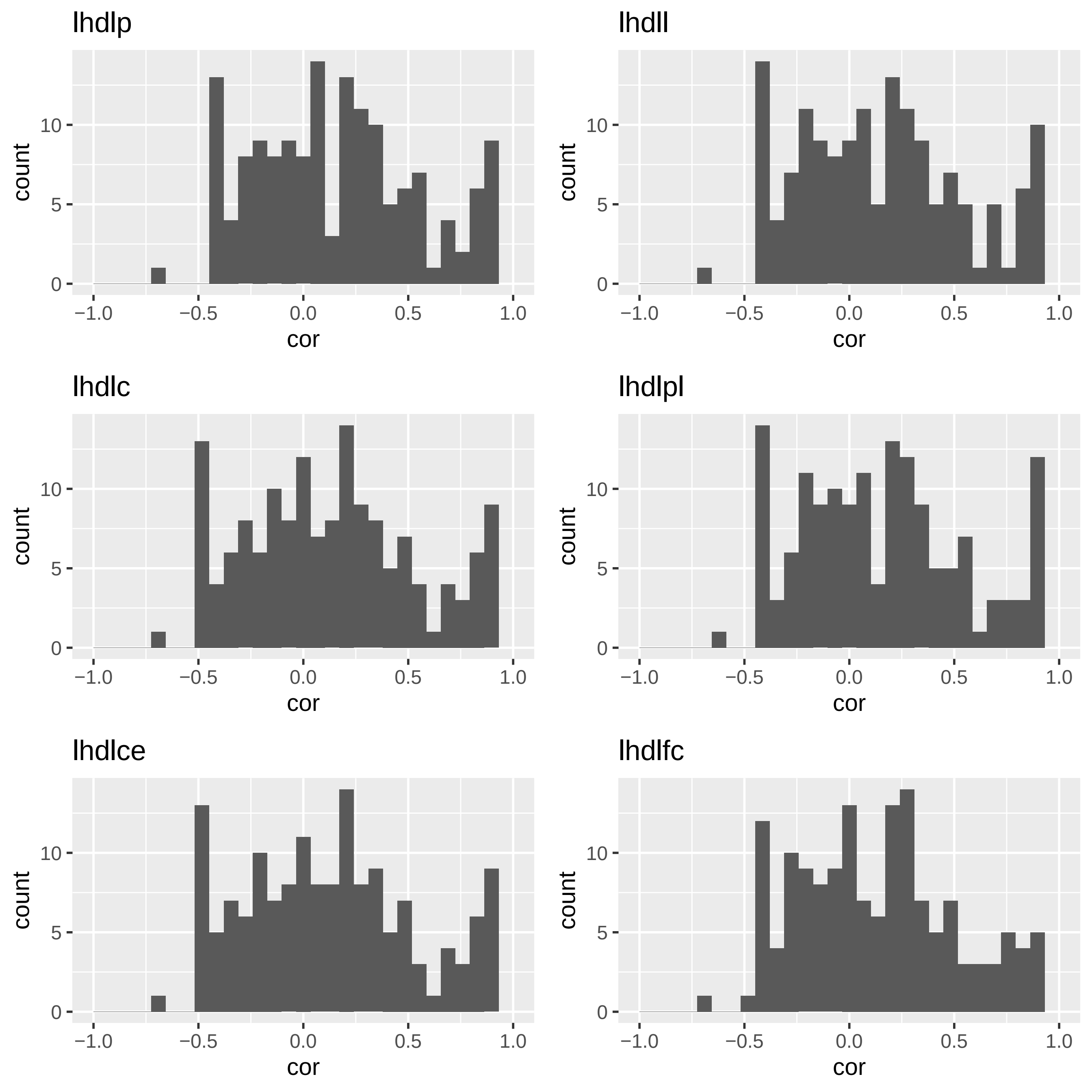
lhdlp
lhdll
lhdlc
lhdlpl
lhdlce
lhdlfc
count
cor
0
5
10
−1.0
−0.5
0.0
0.5
1.0

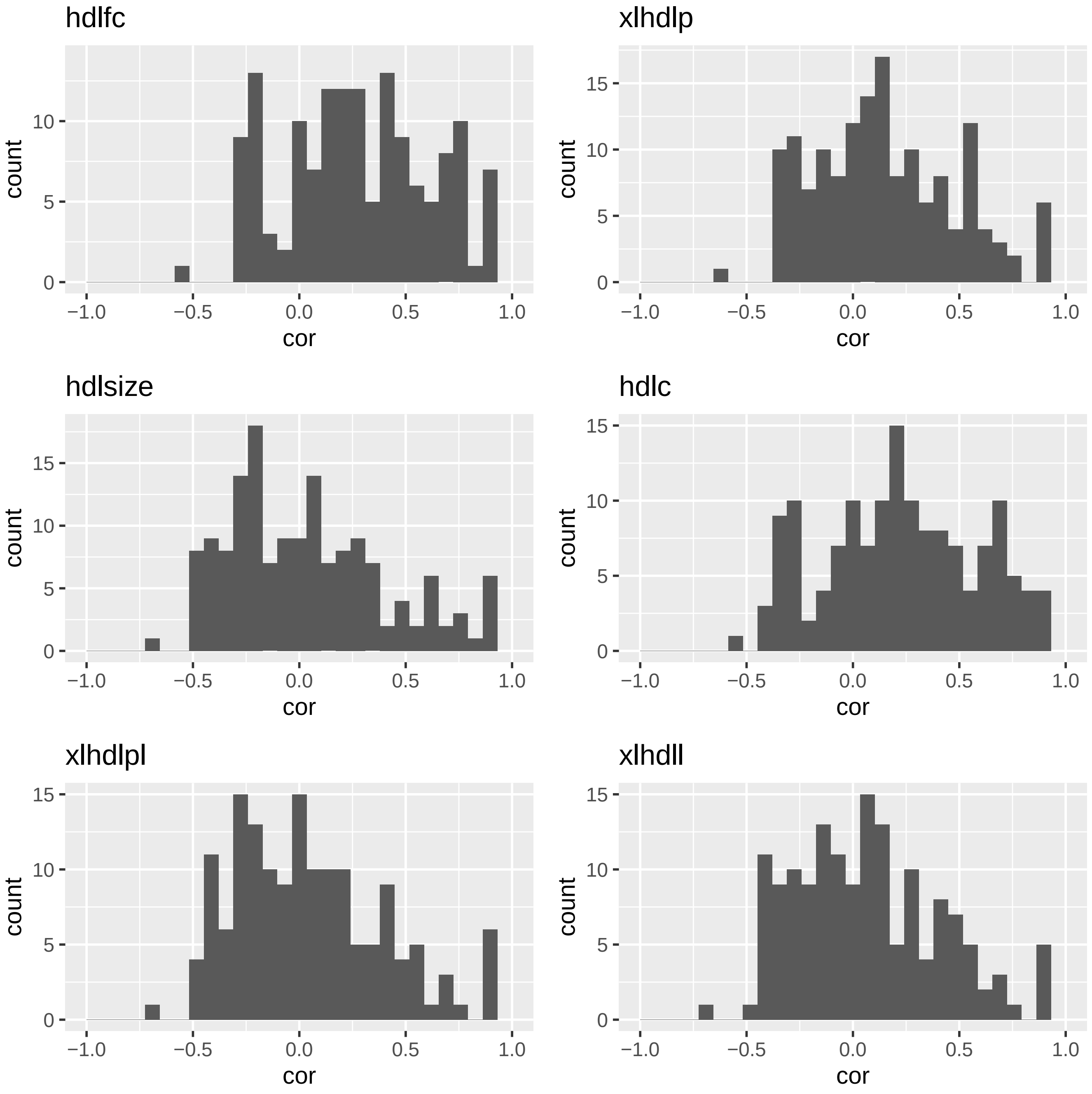
hdlfc
xlhdlp
hdlsize
hdlc
xlhdlpl
xlhdll
count
cor
−1.0
−0.5
0.0
0.5
1.0
0
5
10
15

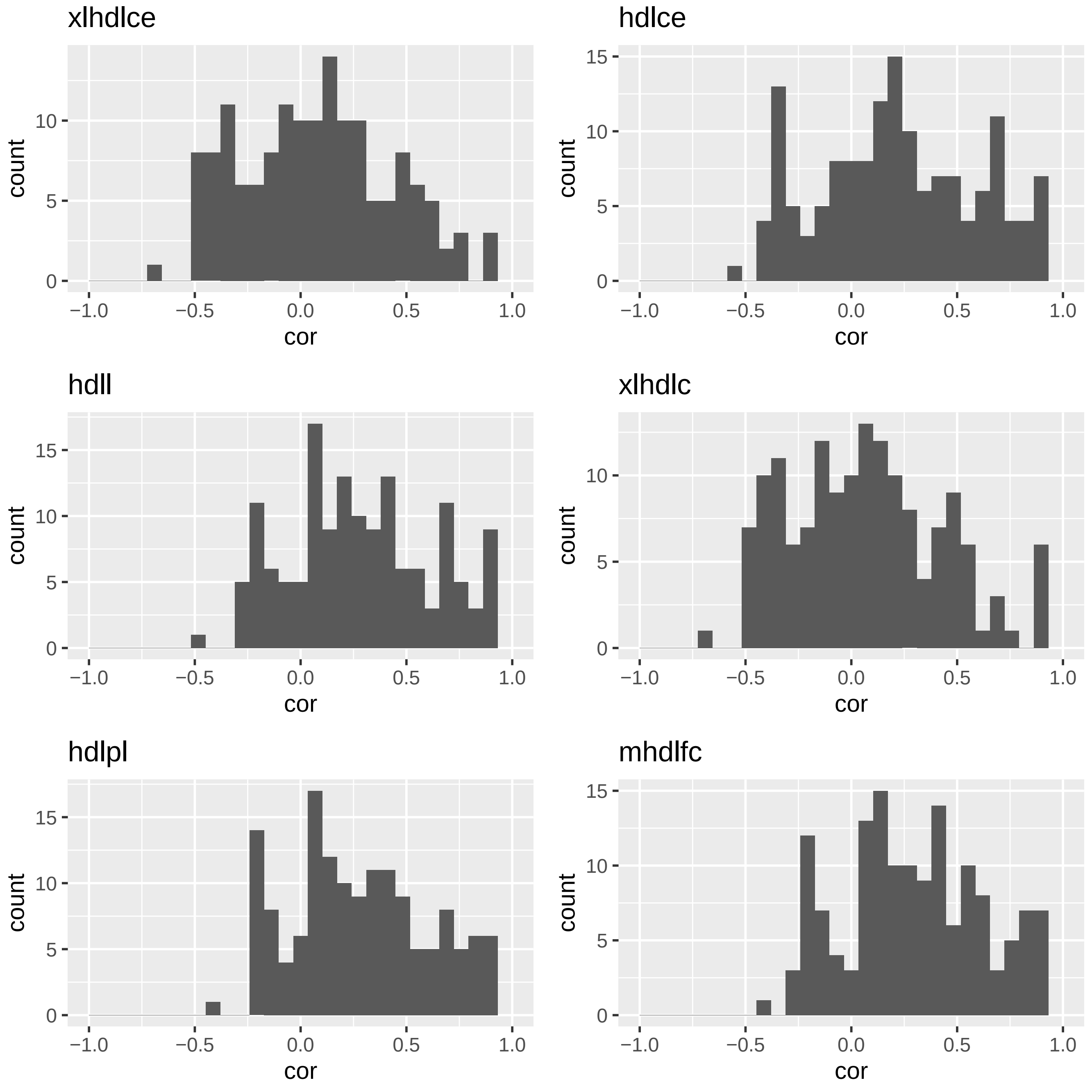
xlhdlce
hdlce
hdll
xlhdlc
hdlpl
mhdlfc
count
cor
0
5
10
15
−1.0
−0.5
0.0
0.5
1.0

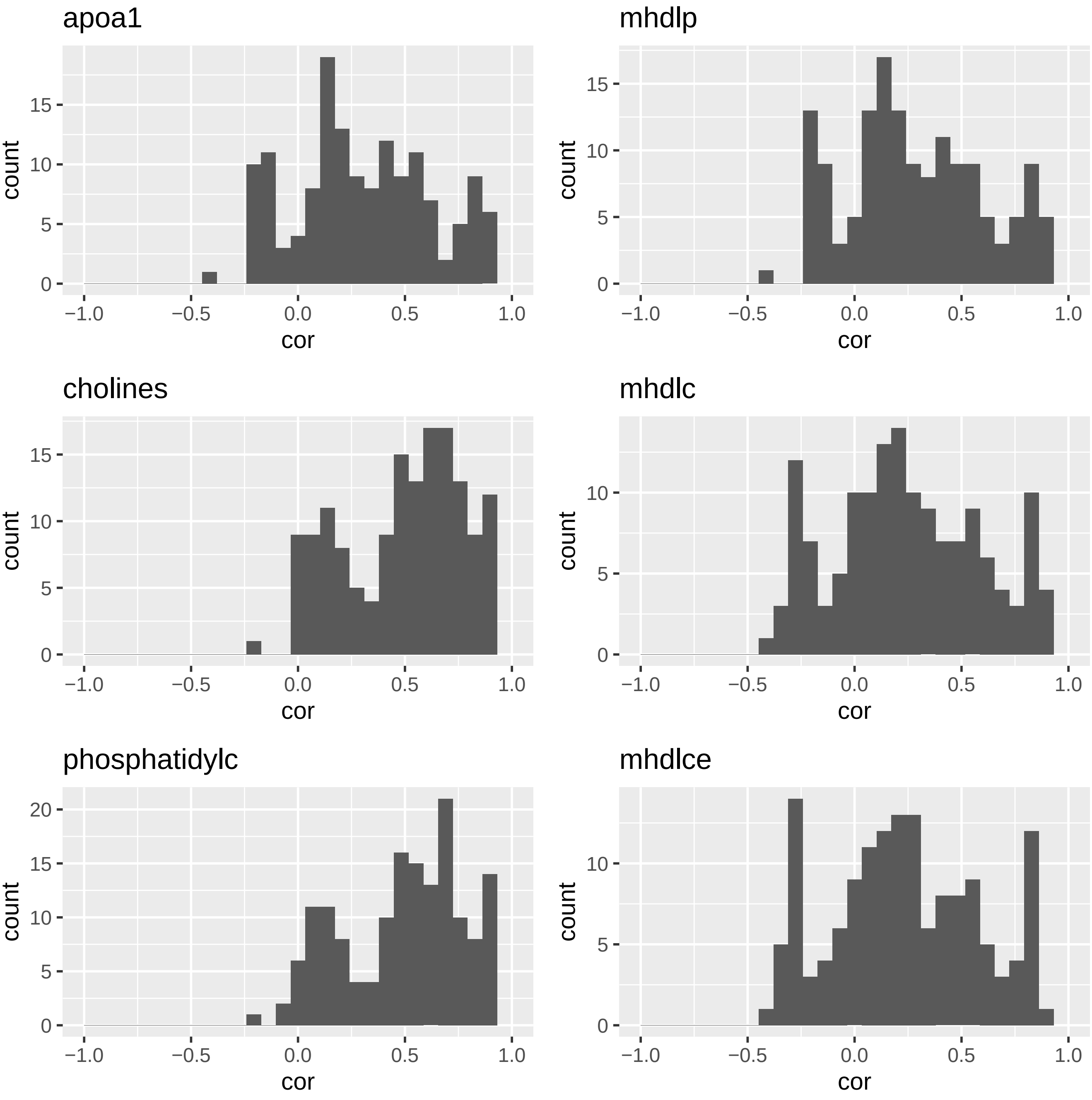
apoa1
mhdlp
cholines
mhdlc
phosphatidylc
mhdlce
count
cor
0
5
10
15
20
−1.0
−0.5
0.0
0.5
1.0

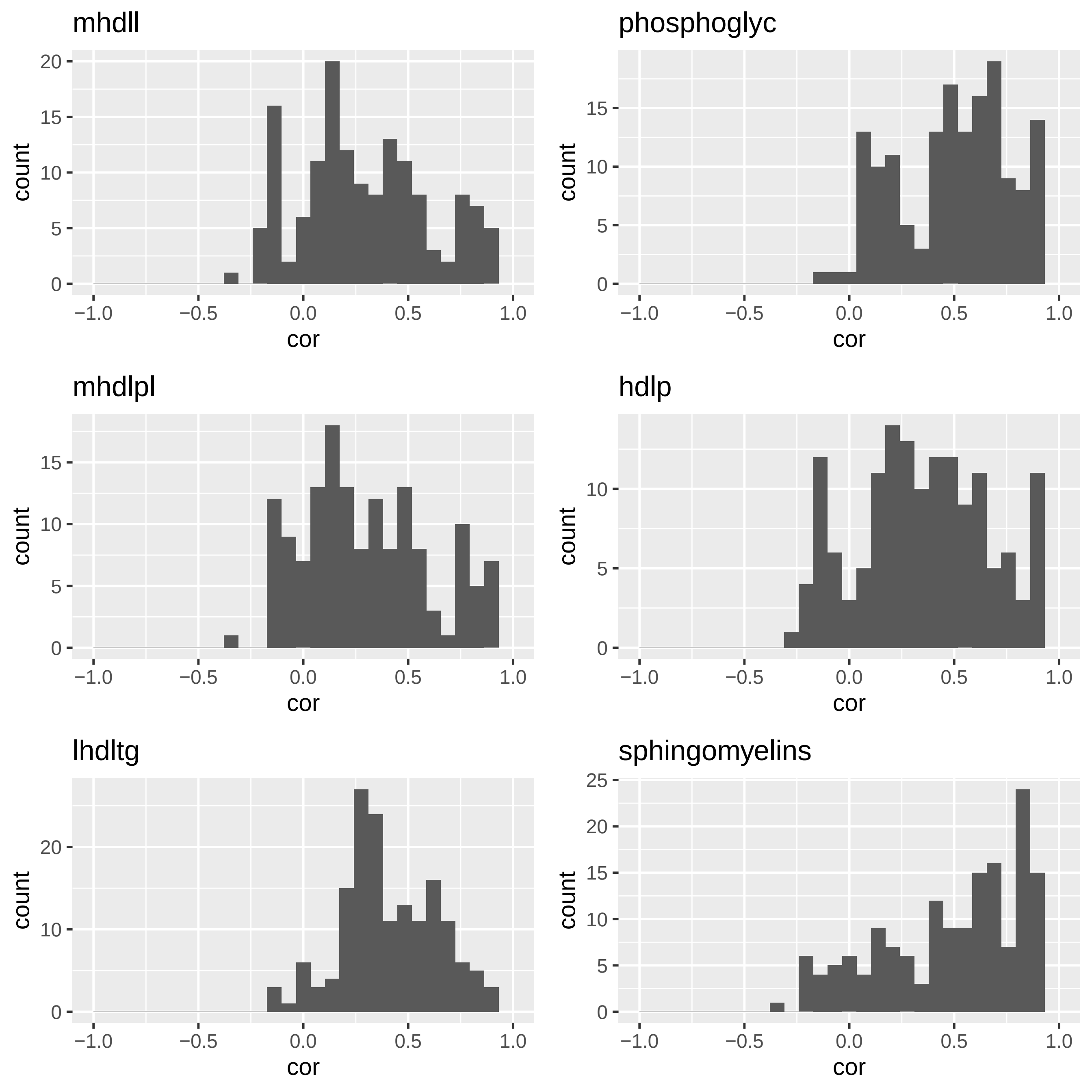
mhdll
phosphoglyc
mhdlpl
hdlp
lhdltg
sphingomyelins
count
cor
−1.0
−0.5
0.0
0.5
1.0
0
5
10
15
20
25

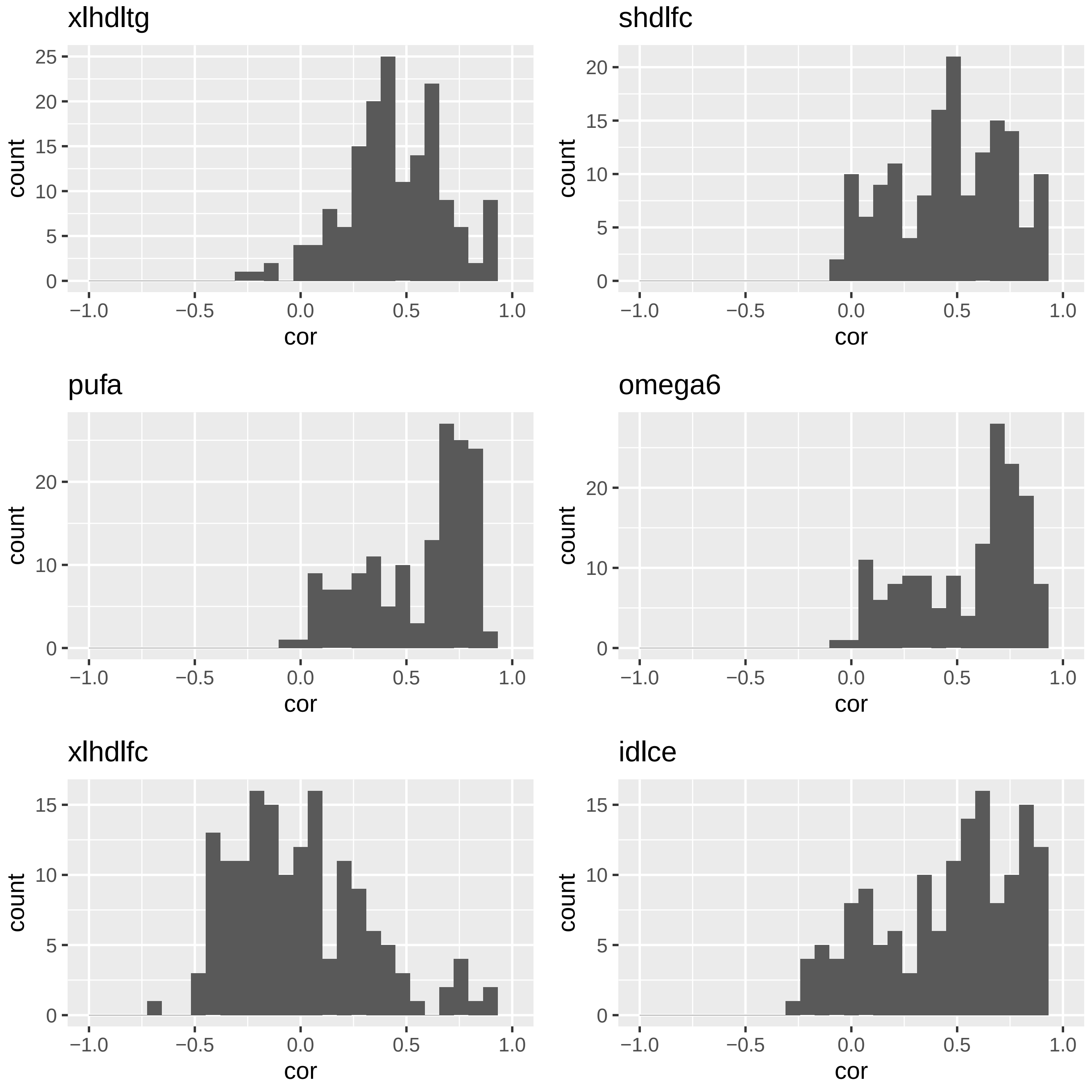

xlhdltg
shdlfc
pufa
omega6
xlhdlfc
idlce
count
cor

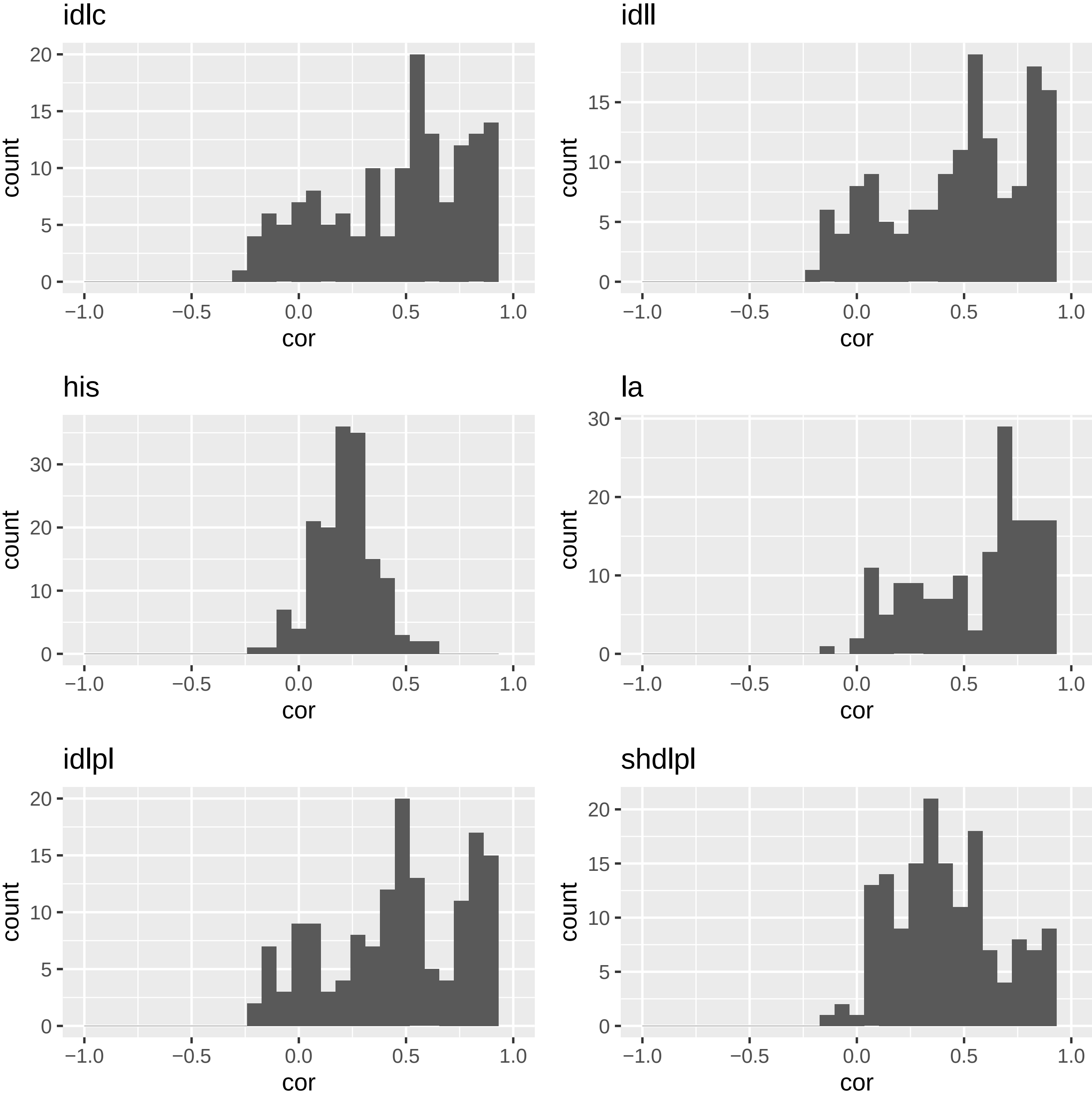
idlc
idll
his
la
idlpl
shdlpl
count
cor
−1.0
−0.5
0.0
0.5
1.0
0
5
10
15
20
30

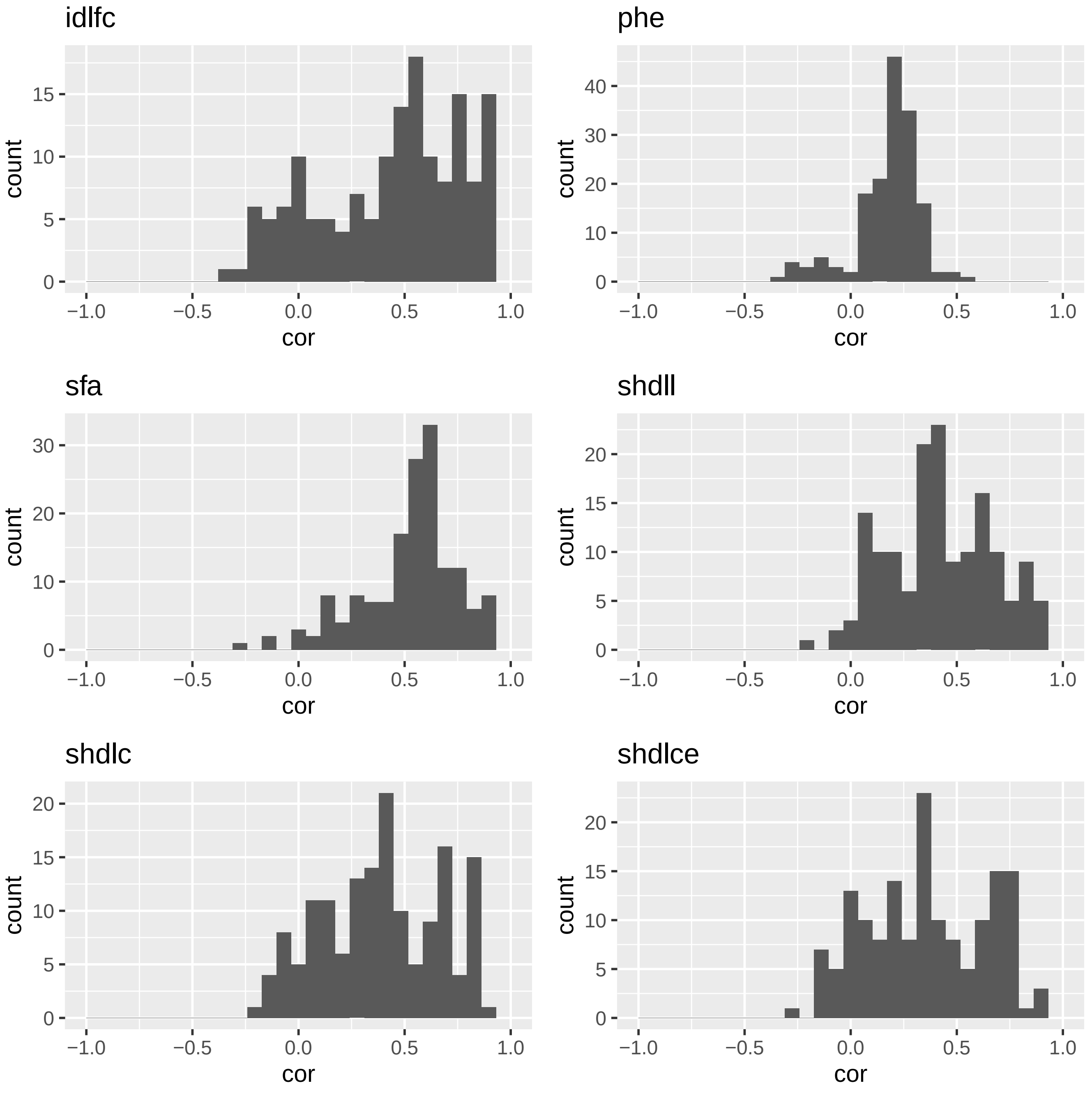
idlfc
phe
sfa
shdll
shdlc
shdlce
count
cor
0
5
10
15
20
30
40
−1.0
−0.5
0.0
0.5
1.0

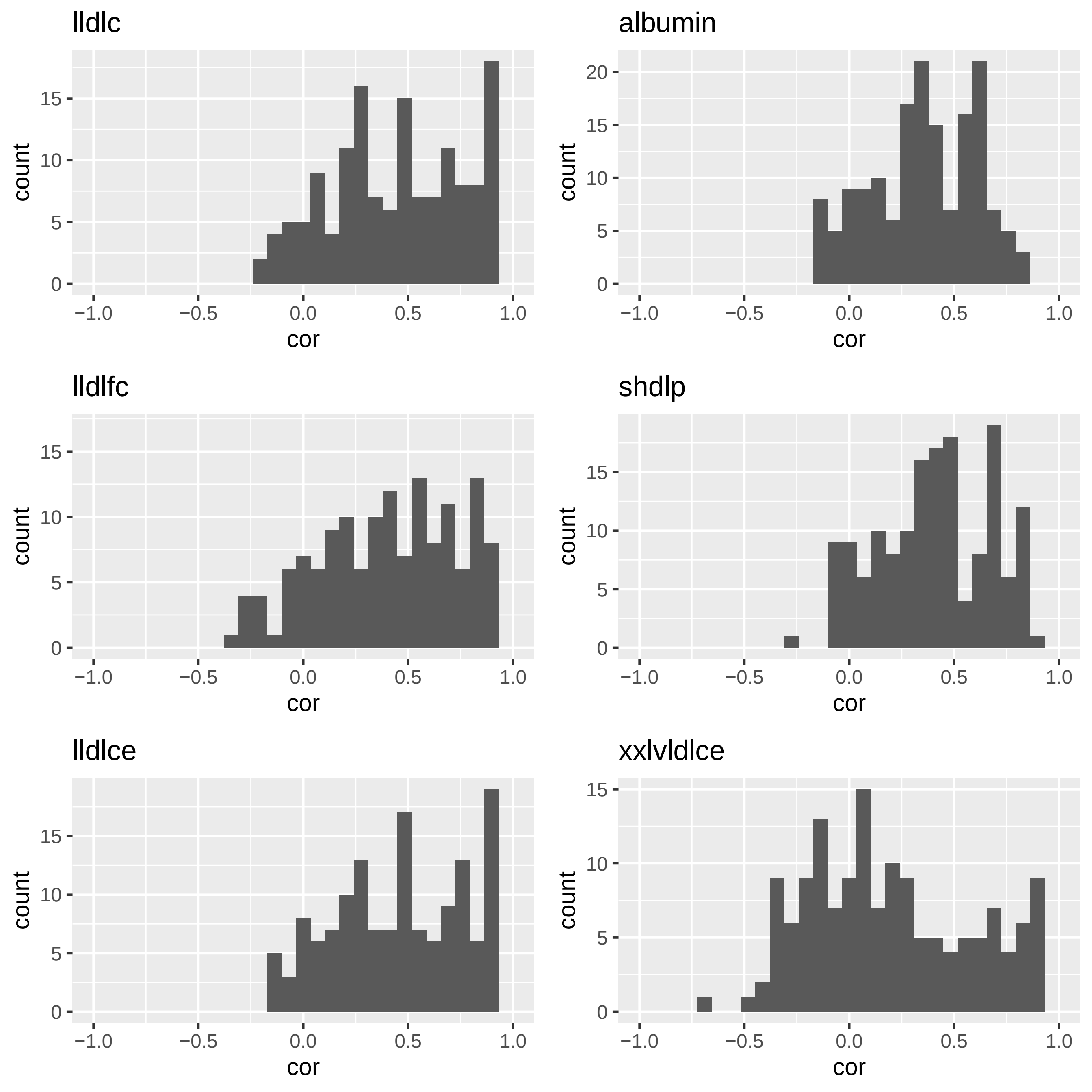
lldlc
albumin
lldlfc
shdlp
lldlce
xxlvldlce
count
cor
−1.0
−0.5
0.0
0.5
1.0
0
5
10
15
20

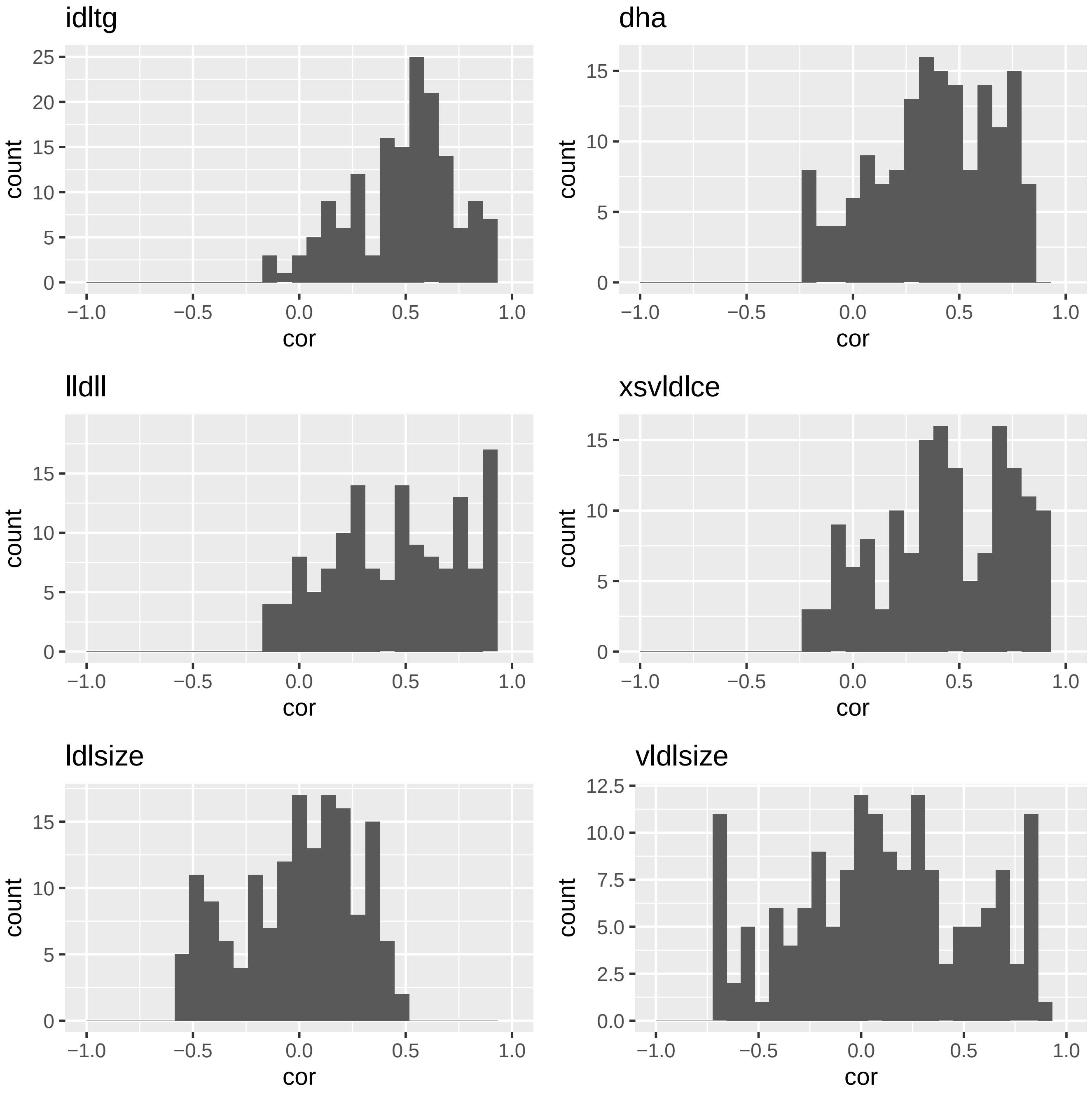
idltg
dha
lldll
xsvldlce
ldlsize
vldlsize
count
cor
25
20
15
10
5
0
12.5
10.0
7.5
5.0
2.5
0.0
−1.0
−0.5
0.0
0.5
1.0

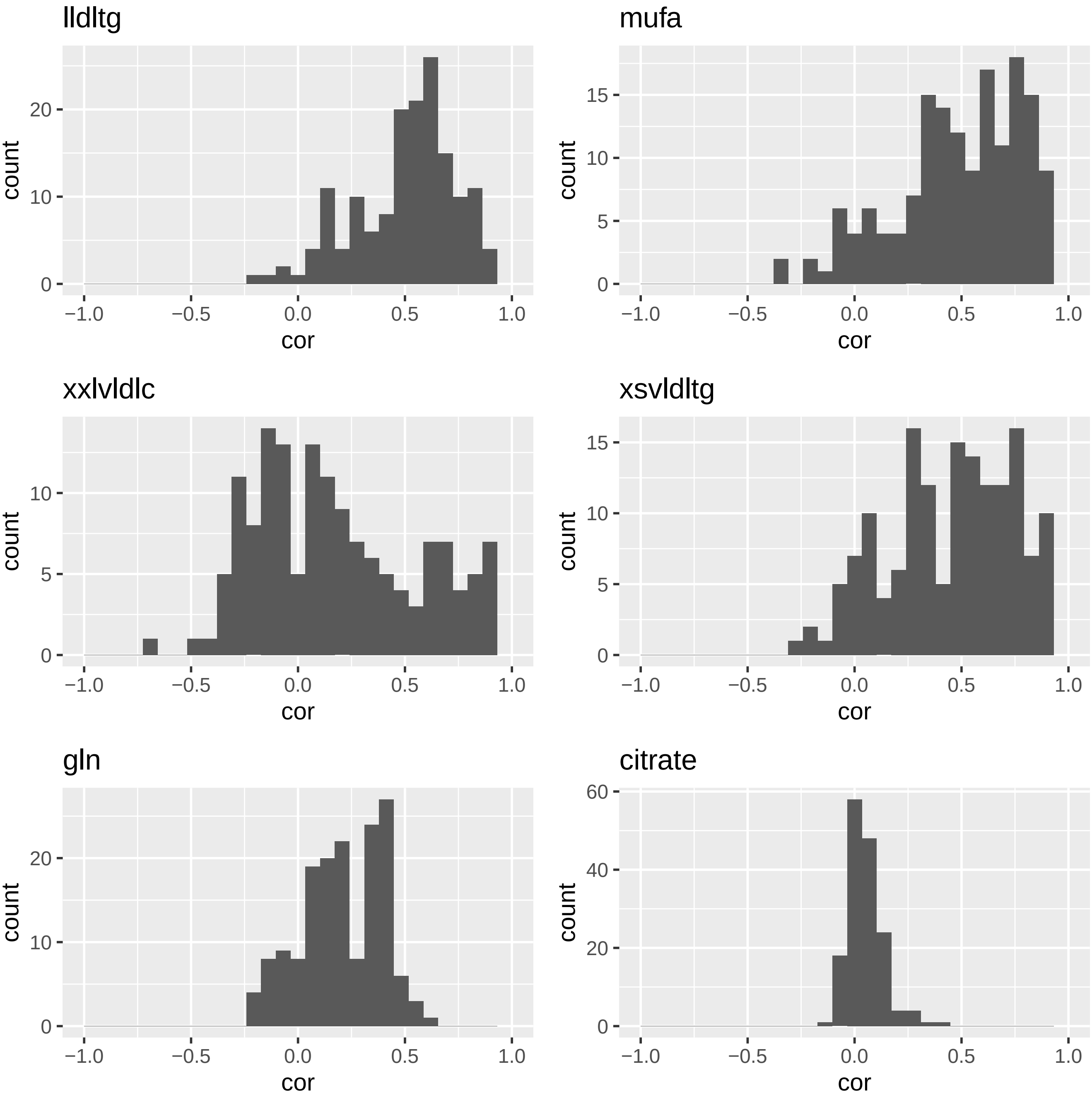
lldltg
mufa
xxlvldlc
xsvldltg
gln
citrate
count
cor
0
10
20
5
15
40
60
−1.0
−0.5
0.0
0.5
1.0

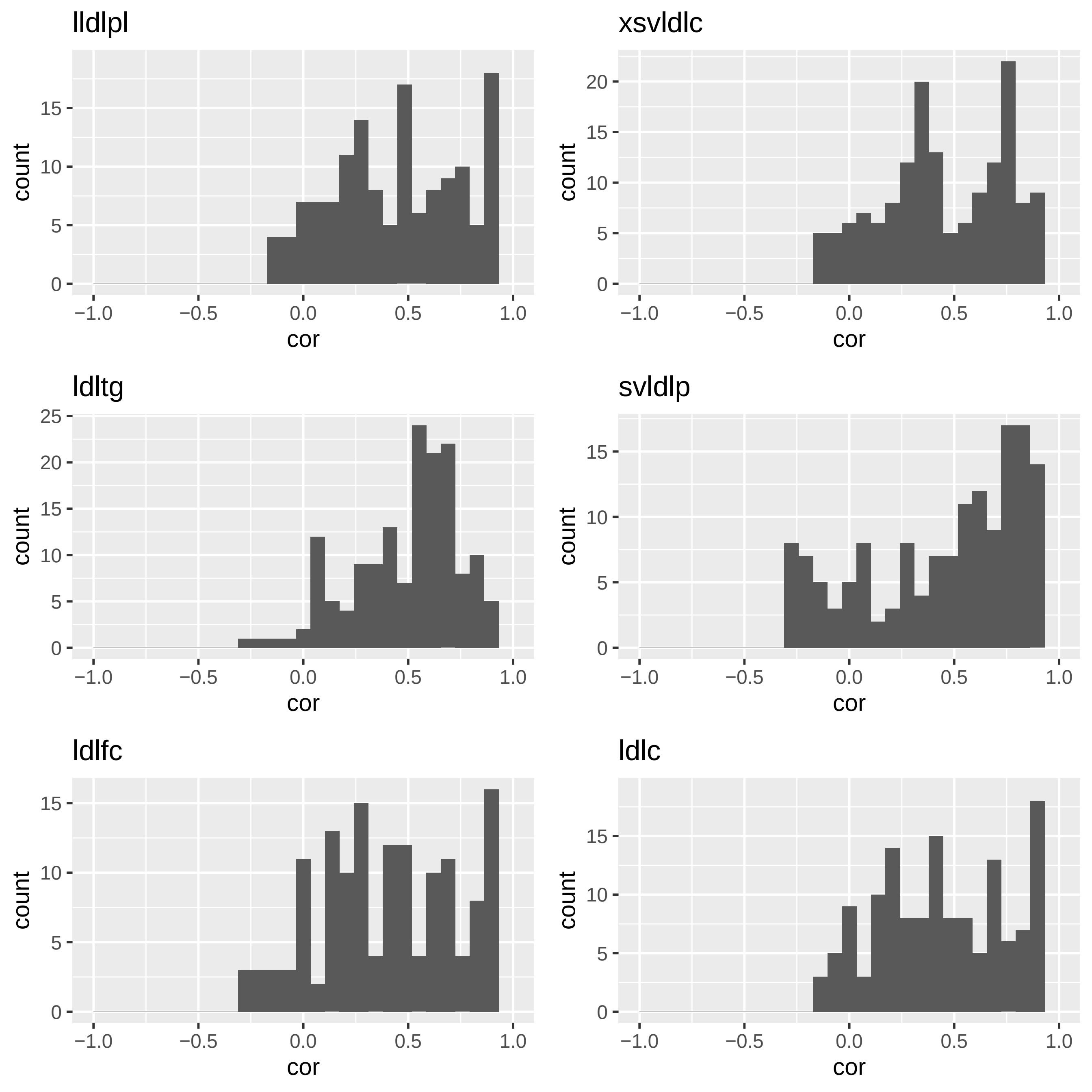

lldlpl
xsvldlc
ldltg
svldlp
ldlfc
ldlc
count
cor
−1.0
−0.5
0.0
0.5
1.0

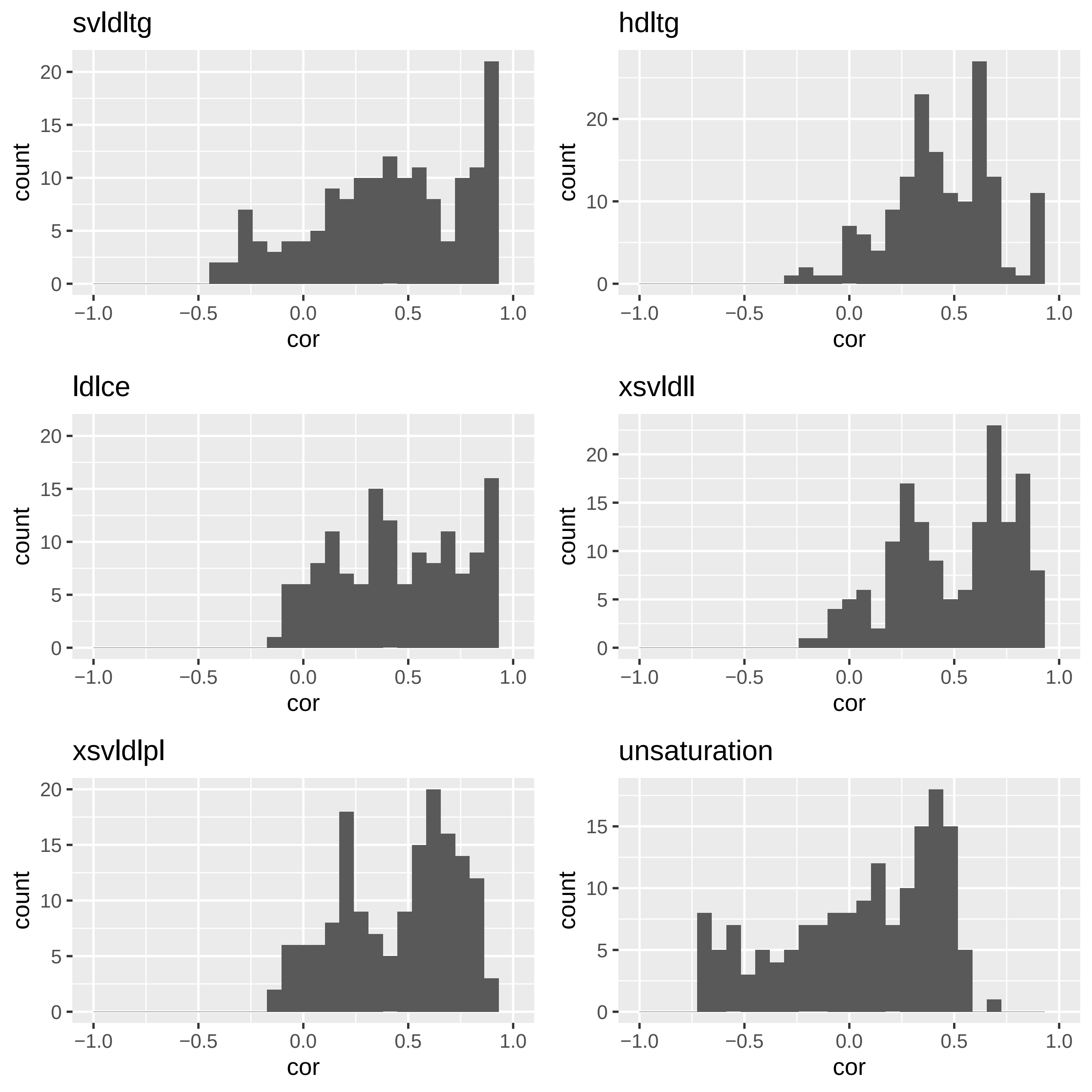

svldltg
hdltg
ldlce
xsvldll
xsvldlpl
unsaturation
count
cor

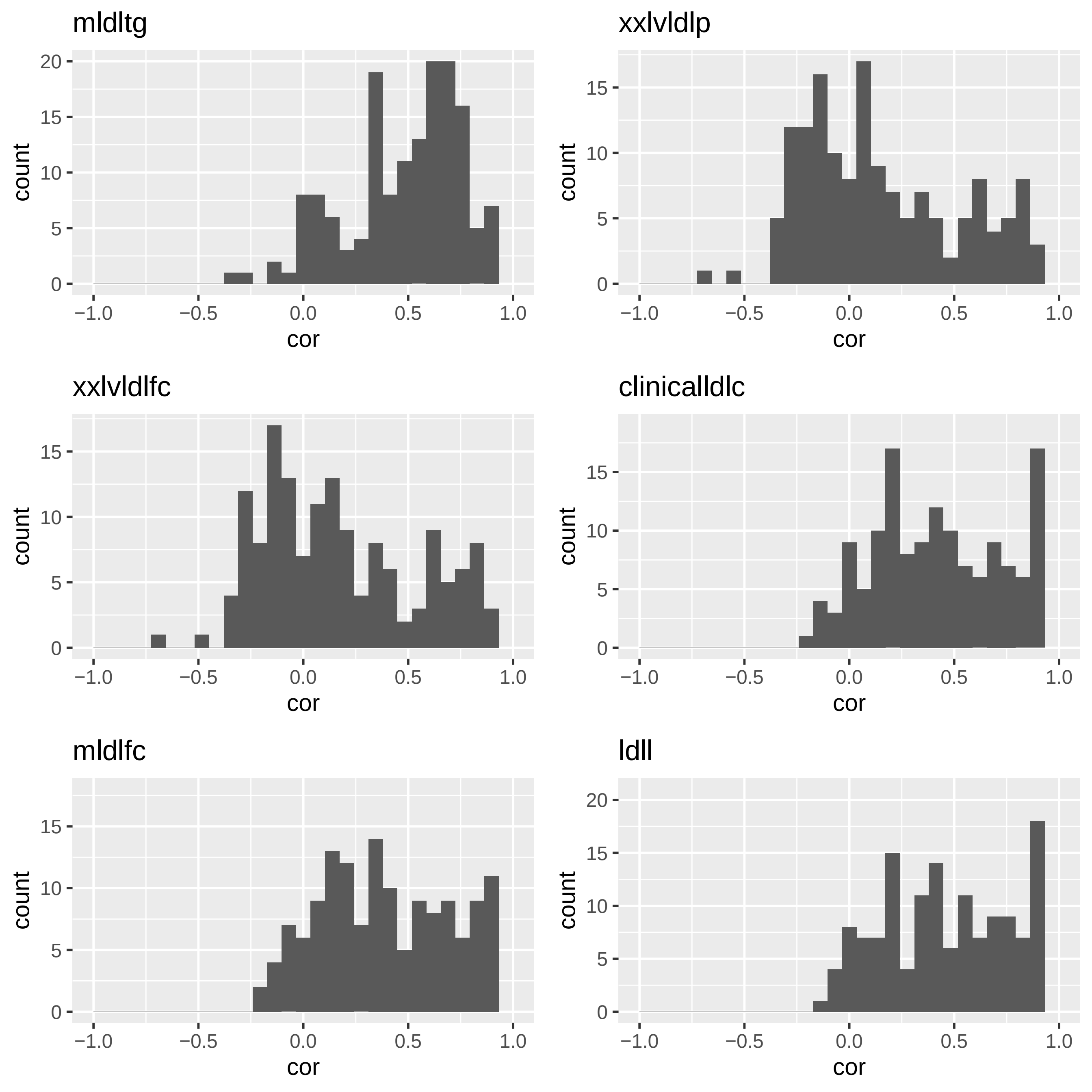
mldltg
xxlvldlp
xxlvldlfc
clinicalldlc
mldlfc
ldll
count
cor
0
5
10
15
20
−1.0
−0.5
0.0
0.5
1.0

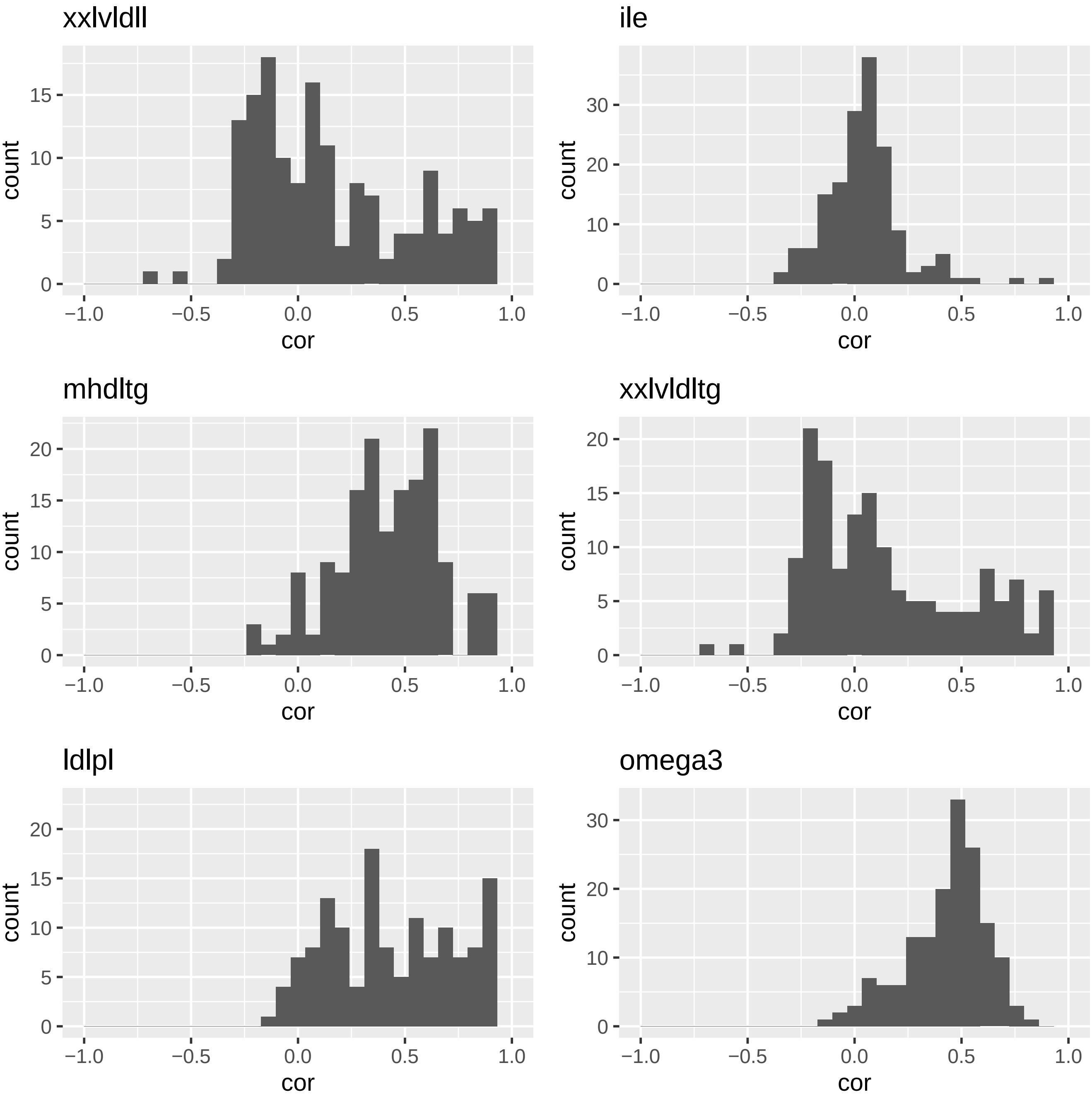

xxlvldll
count
0
5
10
15
−1.0
−0.5
0.0
0.5
1.0
cor
ile
count
0
10
20
30
−1.0
−0.5
0.0
0.5
1.0
cor
mhdltg
count
0
5
10
15
20
−1.0
−0.5
0.0
0.5
1.0
cor
xxlvldltg
count
0
5
10
15
20
−1.0
−0.5
0.0
0.5
1.0
cor
ldlpl
count
0
5
10
15
20
−1.0
−0.5
0.0
0.5
1.0
cor
omega3
count
0
10
20
30
−1.0
−0.5
0.0
0.5
1.0
cor

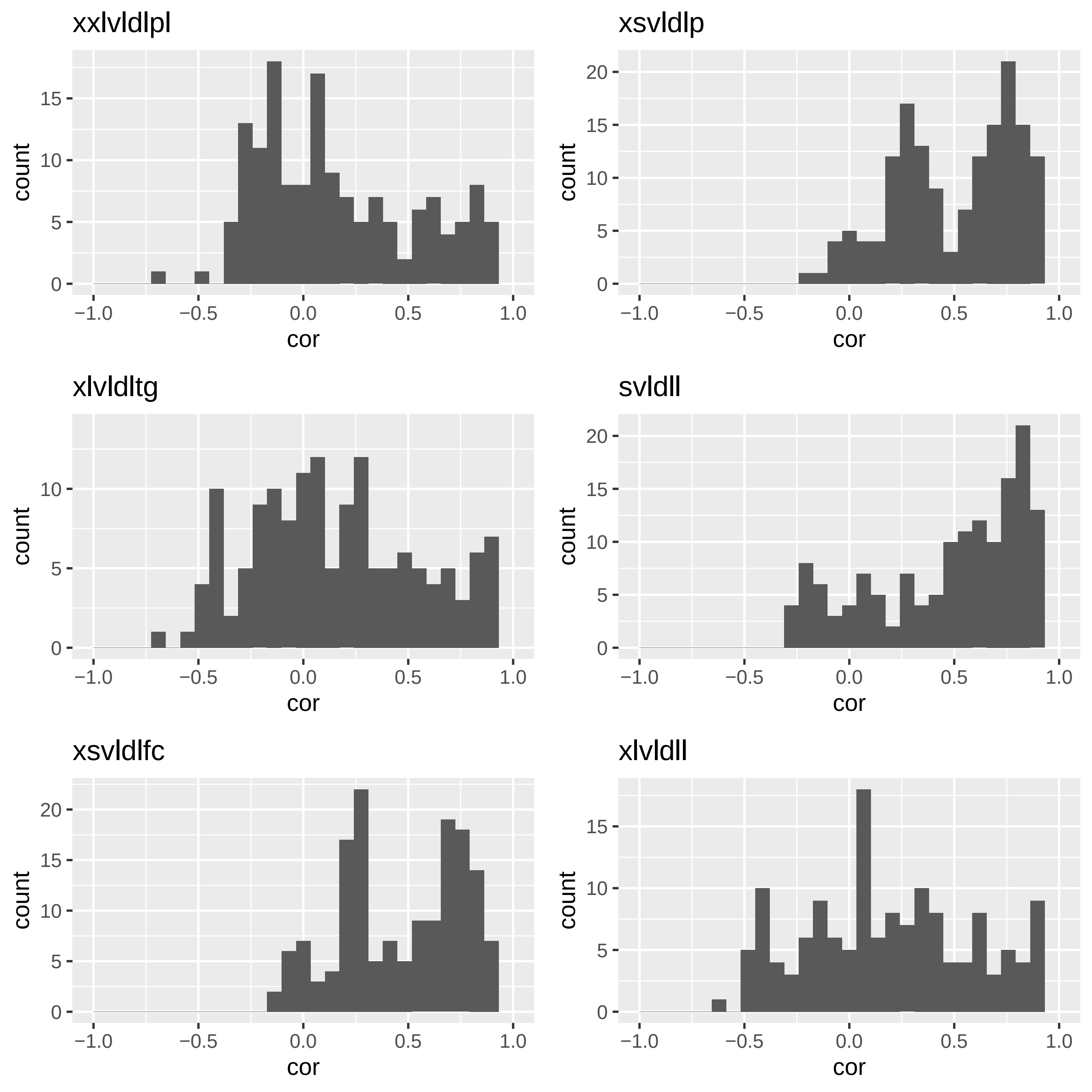
xxlvldlpl
xsvldlp
xlvldltg
svldll
xsvldlfc
xlvldll
count
cor
−1.0
−0.5
0.0
0.5
1.0
0
5
10
15
20

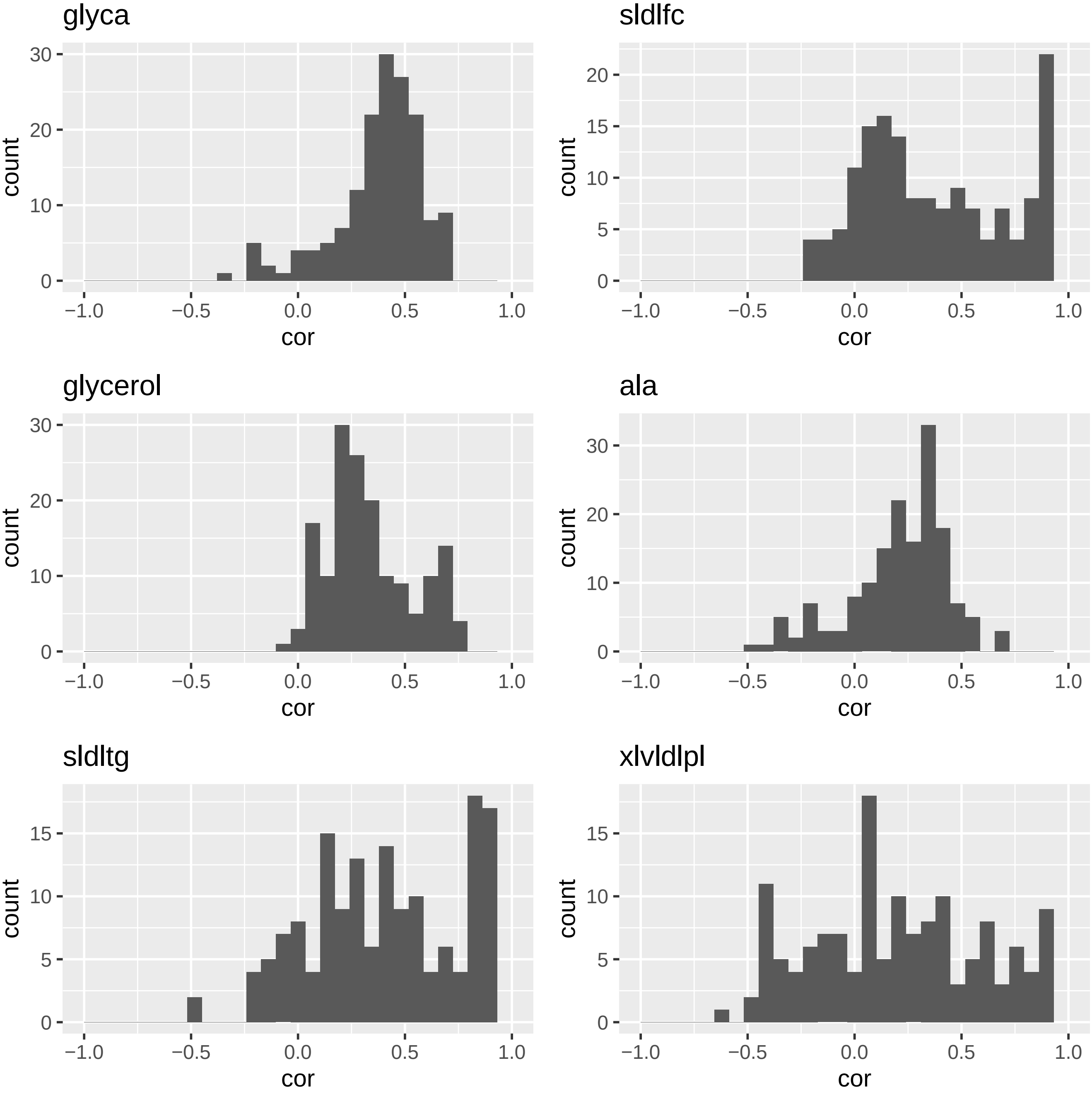
glyca
sldlfc
glycerol
ala
sldltg
xlvldlpl
count
cor
−1.0
−0.5
0.0
0.5
1.0
0
5
10
15
20
30

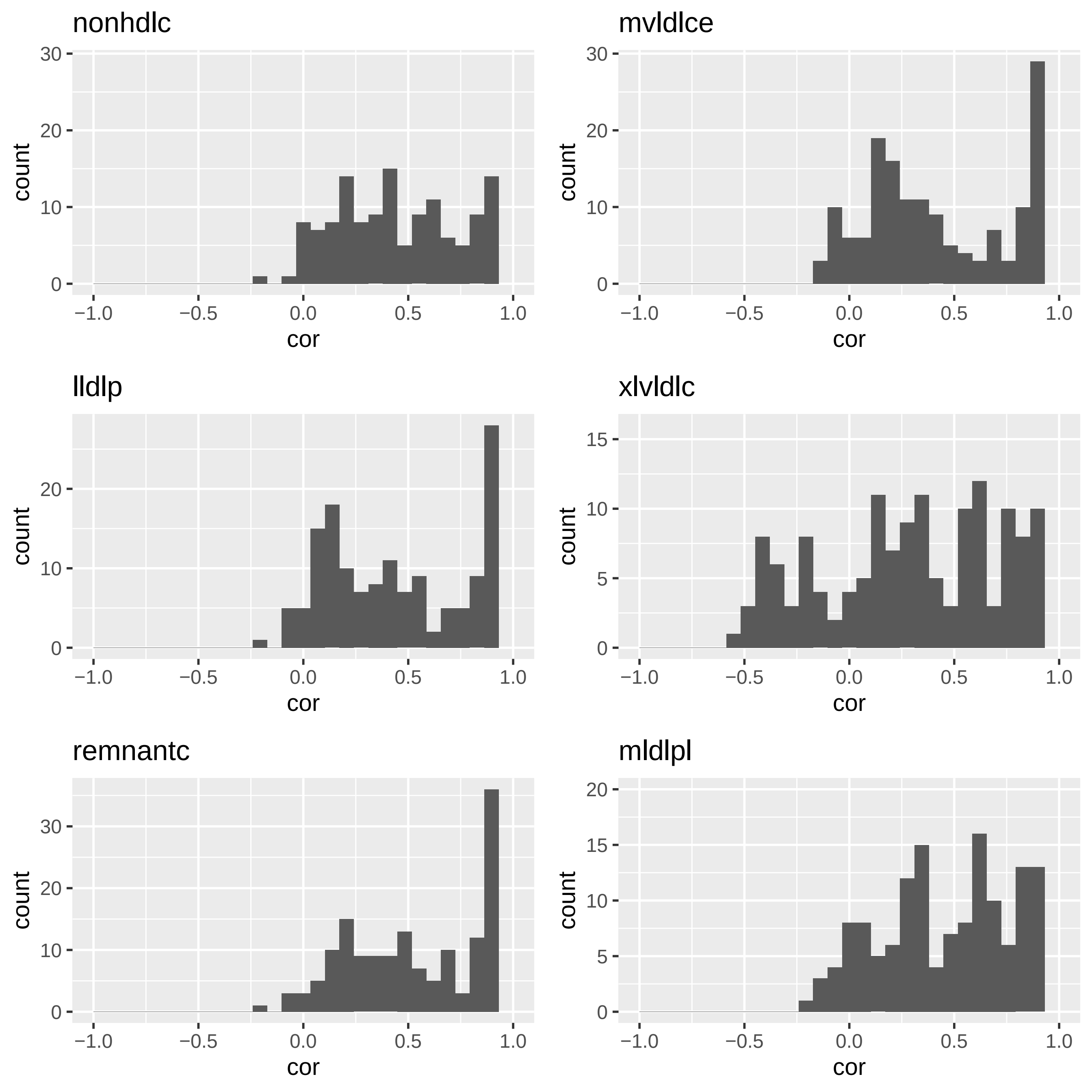

nonhdlc
mvldlce
lldlp
xlvldlc
remnantc
mldlpl
count
cor

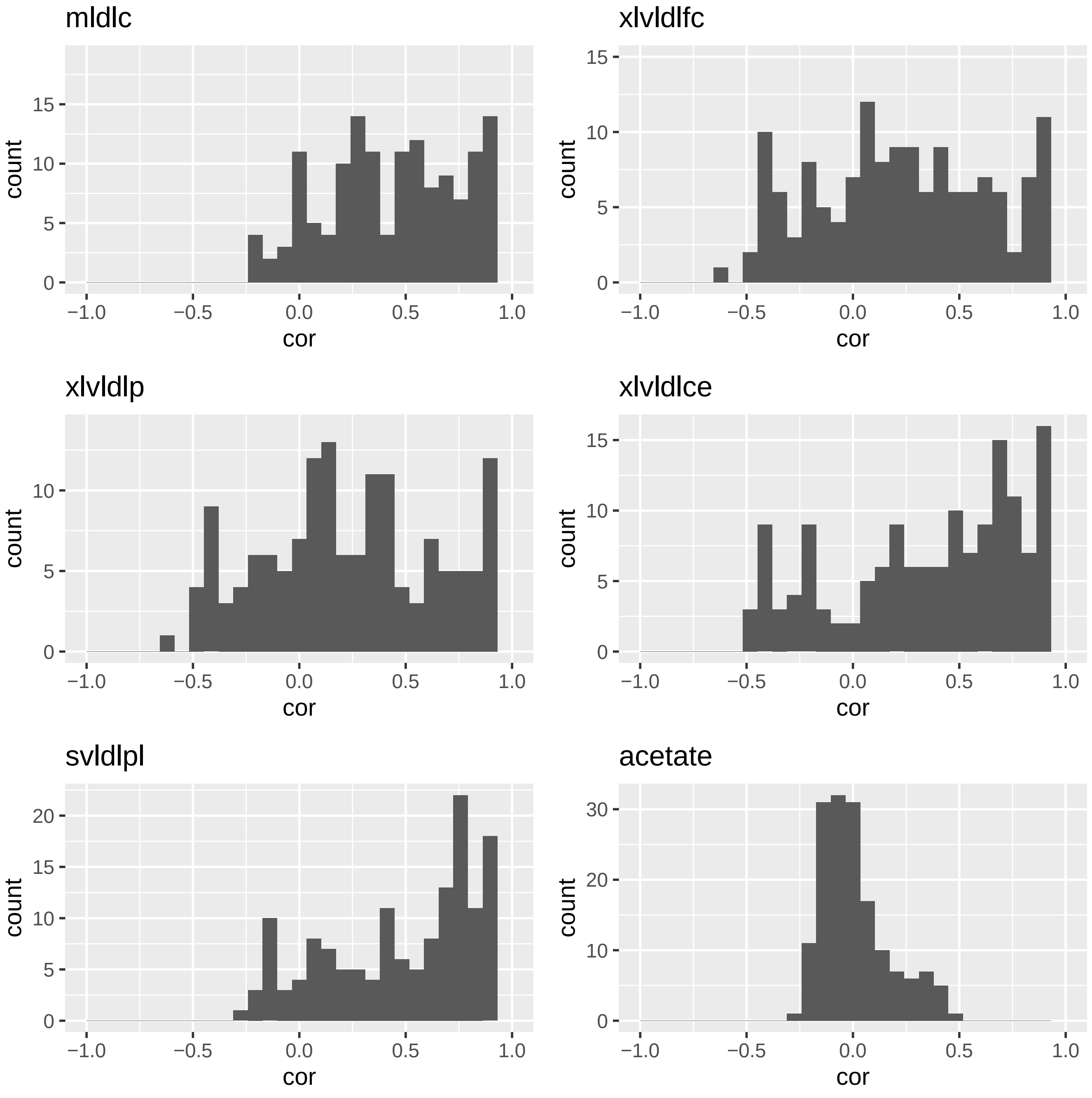
mldlc
xlvldlfc
xlvldlp
xlvldlce
svldlpl
acetate
count
cor
−1.0
−0.5
0.0
0.5
1.0
0
5
10
15
20
30

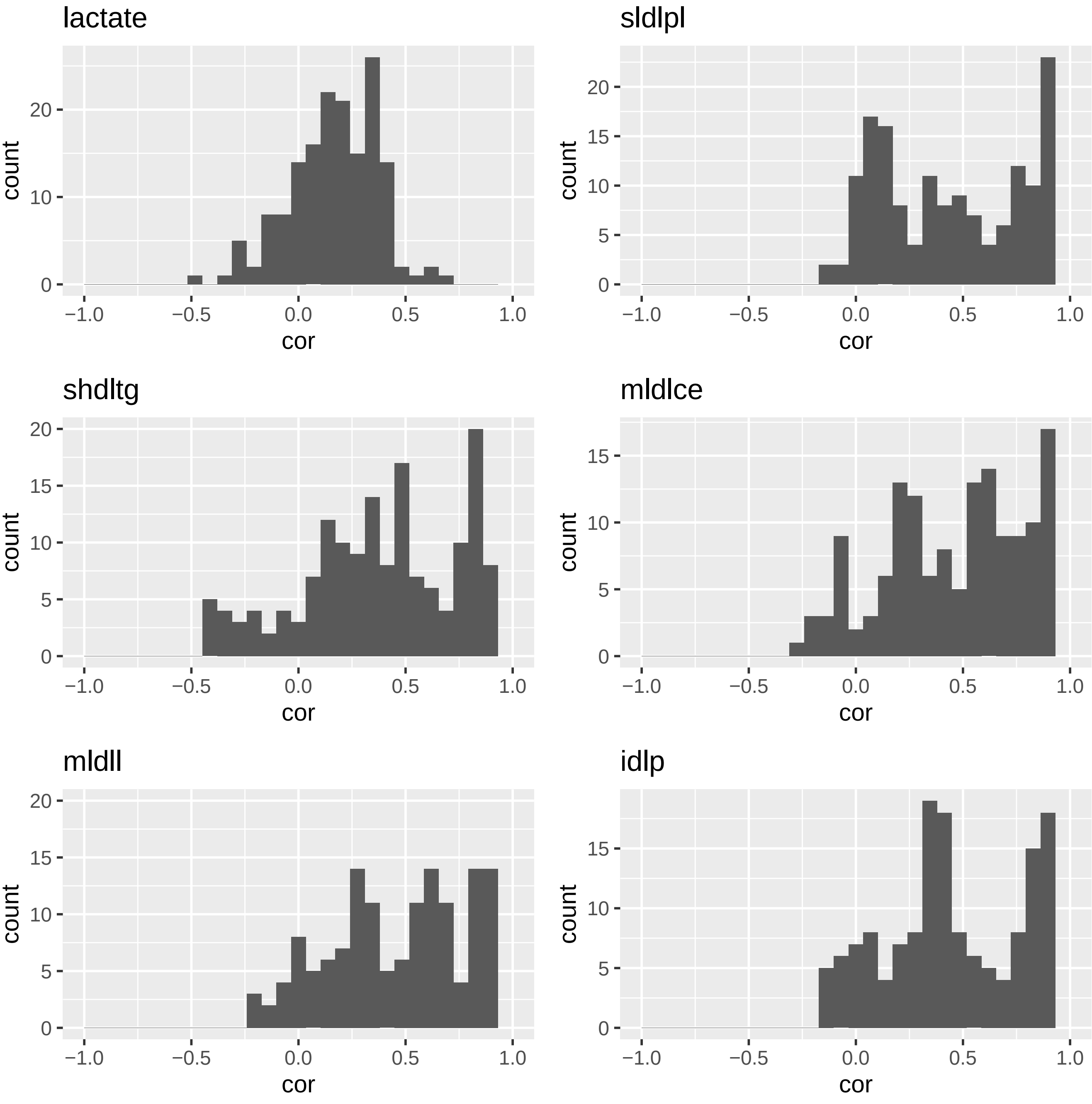
lactate
sldlpl
shdltg
mldlce
mldll
idlp
count
cor
−1.0
−0.5
0.0
0.5
1.0
0
5
10
15
20

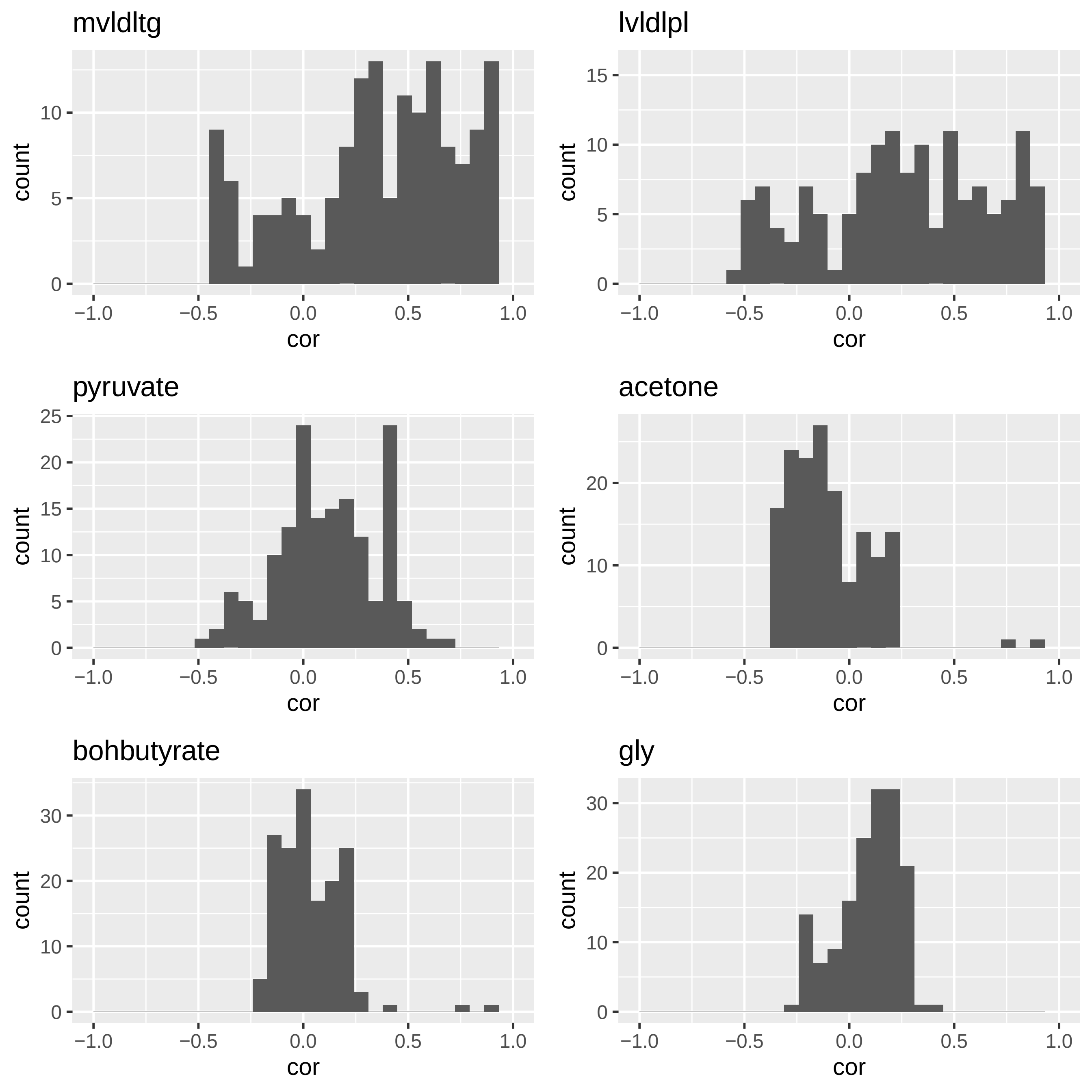
mvldltg
lvldlpl
pyruvate
acetone
bohbutyrate
gly
count
cor
−1.0
−0.5
0.0
0.5
1.0

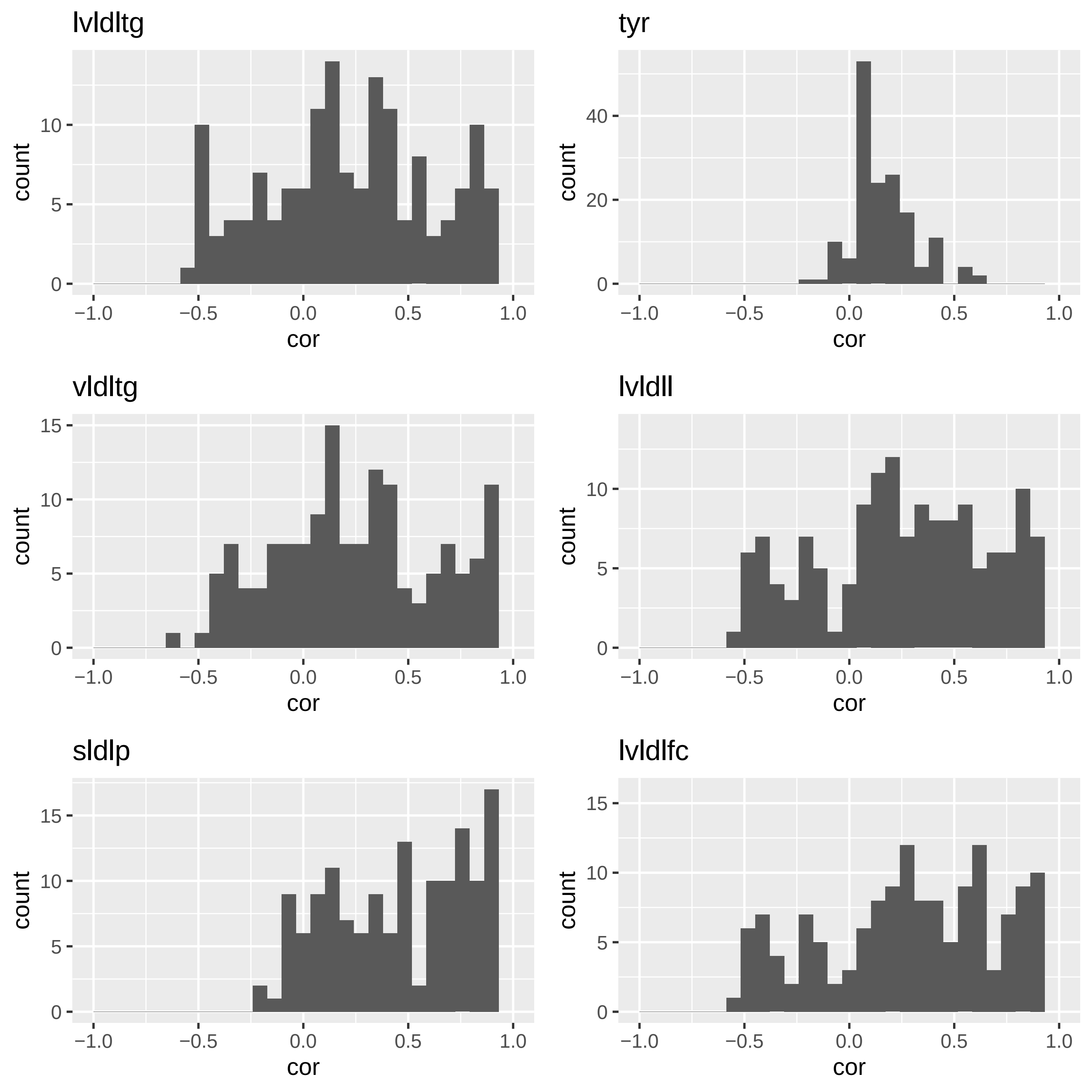
lvldltg
tyr
vldltg
lvldll
sldlp
lvldlfc
count
cor
0
5
10
15
20
40
−1.0
−0.5
0.0
0.5
1.0

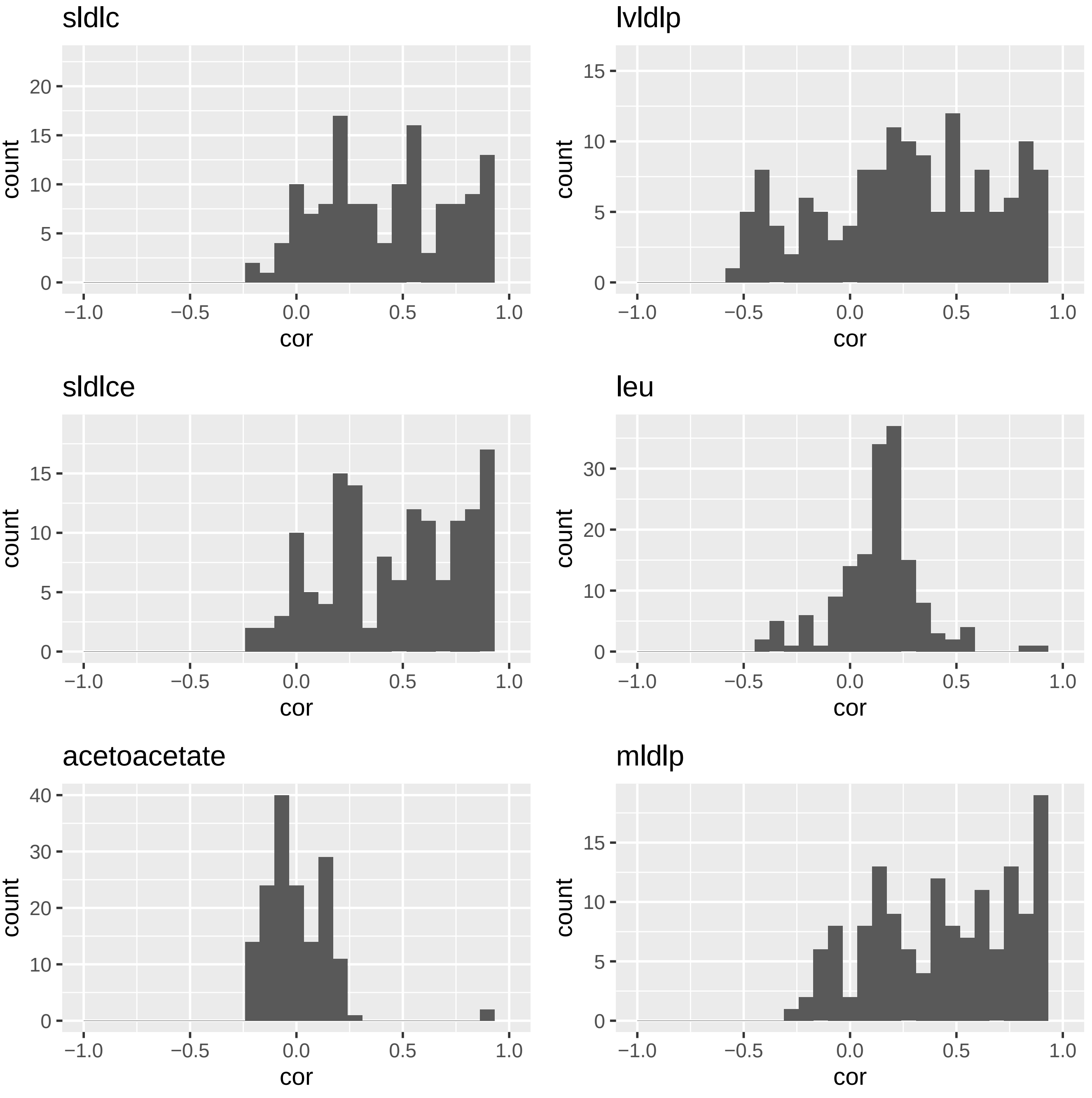
sldlc
lvldlp
sldlce
leu
acetoacetate
mldlp
count
cor
−1.0
−0.5
0.0
0.5
1.0
0
5
10
15
20
30
40

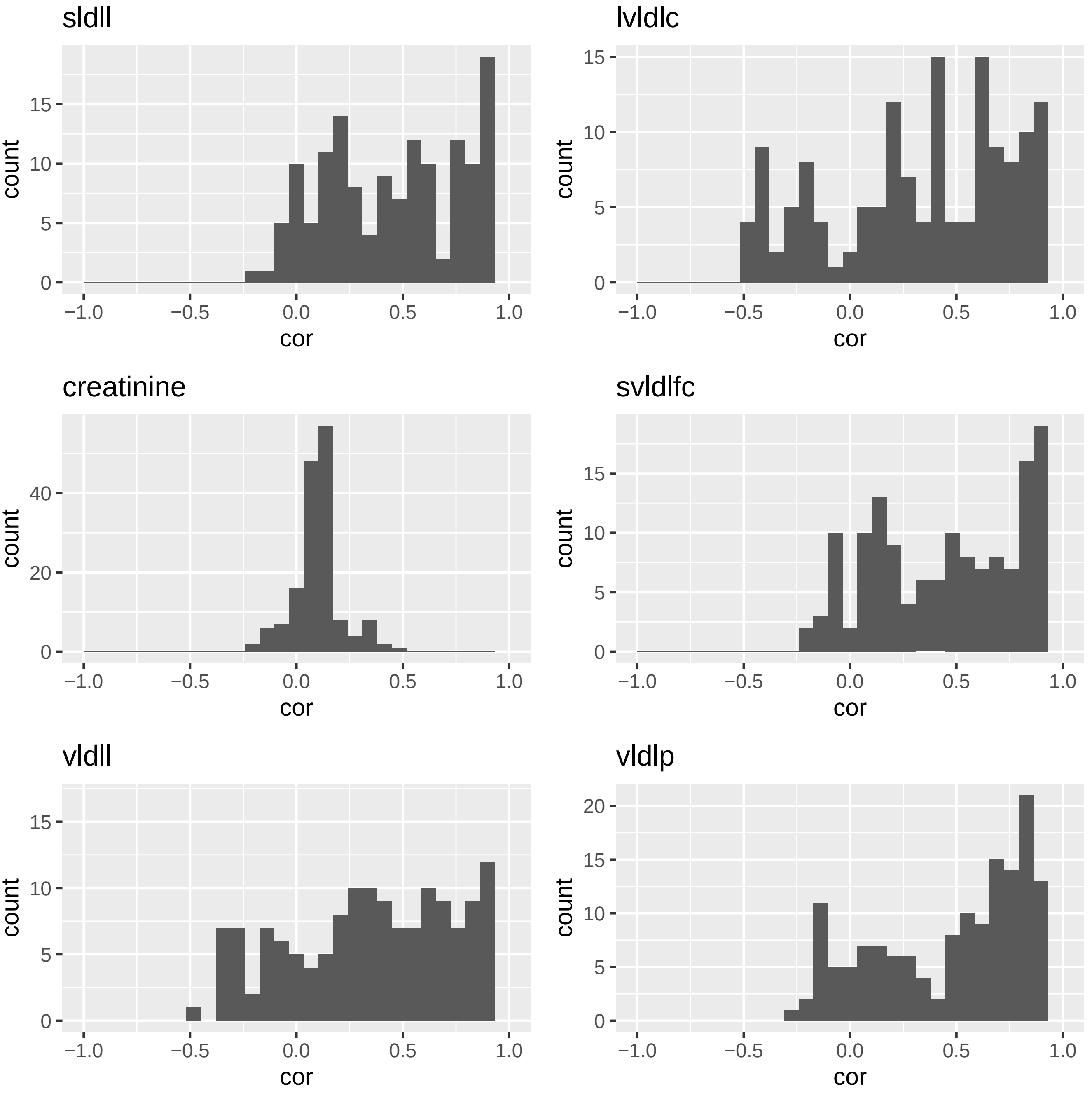

sldll
lvldlc
creatinine
svldlfc
vldll
vldlp
count
cor

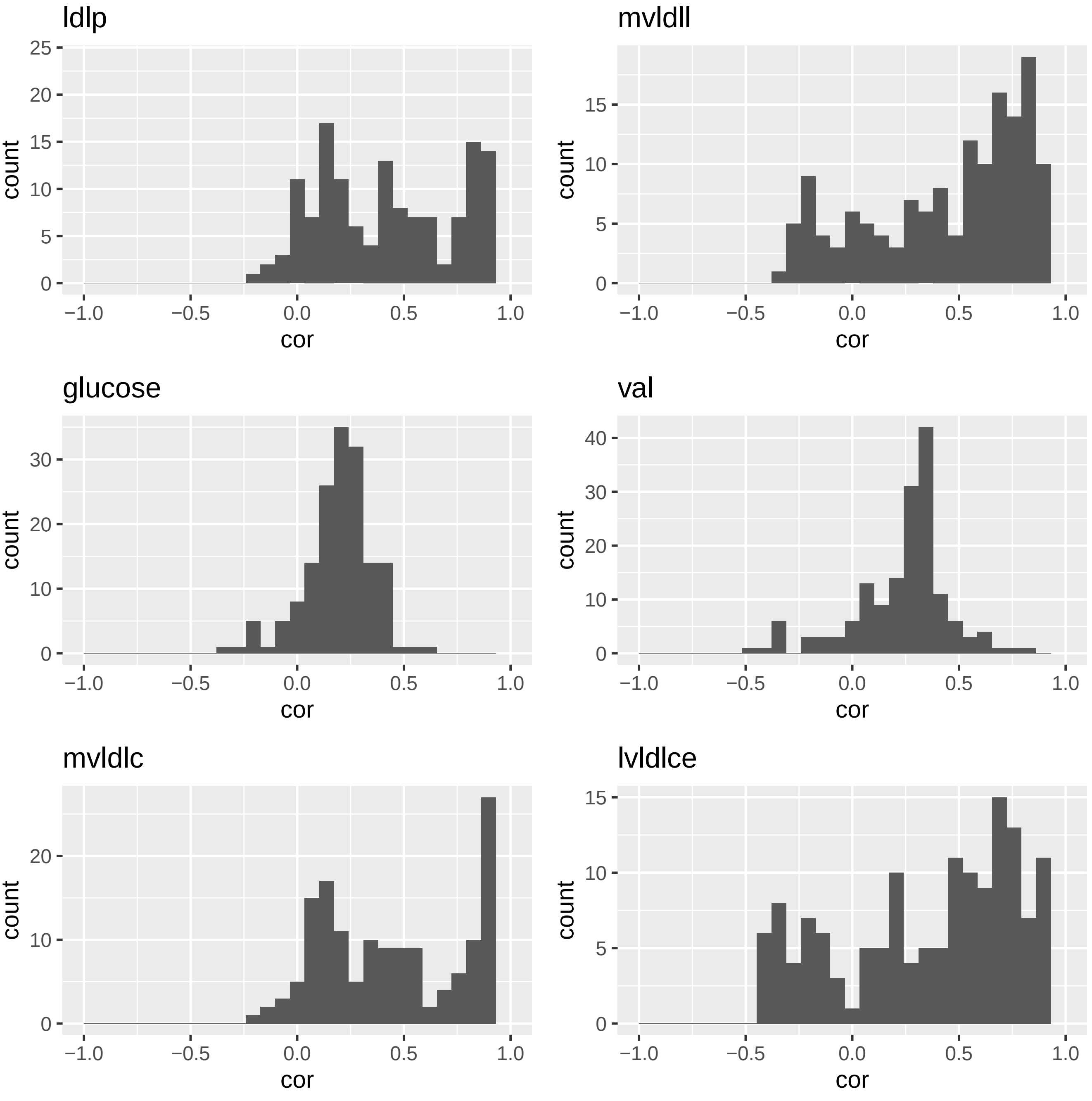
ldlp
mvldll
glucose
val
mvldlc
lvldlce
count
cor

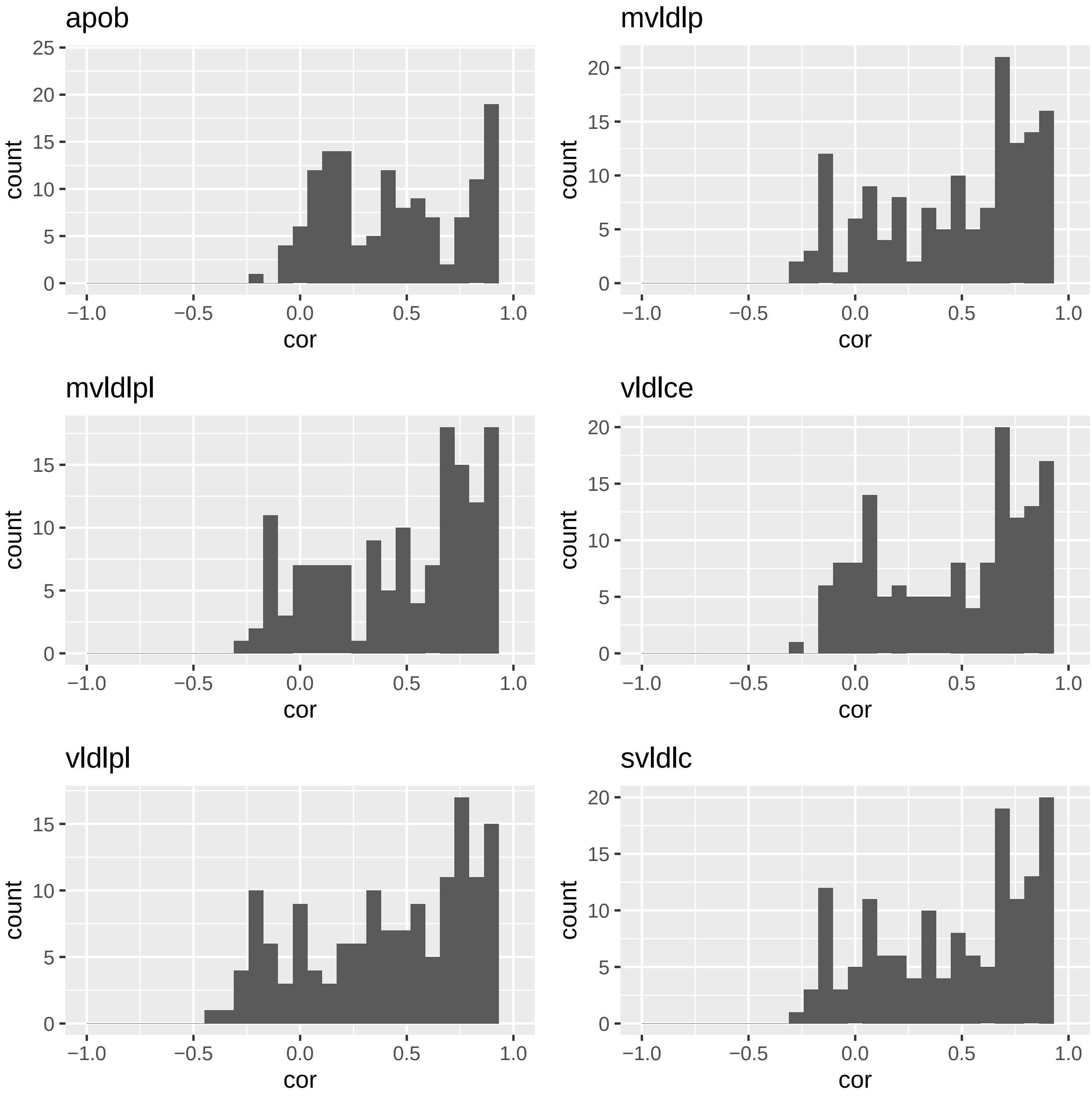
apob
mvldlp
mvldlpl
vldlce
vldlpl
svldlc
count
cor
−1.0
−0.5
0.0
0.5
1.0
0
5
10
15
20
25

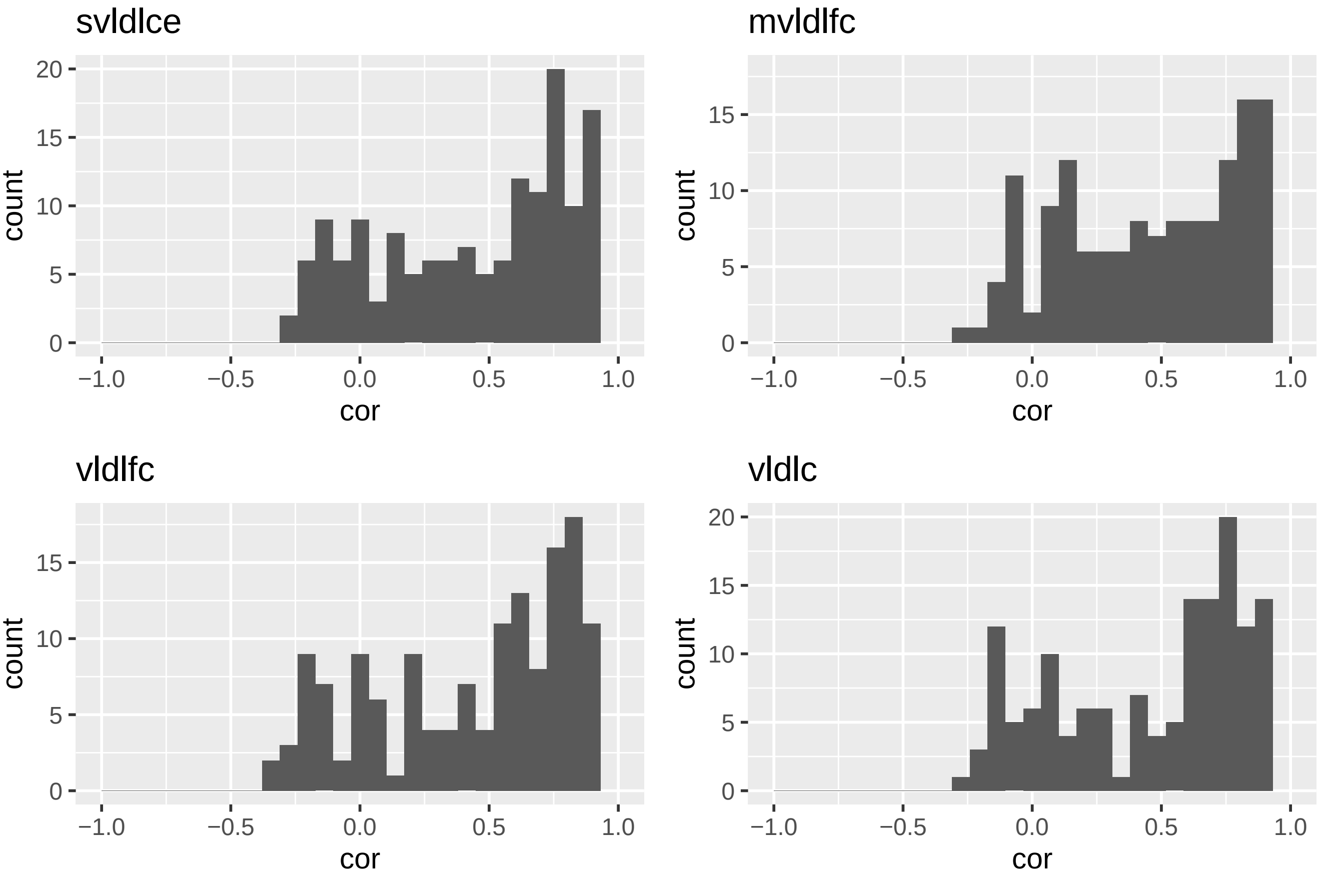
svldlce
mvldlfc
vldlfc
vldlc
count
cor
20
15
10
5
0
−1.0
−0.5
0.0
0.5
1.0
